# A Model Curriculum for an Emergency Medicine Residency Rotation in Clinical Informatics

**DOI:** 10.21980/J82P9H

**Published:** 2022-10-15

**Authors:** Carrie K Baker, Nivethietha Maniam, Benjamin H Schnapp, Nicholas Genes, Jeffrey A Nielson, Vishnu Mohan, William Hersh, Benjamin H Slovis

**Affiliations:** 1 Soin Medical Center, Kettering Health, Clinical Informatics Fellowship Program, Dayton OH; 2 Wright State University, Department of Emergency Medicine, Dayton, OH; 3 Thomas Jefferson University, Department of Medicine, Philadelphia, PA; 4 University of Wisconsin, Department of Emergency Medicine, Madison, WI; 5 New York University Grossman School of Medicine, Ronald O Perelman Department of Emergency Medicine, New York, NY; 6 Kettering Health, Department of Emergency Medicine, Dayton, OH; 7 Northeast Ohio Medical University, Department of Emergency Medicine, Rootstown, OH; 8 Oregon Health & Science University, Department of Medical Informatics & Clinical Epidemiology, Portland, OR; 9 Department of Medicine, Oregon Health & Science University, Portland Oregon; 10 Department of Emergency Medicine, Thomas Jefferson University, Philadelphia PA; 11 Office of Clinical Informatics, Thomas Jefferson University Hospitals, Philadelphia PA

**Keywords:** Informatics, Emergency Medicine, Curriculum, Education, Resident

## Abstract

**Audience:**

This curriculum is designed for emergency medicine residents at all levels of training. The curriculum covers basic foundations in clinical informatics for improving patient care and outcomes, utilizing data, and leading improvements in emergency medicine.

**Length of Curriculum:**

The curriculum is designed for a four-week rotation.

**Introduction:**

The American College of Graduate Medical Education (ACGME) mandated that all Emergency Medicine (EM) residents receive specific training in the use of information technology.^1^,^2^ To our knowledge, a clinical informatics curriculum for EM residents does not exist. We propose the following standardized and reproducible educational curriculum for EM residents.

**Educational Goals:**

The aim of this curriculum is to teach informatics skills to emergency physicians to improve patient care and outcomes, utilize data, and develop projects to lead change.^3^ These goals will be achieved by providing a foundational informatics elective for EM residents that follows the delineation of practice for Clinical Informatics outlined by the American Medical Informatics Association (AMIA) and the American Board of Preventive Medicine (ABPM).^4^–^6^

**Educational Methods:**

The educational strategies used in this curriculum include asynchronous learning via books, papers, videos, and websites. Residents attend administrative sessions (meetings), develop a project proposal, and participate in small group discussions.

The rotation emphasizes the basic concepts surrounding clinical informatics with an emphasis on improving care delivery and outcomes, information systems, data governance and analytics, as well as leadership and professionalism. The course focuses on the practical application of these concepts, including implementation, clinical decision support, workflow analysis, privacy and security, information technology across the patient care continuum, health information exchange, data analytics, and leading change through stakeholder engagement.

**Research Methods:**

An initial version of the curriculum was introduced to two separate institutions and was completed by three rotating resident physicians and one rotating resident pharmacist. A brief course evaluation as well as qualitative feedback was solicited from elective participants by the course director, via email following the completion of the course, regarding the effectiveness of the course content. Learner feedback was used to influence the development of this complete curriculum.

**Results:**

The curriculum was graded by learners on a 5-point Likert scale (1=strongly disagree, 5 = strongly agree). The mean response to, “This course was a valuable use of my elective time,” was 5 (sd=0). The mean response to, “I achieved the learning objectives,” and “This rotation helped me understand Clinical Informatics,” were both 4.75 (sd=0.5).

**Discussion:**

Overall, participants reported that the content was effective for achieving the learning objectives. During initial implementation, we found that the preliminary asynchronous learning component worked less effectively than we anticipated due to a lower volume of content. In response to this, as well as resident feedback, we added significantly more educational content.

In conclusion, this model curriculum provides a structured process for an informatics rotation for the emergency medicine resident that utilizes the core competencies established by the governing bodies of the clinical informatics specialty and ACGME.

**Topics:**

Clinical informatics key concepts, including definitions, fundamental terminology, history, policy and regulations, ethical considerations, clinical decision support, health information systems, data governance and analytics, process improvement, stakeholder engagement and change management.

## USER GUIDE

**Table:** 

**List of Resources:**	
Abstract	1
User Guide	3
Appendix A: Curriculum Timeline	10
Appendix B: Project Proposal Assignment	16
Appendix C: Sample Attendance Sheet and Time Log	17
Appendix D: Asynchronous Learning: Books, Papers, Videos, Websites	18
Appendix E.1: Small Group Discussion: Clinical Informatics Fundamentals	21
Appendix E.1.a: CI Fundamentals PPT	23
Appendix E.1.b: CI Fundamentals Instructor Material	24
Appendix E.1.c: CI Fundamentals Learner Material	26
Appendix E.2: Small Group Discussion: Improving Care Delivery and Outcomes	27
Appendix E.2.a: Care Delivery Outcomes CDS PPT	29
Appendix E.2.b: Care Delivery Outcomes CDS Form	30
Appendix E.3: Small Group Discussion: Data Analytics and Governance	32
Appendix E.3.a: Data Analytics Governance PPT	34
Appendix E.3.b: Data Analytics Governance Instructor Material	35
Appendix E.3.c: Data Analytics Governance Learner Material	39
Appendix E.4: Small Group Discussion: Leadership and Professionalism	42
Appendix E.4.a: Leadership PPT	44
Appendix E.4.b: Leadership Form	45
Appendix F: Sample Schedule	48
Appendix G: Sample Survey	50

**Learner Audience:**

Junior Residents, Senior Residents

**Length of Curriculum:**

The curriculum is designed for a four-week rotation. Residents have the option of continuing their project proposal longitudinally upon completion of the rotation, if approved.

**Topics:**

Clinical informatics key concepts, including definitions, fundamental terminology, history, policy and regulations, ethical considerations, clinical decision support, health information systems, data governance and analytics, process improvement, stakeholder engagement and change management.

**Objectives:**

Residents will gain an introduction of the broad field of clinical informatics, with a focus on the key applications of informatics in EM. By the end of this rotation, the learner will be able to:

1. State the value proposition of clinical informatics.
2. Describe the federal policies and legislation that influence the adoption of health information technology in the United States.
3. Propose ideas for planning, implementation and support necessary for the successful use of a clinical information system.
4. Explain the need for standards, clinical terminologies, and ontologies.
5. Analyze the roles of computerized provider order entry, clinical decision support systems, health information exchanges and interoperability as related to health care delivery.
6. Recognize the need for data governance and analytics.
7. Utilize informatics techniques to perform research and quality improvement projects.
8. Develop and present a realistic informatics-based project plan for a problem in their clinical environment, with the option to pursue further research or quality improvement.

### Brief introduction

As hospitals and health care providers increasingly rely on and interact with clinical information systems, there is a growing demand for formalized training in the field of clinical informatics. Clinical informaticians transform health care by analyzing, designing, implementing, and evaluating information and communication systems to enhance individual and population health outcomes, improve patient care, and strengthen the clinician-patient relationship.^3^,^4^

In 2011, the American Board of Medical Specialties approved Clinical Informatics as a subspecialty, recognizing the importance of information management in health care quality to promote patient care that is safe, equitable, efficient, effective, timely, and patient-centered.^4^,^7^,^8^ Recent updates to these core competencies maintain and promote the specialty’s position in the health care system.^5^,^6^

Successful optimization of health information systems depends on the knowledge and skills of the individuals who apply the core concepts, methods, and skills of clinical informatics. As residents enter an increasingly digital medical world, education must adapt to equip students with the necessary tools to further advancements in clinical care and health systems. It has been suggested that informatics should be a part of all formal medical education.^9^

While many medical schools and residencies have added educational content related to information retrieval and basic use of electronic health records, only a few have expanded their curricula to include the myriad of other ways physicians interact with electronic health information including clinical decision support, quality measurement and improvement, personal health records, telemedicine, and personalized medicine, despite established undergraduate medicine competencies and curricula.^10^,^11^ The use of health information technology permeates graduate medical practice, yet only a handful of residency programs and national organizations have designed, integrated, or recommended formalized informatics curriculum into their training programs.^12^–^17^

Informatics is especially relevant to the specialty and practice of Emergency Medicine (EM). The efficient management of information from the rapid processing, analysis, and interpretation of patient data in an emergent setting is crucial to patient outcomes. Through the widespread use of electronic health records feeding data to community resources, emergency physicians can monitor and better care for specific populations of patients, thus bridging the gap between personal and population health. The use of technology has become a requirement in EM training from both the American Board of Emergency Medicine (ABEM) Knowledge, Skills and Abilities (KSAs)^18^ and the Emergency Medicine Milestones Project, a joint venture between ABEM and the Accreditation Council for Graduate Medical Education (ACGME).^2^,^19^ Specifically, milestone Systems-Based Practice (SBP) 3 focuses on use of technology, while milestone SBP1 focuses on patient safety. Milestone SBP2 focuses on system-based management. Milestone Practice-Based Learning and Improvement (PBLI) focuses on the practice of evidence-based medicine, while Milestone Interpersonal and Communication Skills (ICS) 2 focuses on team management.^2^,^19^

Developing and updating resident competencies in informatics is essential for the future of EM as a specialty, yet informatics education in EM graduate training is often absent or inconsistent in the US and abroad.^20^,^21^ Currently, few options exist for residents to gain exposure to this field. Residents in departments that also host ACGME accredited fellowships may receive more exposure, but only four of these programs were documented in 2018 by the Emergency Medicine Residents Association (EMRA),^22^ though other clinical informatics programs offer EM tracks.^23^ There are also distance learning opportunities, including AMIA’s 10x10 course created by Oregon Health and Science University. One section of this course is held in conjunction with the American College of Emergency Physicians (ACEP) annually. However, these courses span many months and cost approximately $2,000, which may limit the number of EM residents who take the course.^24^ A residency curriculum in clinical informatics could help ensure the development of EM physicians who are able to use the tools of informatics to improve care for patients.

#### Curriculum Development Framework

Using Kern’s six-step approach to medical education curriculum development as a framework,^25^ we developed a curriculum for EM clinical informatics education that aids the next generation of emergency physicians in utilizing clinical informatics during a graduate medical education elective. We included a thematic breakdown of vital topics in EM informatics, as well as suggested content and readings.

### Problem identification, general and targeted needs assessment

The American College of Graduate Medical Education (ACGME) mandates that all EM residents receive specific training in the use of information technology.^2^,^19^ EM physicians routinely interact with clinical systems in their medical practice. The ABEM and the ACGME mandated that all EM residents receive specific training in the use of information technology to optimize learning, improve patient care, and accomplish and document safe health care delivery.^2^,^18^ Currently, a widely accessible, standardized informatics curriculum is not available to train EM residents. As a result, exposure to foundational clinical informatics remains inconsistent between training programs, leaving many EM residents with a deficit in training. We propose the following standardized and reproducible educational curriculum for EM residents.

### Goals of the curriculum

The aim of this curriculum is to teach informatics skills to emergency physicians to improve patient care and outcomes, utilize data, and develop leadership abilities. These goals will be achieved by providing a foundational informatics elective for EM residents that follows the delineation of practice for clinical informatics outlined by the American Medical Informatics Association (AMIA) and the American Board of Preventive Medicine (ABPM).^5^,^6^

### Objectives of the curriculum

Residents will gain an introduction of the broad field of clinical informatics, with a focus on the key applications of informatics in EM. By the end of this rotation, the learner will be able to:

1. State the value proposition of clinical informatics.
2. Describe the federal policies and legislation that influence the adoption of health information technology in the United States.
3. Propose ideas for planning, implementation and support necessary for the successful use of a clinical information system.
4. Explain the need for standards, clinical terminologies, and ontologies.
5. Analyze the roles of computerized provider order entry, clinical decision support systems, health information exchanges and interoperability as related to health care delivery.
6. Recognize the need for data governance and analytics.
7. Utilize informatics techniques to perform research and quality improvement projects.
8. Develop and present a realistic informatics-based project plan for a problem in their clinical environment, with the option to pursue further research or quality improvement.

### Educational Strategies

The curriculum will offer an introductory study of the basic concepts surrounding clinical information systems, focusing on the practical application of these concepts within the realm of EM. Asynchronous coursework will be combined with experiential learning to create a robust educational experience that can be replicated and implemented at a variety of EM residency training programs.

The curriculum fulfills the ACGME requirements pertaining to information technology for EM programs.^1^ These include:

- IV.B.1.d) Practice-Based Learning and Improvement
  ○ Residents must demonstrate the ability to investigate and evaluate their care of
  ○ patients, to appraise and assimilate scientific evidence, and to continuously
  ○ improve patient care based on constant self-evaluation and lifelong learning.
  ○ IV.B.1.d.(1) Residents must demonstrate competence in:
    ▪ IV.B.1.d).(1).(g) using information technology to optimize learning.
    ▪ IV.B.1.d).(1).(i) using information technology to improve patient care.
- IV.B.1.e) Interpersonal and Communication Skills
  ○ Residents must demonstrate interpersonal and communication skills that result in the effective exchange of information and collaboration with patients, their families, and health professionals.
  ○ IV.B.1.e).(1) Residents must demonstrate competence in:
    ▪ IV.B.1.e).(1).(f) maintaining comprehensive, timely, and legible medical records, if applicable.
- IV.B.1.f) Systems-based Practice
  ○ Residents must demonstrate an awareness of and responsiveness to the larger context and system of health care, including the social determinants of health, as well as the ability to call effectively on other resources to provide optimal health care.
  ○ IV.B.1.f).(1).(d) working in interprofessional teams to enhance patient safety and improve patient care quality.
  ○ IV.B.1.f).(1).(i) using technology to accomplish and document safe health care delivery.

Informatics is a rapidly developing field. We suggest the rotation director be an EM physician with experience in the field of clinical informatics, preferably board eligible or board certified. While the educational system is growing with certifications and fellowships, we do not feel these are necessary for the EM residency informatics rotation instructor. Many experts in the field acquired knowledge through experience and self-education. Most of the rotation will take place in the hospital setting. Learners should have access to the institutional health system with basic literacy in information retrieval.

The goal of this curriculum is for residents in EM to build basic competency in the field of informatics through education and clinical application. They will expand their knowledge beyond data access and retrieval. They should understand the broader role of health information technology (HIT) in health care delivery, public health and clinical research, and quality improvement. Clinical informaticians draw from the field of biomedicine and informatics to apply these tools to the practice of medicine. As per the AMIA white paper on Core Content for the Subspecialty of Clinical Informatics, clinical informatics encompasses three spheres of activity: clinical care, the health system, and information and communications technology. Through this elective, residents will gain a better understanding of the five major categories that form the core content of informatics: fundamentals, improving care delivery and outcomes, health information systems, data governance and data analytics, and leadership and professionalism.^4^–^6^

#### I. Fundamentals

The first core concept focuses on learning the fundamentals and basic knowledge needed for students to understand the environment in which an informaticist functions. This includes the history of informatics, key informatics concepts, models and theories, commonly cited literature, and an overview of the health system.^4^ The EM resident does not need to understand the entirety of clinical informatics but should appreciate its history, read several landmark articles, and understand the role that EM informaticists play in the emergency department and hospital system. This includes speaking the vocabulary within the culture of informatics as well as the technical and legislative developments that prompted evolution in the field. The resident will learn about policy and regulatory framework that led to the IT (Information Technology) application of coding guidelines and privacy laws of the 1990s, the creation of the Office of the National Coordinator of Health IT in 2004, the adoption of Health Information Technology for Economic and Clinical Health (HITECH) Act of 2009 to promote meaningful use, as well as the ongoing implementation of the 21^st^ Century Cures Act.^26^

Residents will acquire knowledge of the fundamentals of informatics through online resources, reading materials, participating in both department and hospital level operations and committee meetings as well as small group sessions. Additionally, residents will be encouraged to set up one-on-one meetings with university librarians to establish competency with medical literature database searches and information retrieval. They will also meet with Electronic Health Record (EHR) informatics analysts and/or clinical informaticists to learn the basics of their learning environment’s EHR, hospital information systems and leadership organization structure. After residents have a foundational knowledge of the key concepts of clinical informatics, they will then have an opportunity to apply these ideas to their clinical workflows. The fundamentals of clinical informatics align with milestones SBP3.^2^,^19^

#### II. Improving Care Delivery and Outcomes

Implementing and integrating clinical decision-making and care process improvement is the second core concept. One of the goals of this elective is for EM residents to understand their own decision-making through effective information analysis. Important concepts include evidence-based medicine (EBM), clinical decision support (CDS), and process redesign.^4^ While most students have a rudimentary understanding of clinical decision support and electronic health record capabilities, through independent readings and project assignments, they will understand how to optimize the system capabilities and become better advocates for patient care.

To accomplish this, they will review active and passive CDS in use in the ED, from order sets to interactive alerts, and gain an appreciation for the CDS maintenance processes. Furthermore, they will expand these skills to answer questions regarding individual, institutional, and community health practices. Residents will have the opportunity to apply informatics core concepts to identifiable gaps in the clinical setting and apply process redesign to address those problems. Improving Care Delivery and Outcomes aligns with milestones SBP1, SBP2, SBP3, PBL1 and ICS2.^2^,^19^

#### III. Health Information Systems

The third core concept focuses on health information systems and interoperability. Themes in this concept include computer programming, networks and databases, data security, clinical data standards and information system lifecycles.^4^ Information systems are complex networks encompassing people, processes, and technology. As residents move into new roles beyond training, they must not only know how to use health systems, but also how to evaluate the effectiveness of a system in meeting clinical goals. They need to be able to critically assess the advantages and disadvantages of technological tools and interpret the quality of data produced. Residents should not be expected to learn computer programming but instead appreciate the advantages and limitations of information systems and their associated software.

They will accomplish these objectives through independent readings and online tutorials.

The final project in our curriculum will allow residents to develop an informatics-based solution to a problem in their clinical environment. They will explain how they would implement an optimization or introduce a new workflow to their EHR, and develop measures of the effectiveness of that change, in terms of clinician behavioral change or patient outcomes. These skills enable residents to evaluate the reliability of data, provide feedback, and integrate their solution to provide better patient care.

#### IV. Data Governance and Data Analytics

The fourth concept focuses on standards of data governance, policies, and processes.^5^,^6^ Through readings and online didactics, residents will learn best practices for data use, privacy and security, as well as the “data life cycle” and need for data validation and management to optimize data sharing across systems.

EM residents will gain an understanding of the basic principles of analytics techniques, as well as machine learning, data visualization and natural language processing through independent reading and small group discussion. Residents will apply this knowledge in their final project, maintaining appropriate methodologies of data governance and utilizing their understanding of analytics to propose methods of assessment and interpretation of their described intervention.

#### V. Leadership and Professionalism

Finally, the fifth core concept focuses on the organization of health care institutions and techniques to effectively introduce change into these organizations. Themes include identifying and engaging with stakeholders, building interdisciplinary teams, and project management.^4^ As medicine becomes increasingly interdisciplinary, physicians must foster collaboration and effect change at the organizational level. It is useful to understand how informatics decisions for the ED can impact a diverse array of stakeholders – from patients and front-line staff to social work and case management, consultants, inpatient and outpatient clinicians, as well as regulatory and compliance, legal and financial stakeholders.

While a single rotation will not allow for full development of leadership skills, EM residents will experience some aspects of managing teams, effective communication, and group management processes. It is expected that residents will observe and understand administrative leadership roles by attending meetings with departmental and hospital leadership. Examples of administrative leadership meetings include the following: clinical decision support committee, computerized provider order management committee, quality and utilization committee, medical informatics committee, physician advisory council, analytics council, process excellence committee, and clinical pathways committee.

Residents will gain a basic understanding of leadership and professionalism through the lens of clinical informatics through reading and online video instruction. The residents will apply the knowledge they gained of the five core competencies to a project with an informatics-based solution. This will be based on a problem in their clinical setting. Ideas may include clinical decision support, hospital information systems, data analytics, and leadership skills to enact change.

Through this introduction to clinical informatics, we hope residents will gain a better understanding of the framework of information systems, including processes to enhance medical decision making and improve patient care. Demand will increase in the EM community to invest in informatics initiatives. It is our obligation to train students to optimize available tools and develop new solutions to improve the future of medicine.

#### Curriculum Design

This curriculum aims to provide a model for a structured informatics rotation for EM residents that fulfills ACGME requirements by following the Core Content for Clinical Informatics outlined by the American Medical Informatics Association (AMIA) and the American Board of Preventive Medicine (ABPM).^4^–^6^

The authors performed a PubMed (National Center for Biotechnology Information, Bethesda, MD) and Google Scholar search for terms associated with EM residency informatics curricula. We were unable to identify any published EM residency curricula in clinical informatics. Informatics curricula from other non-EM specialties, non-Graduate Medical Education (GME) courses, and medical student electives were adapted to meet the needs of an EM resident.^10^,^11^,^15^–^17^,^20^,^22^–^24^ Expert opinion on the fields of EM informatics and education were included for the curriculum design: authors Carrie Baker, Benjamin Slovis, Nicholas Genes and Jeffrey Nielson all hold leadership positions in ACEP with extensive experience in EM informatics education. Benjamin Schnapp is a medical education fellowship trained EM physician with experience in resident education and curriculum design. William Hersh and Vishnu Mohan are leaders in clinical informatics with numerous academic and educational contributions to the field.

### Results and tips for successful implementation

Four learners participated in an initial version of the curriculum and provided feedback via the standardized follow-up survey as well as comments on the curriculum. These results were used to further develop the curriculum into its current state. Surveys included a 5-point Likert scale with 1 indicating “strongly disagree” and 5 indicating “strongly agree.”

The mean response, “This course was a valuable use of my elective time,” was 5 (sd=0). The mean response to, “I achieved the learning objectives,” and “This rotation helped me understand Clinical Informatics,” were 4.75 (sd=0.5).

Free-form feedback from participants was also received. Comments included: “I got a good experience out of this rotation. I was able to get a good feeling for what an informatics fellowship could be like.” Rotators found that the leadership component was particularly effective, reporting “The exposure to the daily workload and workflow of the fellows greatly helped me to understand and appreciate the role of clinical informatics. Attending various meetings with the network’s informatics team allowed me to see the power of teamwork and quality improvement projects.” Another learner commented, “It showed me the general mindset and process of thinking as a clinical informaticist in how data is collected, processed, and applied in the clinical setting to take data and create knowledge. I also learned some of the terminology and language used in informatics which is important in understanding and communicating in the field.”

Targeted learners are either second or third-year EM residents who are expected to be in good standing with the residency program. Residents will have approximately four weeks to complete the curriculum, with asynchronous learning performed at their own pace. Didactic sessions and administrative sessions will be attended at set times based on the system, course director, and lecturer schedules. Residents may be given the opportunity to implement their proposal after completion of the rotation as a longitudinal project.

### Evaluation and Feedback

Residents will provide feedback in a standardized follow-up survey administered to each resident after the rotation. Additionally, residents are expected to present their project proposal to the appropriate governing committee for feedback (ED leadership, EM core faculty, Quality Improvement Committee, Project Management Office, Business Development, etc.) The leadership reviewers will provide feedback in a standardized follow-up survey.

Comments from the initial rotators influenced modifications to the curriculum including further development of small group discussion topics and increasing emphasis on operational informatics.

### Associated Content

Appendix A. Curriculum Chart
Appendix B. Project Proposal Assignment
Appendix C. Sample Attendance Sheet and Time Log
Appendix D. Asynchronous Learning: Books, Papers, Videos, Websites
Appendix E. Small Group Discussions
  Appendix E.1. Clinical Informatics (CI) Fundamentals
    Appendix E.1.a. CI Fundamentals PPT
    Appendix E.1.b. CI Fundamentals Instructor Materials
    Appendix E.1.c. CI Fundamentals Learner Materials
  Appendix E.2. Improving Care Delivery and Outcomes
    Appendix E.2.a. Care Delivery Outcomes CDS PPT
    Appendix E.2.b. Care Delivery Outcomes CDS Form
  Appendix E.3. Data Analytics and Governance
    Appendix E.3.a. Data Analytics Governance PPT
    Appendix E.3.b. Data Analytics Governance Instructor Material
    Appendix E.3.c. Data Analytics Governance Learner Material
  Appendix E.4. Leadership and Professionalism
    Appendix E.4.a. Leadership PPT
    Appendix E.4.b. Leadership Form
Appendix F. Sample Schedule
Appendix G. Sample Survey

## DIDACTICS AND HANDS-ON CURRICULUM

### Appendix E.1: Small Group Discussion: Clinical Informatics Fundamentals

#### Appendix E.1.a: CI Fundamentals PPT

Please see associated PowerPoint file

### Appendix E.2: Small Group Discussion: Improving Care Delivery and Outcomes

#### Appendix E.2.a: Care Delivery Outcomes CDS PPT

Please see associated PowerPoint file

### Appendix E.3: Small Group Discussion: Data Analytics and Governance

#### Appendix E.3.a: Data Analytics Governance PPT

Please see associated PowerPoint file

### Appendix E.4: Small Group Discussion: Leadership and Professionalism

#### Appendix E.4.a: Leadership PPT

Please see associated PowerPoint file
