# Supplementary material for: A Model Curriculum for an Emergency Medicine Residency Rotation in Clinical Informatics
Source: J Educ Teach Emerg Med. 2022 Oct 15;7(4):C1–C50. doi: 10.21980/J82P9H (PMC10332664; doi:10.21980/J82P9H)
Supplement: Supplementary file 1 [file JETem-7-4-C1-AppendixA.docx]

| Topic | Recommended Educational Strategy | Educational Content | Objectives | Learners | Timing, Resources Needed (Space, Instructors, Equipment, Citations of JETem pubs or other literature) | Recommended Assessment, Milestones Addressed |
| --- | --- | --- | --- | --- | --- | --- |
| Clinical Informatics Fundamentals | Asynchronous Learning,  Administrative Sessions,  Faculty Presentation,  Small Group Discussion,  Case-Based Learning, Flipped Classroom, Active Learning, Muddiest Point, Problem-Based Learning, Team-Based Learning, Commitment-Based Learning | What is Clinical Informatics?  Clinical Informatics in the Emergency Department  Hospital Clinical Information Systems | Residents will gain an introduction of the broad field of clinical informatics with a focus on the key applications of informatics in emergency medicine.  -Learners will describe clinical informatics.  -Learners will describe the history of the field including federal policies influencing the use of electronic health records.  -Learners will describe resources available to them for further development of their understanding of information retrieval and project management. | PGY 2-3 EM Residents | Computer, projector or large monitor for group discussion, access to asynchronous learning resources (See Appendix D).  Learners will spend approximately 40 hours per week on rotation performing asynchronous learning, attending administrative sessions, participating in group discussions, and presenting their work.  Depending on the level of experience and familiarity of the material to the instructor, pre-work times may vary.  Instructors need to plan an introductory overview session and host the small group discussions weekly. | Assessment:  Submission of  Appendix E.1.b. CI Fundamentals, Learner Materials,  Demonstration of understanding during small group discussion,  Attendance and Time Log.  Milestones: SBP2, SBP3, PBL1 |
| Improving Care Delivery and Outcomes | Asynchronous Learning,  Administrative Sessions,  Faculty Presentation,  Small Group Discussion,  Case-Based Learning, Flipped Classroom, Active Learning, Muddiest Point, Problem-Based Learning, Team-Based Learning, Commitment-Based Learning | What are Clinical Decision Support Systems?  -How are Clinical Decision Support Systems affecting Emergency Medicine? | Propose ideas for planning, implementation, and support necessary for the successful use of a clinical information system.  -Analyze the roles of computerized provider order entry, clinical decision support systems.  -Learners will explain the role of Clinical Decision Support Systems in the practice of emergency medicine and healthcare at large.  -Learners will analyze the positive and negative effects of decision support as well as unintended consequences. | PGY 2-3 EM Residents | Computer, projector or large monitor for group discussion, access to asynchronous learning resources (See Appendix D).  Learners will spend approximately 40 hours per week on rotation performing asynchronous learning, attending administrative sessions, participating in group discussions, and presenting their work.  Depending on the level of experience and familiarity of the material to the instructor, pre-work times may vary.  Instructors need to host the small group discussions weekly. | Assessment:  Submission of Appendix E.2.b. Care Delivery Outcomes CDS Development Form.  Demonstration of understanding during small group discussion.  Comprehension and assimilation of information into project proposal draft.  Attendance and Time Log.  Milestone: SBP2 |
| Health Information Systems, Data Analytics and Data Governance | Asynchronous Learning,  Administrative Sessions,  Faculty Presentation,  Small Group Discussion,  Case-Based Learning, Flipped Classroom, Active Learning, Muddiest Point, Problem-Based Learning, Team-Based Learning, Commitment-Based Learning | What are Hospital Information systems?  -Security  -Programming  What is data governance?  Why is it important to ensure data quality and meaning across settings?  How is data used to optimize clinical and business decision making? | Appreciate the advantages and limitations of information systems and their associated software.  -Propose ideas for planning, implementation and support necessary for the successful use of a clinical information system.  -Learn best practices for data use, privacy and appreciate the risk associated with security breaches and government recommendations on how to avoid them.  -Develop a basic understanding of the concepts of analytics, machine learning, data visualization and natural language processing.  Learners will review the IS Framework and gain a better understanding of hospital information systems.  Learners will appreciate the importance of Data Security in Healthcare.  Learners will appreciate the role of health information exchanges in the emergency department.  Learners will appreciate the concept of terminologies and their application in health information technology.  Learners will understand the need for Healthcare Data standards.  Learners will appreciate the concept of data analytics and its application in healthcare.  Learners will develop a basic understanding of the concepts of data analytics for ED needs, including techniques of “AI” / machine learning, and natural language processing.  Learners will understand strategies for data warehouse access and methods of conducting research and quality projects to improve ED care and operations.  Learners will appreciate the utility of health information exchange in the ED, as well as some of the challenges of interoperability, necessity of data standards, and tradeoffs involved with different consent models. | PGY 2-3 EM Residents | Computer, projector or large monitor for group discussion, access to asynchronous learning resources (See Appendix D).  Learners will spend approximately 40 hours per week on rotation performing asynchronous learning, attending administrative sessions, participating in group discussions and presenting their work.  Depending on the level of experience and familiarity of the material to the instructor, pre-work times may vary.  Instructors need to host the small group discussions. | Assessment:  Submission of Appendix E.3b. Data Analytics Governance Learner Material.  Demonstration of understanding during small group discussion,  Comprehension and assimilation of information into project proposal draft.  Attendance and Time Log.  Milestones: SBP2 and SBP3 |
| Leadership and Professionalism | Asynchronous Learning,  Administrative Sessions,  Faculty Presentation,  Small Group Discussion,  Case-Based Learning, Flipped Classroom, Active Learning, Muddiest Point, Problem-Based Learning, Team-Based Learning, Commitment-Based Learning | -How to enact change.  -How to promote an implementation and gain end-user buy-in. | Appreciate the role and need for project management, change management, and stakeholder engagement.  Learn the basics of team management and effective communication skills.  Appreciate the role emergency medicine physicians can play in departmental and hospital leadership.  Learners will appreciate the complexity of change management and implementation science.  Learners will understand the requirements needed to initiate and see through a project. | PGY 2-3 EM Residents | Computer, projector or large monitor for group discussion, access to asynchronous learning resources (See Appendix D).  Learners will spend approximately 40 hours per week on rotation performing asynchronous learning, attending administrative sessions, participating in group discussions, and presenting their work.  Depending on the level of experience and familiarity of the material to the instructor, pre-work times may vary.  Instructors need to host the small group discussions and create an opportunity for the learners to present their final project at a regular leadership meeting or as a stand-alone event. | Assessment:  Presentation of project proposal,  Demonstration of understanding during small group discussion,  Attendance and Time Log.  Sample Survey.  Milestone:  ICS2 |
