# Supplementary material for: A Model Curriculum for an Emergency Medicine Residency Rotation in Clinical Informatics
Source: J Educ Teach Emerg Med. 2022 Oct 15;7(4):C1–C50. doi: 10.21980/J82P9H (PMC10332664; doi:10.21980/J82P9H)
Supplement: Supplementary file 2 [file JETem-7-4-C1-AppendixB.docx]

Appendix B:

Project Proposal Assignment

Resident Name: _______________________

Rotation Dates: _______________________

Identify a problem or need. Explain why this exists and why improvements or interventions are required. Paint a picture of the current state of this issue. _______________________________________________________________________________________________________________________________________________________­­­­___________­­­­___________­­­­_______

Describe your proposed intervention and ideal future state. Identify critical elements of your design, including how you will evaluate the current state of the problem, stakeholders involved, data sources, and plan for change. _______________________________________________________________________________________________________________________________________________________­­­­___________­­­­___________­­­­_______

Describe the key performance indicators and measurements used to determine success of your intervention. What data would you like to have?

_______________________________________________________________________________________________________________________________________________________­­­­___________­­­­___________­­­­_______

Describe how you will implement your intervention. Who are the key stakeholders and subject matter experts? How will you generate buy-in and utilization? How will you mitigate dissent? Develop a communication plan.

_______________________________________________________________________________________________________________________________________________________­­­­___________­­­­___________­­­­_______
