# Supplementary material for: A Model Curriculum for an Emergency Medicine Residency Rotation in Clinical Informatics
Source: J Educ Teach Emerg Med. 2022 Oct 15;7(4):C1–C50. doi: 10.21980/J82P9H (PMC10332664; doi:10.21980/J82P9H)
Supplement: Supplementary file 3 [file JETem-7-4-C1-AppendixC.docx]

Appendix C:

Sample Attendance Sheet and Time Log

Resident Name: _______________________ Rotation Block Dates: _______________________

|  | **Description:** | **Date** | **Faculty/Admin**  **Print** | **Faculty/Admin**  **Signature** |
| --- | --- | --- | --- | --- |
| **Administrative Sessions** | Organizational Leadership Meeting Examples: |  |  |  |
|  | Clinical Decision Support |  |  |  |
|  | Computerized Provider Order Management |  |  |  |
|  | Quality & Utilization |  |  |  |
|  | Medical Informatics |  |  |  |
|  | Physician Advisory Council |  |  |  |
|  | Analytics Council |  |  |  |
|  | Process Excellence |  |  |  |
|  | Clinical Pathways |  |  |  |
| **Asynchronous Learning** |  |  |  |  |
|  | Fundamentals |  |  |  |
|  | Care Delivery |  |  |  |
|  | IS/Data |  |  |  |
|  | Leadership |  |  |  |
| **Small Group Discussion** |  |  |  |  |
|  | Fundamentals |  |  |  |
|  | Care Delivery |  |  |  |
|  | IS / Data |  |  |  |
|  | Leadership |  |  |  |
| **Presentation:** |  |  |  |  |
