# Supplementary material for: A Model Curriculum for an Emergency Medicine Residency Rotation in Clinical Informatics
Source: J Educ Teach Emerg Med. 2022 Oct 15;7(4):C1–C50. doi: 10.21980/J82P9H (PMC10332664; doi:10.21980/J82P9H)
Supplement: Supplementary file 4 [file JETem-7-4-C1-AppendixD.docx]

Appendix D:

Asynchronous Learning: Books, Papers, Videos, Websites

**Books**

1. Finnell JT, Dixon BE, eds. *Clinical Informatics Study Guide.* 2nd ed. Springer; 2022.
   1. Ozkaynak M, Unertl KM, Johnson SA, Brixey JJ, Haque SN. Clinical Workflow Analysis, Process Redesign and Quality Improvement. In: Finnell JT, Dixon BE, eds. *Clinical Informatics Study Guide.* 2^nd^ ed. Springer; 2022:103-118.
   2. Kasthurirathne SN, Grannis SJ. Analytics. In: Finnell JT, Dixon BE, eds. *Clinical Informatics Study Guide.* Springer; 2022: 227-239.
   3. Dixon BE, Holmgren AJ, Adler-Milstein J, Grannis SJ. Health Information Exchange and Interoperability. In: Finnell JT, Dixon BE, eds. *Clinical Informatics Study Guide.* Springer; 2022:203-219.
   4. Schleyer T, Zappone S, Wells-Meyers C, Saxton T. Effective Interdisciplinary Teams. In: Finnell JT, Dixon BE, eds. *Clinical Informatics Study Guide.* 2nd ed. Springer; 2022: 285-306.
2. Hersh WR, ed. *Health Informatics: Practical Guide*. 8^th^ ed. Informatics Education. 2022.
   1. Hersh WR. Introduction to Biomedical and Health Informatics. In: Hersh WR, ed. *Health Informatics: Practical Guide*. 8^th^ ed. Informatics Education; 2022:1-18.
   2. Hersh WR. A Short History of Biomedical and Health Informatics. In: Hersh WR, ed. *Health Informatics: Practical Guide*. 8th ed. Informatics Education; 2022:19-26.
   3. Hersh WR. Evidence-Based Medicine. In: Hersh WR, ed. *Health Informatics: Practical Guide*. 8th ed. Informatics Education. 2022:377-397.
   4. Hersh WR. Data Science, Machine Learning, and Artificial Intelligence. In: Hersh WR, ed. *Health Informatics: Practical Guide*. 8^th^ ed. Informatics Education. 2022:89-115.
   5. Jenders RA. Clinical Decision Support. In: Hersh WR, ed. *Health Informatics: Practical* Guide. 8^th^ ed. Informatics Education. 2022:117-130.
   6. Rasmussen J. Health Information Privacy and Security. In: Hersh WR, ed. *Health Informatics: Practical Guide*. 8^th^ ed. Informatics Education. 2022:233-252.

**Reference Books Suggested for Further Reading:**

1. Levick D, Saldana L, Osheroff JA, eds. Healthcare Information and Management Systems Society. *Improving Outcomes with Clinical Decision Support: An Implementer’s Guide*. 2nd ed. HIMSS; 2012.
2. Shortiliffe EH, Cimino JJ, eds. *Biomedical Informatics: Computer Applications in Health Care and Biomedicine. 4^th^ Ed. London: Springer; 2014.*

**Papers**

1. Friedman CP. A "fundamental theorem" of biomedical informatics. *J Am Med Inform Assoc*. 2009;16(2):169-170. doi:10.1197/jamia.M3092
2. Bates DW, Kuperman GJ, Wang S, et al. Ten commandments for effective clinical decision support: making the practice of evidence-based medicine a reality. *J Am Med Inform Assoc*. 2003;10(6):523-530. doi:10.1197/jamia.M1370
3. Hripcsak G, Clayton PD, Jenders RA, Cimino JJ, Johnson SB. Design of a clinical event monitor. *Comput Biomed Res*. 1996;29(3):194-221. doi:10.1006/cbmr.1996.0016
4. Han YY, Carcillo JA, Venkataraman ST, et al. Unexpected increased mortality after implementation of a commercially sold computerized physician order entry system [published correction appears in *Pediatrics*. 2006 Feb;117(2):594]. *Pediatrics*. 2005;116(6):1506-1512. doi:10.1542/peds.2005-1287
5. Farley HL, Baumlin KM, Hamedani AG, et al. Quality and safety implications of emergency department information systems. *Ann Emerg Med*. 2013;62(4):399-407. doi:10.1016/j.annemergmed.2013.05.019
6. Melnick ER, Genes NG, Chawla NK, Akerman M, Baumlin KM, Jagoda A. Knowledge translation of the American College of Emergency Physicians' clinical policy on syncope using computerized clinical decision support. *Int J Emerg Med*. 2010;3(2):97-104. Published 2010 Jun 1. doi:10.1007/s12245-010-0168-x
7. Weingart SN, Simchowitz B, Padolsky H, et al. An empirical model to estimate the potential impact of medication safety alerts on patient safety, health care utilization, and cost in ambulatory care. *Arch Intern Med*. 2009;169(16):1465-1473. doi:10.1001/archinternmed.2009.252
8. Terrell KM, Perkins AJ, Hui SL, Callahan CM, Dexter PR, Miller DK. Computerized decision support for medication dosing in renal insufficiency: a randomized, controlled trial. *Ann Emerg Med*. 2010;56(6):623-629. doi:10.1016/j.annemergmed.2010.03.025
9. Powers EM, Shiffman RN, Melnick ER, Hickner A, Sharifi M. Efficacy and unintended consequences of hard-stop alerts in electronic health record systems: a systematic review. *J Am Med Inform Assoc*. 2018;25(11):1556-1566. doi:10.1093/jamia/ocy112
10. Strom BL, Schinnar R, Aberra F, et al. Unintended effects of a computerized physician order entry nearly hard-stop alert to prevent a drug interaction: a randomized controlled trial. *Arch Intern Med*. 2010;170(17):1578-1583. doi:10.1001/archinternmed.2010.324
11. Shapiro JS, Crowley D, Hoxhaj S, et al. Health Information Exchange in Emergency Medicine. *Ann Emerg Med*. 2016;67(2):216-226. doi:10.1016/j.annemergmed.2015.06.018
12. Cimino JJ. Desiderata for controlled medical vocabularies in the twenty-first century. *Methods Inf Med*. 1998;37(4-5):394-403.
13. Varkey P, Reller MK, Resar RK. Basics of quality improvement in health care. *Mayo Clin Proc*. 2007;82(6):735-739. doi:10.4065/82.6.735

**Videos**

1. Baker M, Slovis BH, Kring R. What is Clinical Informatics? ACEP.org. Published April 11, 2022. Accessed April 22, 2022. At: https://www.acep.org/administration/quality/health-information-technology/hit-articles/what-is-clinical-informatics/
2. Hersh WR. What is Biomedical and Health Informatics? (1). https://dmice.ohsu.edu/hersh/whatis/ Updated Jan 5, 2022. Accessed April 13, 2022. At: https://echo360.org/media/2c348b8a-fb1f-4689-a5fe-985faec1eebe/public
3. Hersh WR. What is Biomedical and Health Informatics? (2). https://dmice.ohsu.edu/hersh/whatis/ Updated Jan 5, 2022. Accessed April 13, 2022. At: https://echo360.org/media/66a38a82-9ad2-4391-90b5-9d1b3bc1db91/public
4. Hersh WR. A Short History of Biomedical and Health Informatics. https://dmice.ohsu.edu/hersh/whatis/ Updated Jan 5, 2022. Accessed April 13, 2022. At: https://echo360.org/media/cc1c9af1-94c5-4b62-be4d-6cf64ae6efa5/public
5. Hersh WR. Resources for Field: Organizations, Information, Education. https://dmice.ohsu.edu/hersh/whatis/ Updated Jan 5, 2022. Accessed April 13, 2022. At: https://echo360.org/media/6e2092b3-35b7-47f9-a0aa-40d3cba9cda8/public
6. Hersh WR. Clinical Data. <https://dmice.ohsu.edu/hersh/whatis/> Updated Jan 5, 2022. Accessed April 13, 2022. At: https://echo360.org/media/630e4d75-0024-4753-b9f2-a7475fbeff4f/public
7. Genes N. Digital Medicine for the Emergency Physician. ACEP Section for Emergency Medicine Informatics. Published 2016. Accessed April 13, 2022. At: https://www.acep.org/administration/quality/health-information-technology/hit-articles/digital-medicine-for-the-emergency-physician/
8. McClay J. FHIR. ACEP Section for Emergency Medicine Informatics, Grand Rounds. Published Aug 2, 2016. Accessed April 12, 2022. At: https://www.acep.org/administration/quality/health-information-technology/hit-articles/fhir-fast-health-information-resources/
9. Sivers D. How to start a movement. Ted.com. Feb 2010. Accessed April 12, 2022. At: https://www.ted.com/talks/derek_sivers_how_to_start_a_movement?language=en#t-169239

**Websites**

1. Campbell RJ. The Five Rights of Clinical Decision Support: CDS Tools Helpful for Meeting Meaningful Use. Published online October 2013. Accessed April 1, 2022. At: https://library.ahima.org/doc?oid=300027#.YlXJ9t-M4uU
2. U.S. Department of Health and Human Services: Office for Civil Rights. Breach Portal: Notice to the Secretary of HHS Breach of Unsecured Protected Health Information. Accessed April 12, 2022. At: https://ocrportal.hhs.gov/ocr/breach/breach_report.jsf
3. U.S. Department of Health & Human Services: Health Information Privacy. Cybersecurity Guidance Material. Accessed April 12, 2022. At: https://www.hhs.gov/hipaa/for-professionals/security/guidance/cybersecurity/index.html
4. Office of the National Coordinator for Health IT: Health IT Privacy and Security for Providers. Top 10 Tips for Cybersecurity in Health Care. Accessed April 12, 2022. At: https://www.healthit.gov/sites/default/files/Top_10_Tips_for_Cybersecurity.pdf
