# Supplementary material for: A Model Curriculum for an Emergency Medicine Residency Rotation in Clinical Informatics
Source: J Educ Teach Emerg Med. 2022 Oct 15;7(4):C1–C50. doi: 10.21980/J82P9H (PMC10332664; doi:10.21980/J82P9H)
Supplement: Supplementary file 5 [file JETem-7-4-C1-AppendixE1a.docx]

Appendix E.1:

Small Group Discussion: Clinical Informatics Fundamentals

**Pre-Session Preparation:**

Clinical informatics fundamentals will be reviewed at the beginning of the first week of a four-week rotation. Faculty will review asynchronous materials, “Appendix E.1.a. CI Fundamentals PPT,” and “Appendix E.1.b. CI Fundamentals Instructor Material.”

Learners may review the readings and video content asynchronously for an estimated time commitment of 10 hours. The small group session consists of a 20-minute lecture using “Appendix E.1.a. CI Fundamentals PPT,” and then a 40-minute small group question and answer sheet, “Appendix E.1.c. CI Fundamentals Learner Material.”

**Recommended Pre-Reading:**

1. Hersh WR, ed. *Health Informatics: Practical Guide*. 8^th^ ed
   - 1. Hersh WR. Introduction to Biomedical and Health Informatics. In: Hersh WR, ed. *Health Informatics: Practical Guide*. 8^th^ ed. Informatics Education. 2022:1-18.
     2. Hersh WR. A Short History of Biomedical and Health Informatics. In: Hersh WR, ed. *Health Informatics: Practical Guide*. 8th ed. Informatics Education. 2022:19-26.
     3. Hersh WR. Evidence-Based Medicine. In: Hersh WR, ed. *Health Informatics: Practical Guide*. 8th ed. Informatics Education. 2022:377-397.
2. Schleyer T, Zappone S, Wells-Meyers C, Saxton T. Effective Interdisciplinary Teams. In: Finnell JT, Dixon BE, eds. *Clinical Informatics Study Guide.* 2nd ed. Springer; 2022: 285-306.
3. Hersh WR. What is Biomedical and Health Informatics? (1). https://dmice.ohsu.edu/hersh/whatis/ Updated Jan 5, 2022. Accessed April 13, 2022. At: https://echo360.org/media/2c348b8a-fb1f-4689-a5fe-985faec1eebe/public
4. Hersh WR. What is Biomedical and Health Informatics? (2). https://dmice.ohsu.edu/hersh/whatis/ Updated Jan 5, 2022. Accessed April 13, 2022. At: https://echo360.org/media/66a38a82-9ad2-4391-90b5-9d1b3bc1db91/public
5. Hersh WR. A Short History of Biomedical and Health Informatics. https://dmice.ohsu.edu/hersh/whatis/ Updated Jan 5, 2022. Accessed April 13, 2022. At: https://echo360.org/media/cc1c9af1-94c5-4b62-be4d-6cf64ae6efa5/public
6. Hersh WR. Resources for Field: Organizations, Information, Education. https://dmice.ohsu.edu/hersh/whatis/ Updated Jan 5, 2022. Accessed April 13, 2022. At: https://echo360.org/media/6e2092b3-35b7-47f9-a0aa-40d3cba9cda8/public
7. Baker M, Slovis BH, Kring R. What is Clinical Informatics? ACEP.org. Published April 11, 2022. Accessed April 22, 2022. At: https://www.acep.org/administration/quality/health-information-technology/hit-articles/what-is-clinical-informatics/
8. Friedman CP. A "fundamental theorem" of biomedical informatics. *J Am Med Inform Assoc*. 2009;16(2):169-170. doi:10.1197/jamia.M3092

**Objectives:**

Residents will gain an introduction of the broad field of clinical informatics, with a focus on the key applications of informatics in EM. By the end of this small group section, the learner will be able to:

1. State the value proposition of clinical informatics.
2. Describe the federal policies and legislation that influence the adoption of health information technology in the United States.

**Linked objectives and methods:**

Objectives are achieved through small group discussion with guidance from the small group instructor. This allows for knowledge translation in an informal setting. Learners discuss their experiences and ideas in an open format.
