# Supplementary material for: A Model Curriculum for an Emergency Medicine Residency Rotation in Clinical Informatics
Source: J Educ Teach Emerg Med. 2022 Oct 15;7(4):C1–C50. doi: 10.21980/J82P9H (PMC10332664; doi:10.21980/J82P9H)
Supplement: Supplementary file 6 — Please see associated PowerPoint file [file JETem-7-4-C1-AppendixE1b.pptx]

## Slide 1
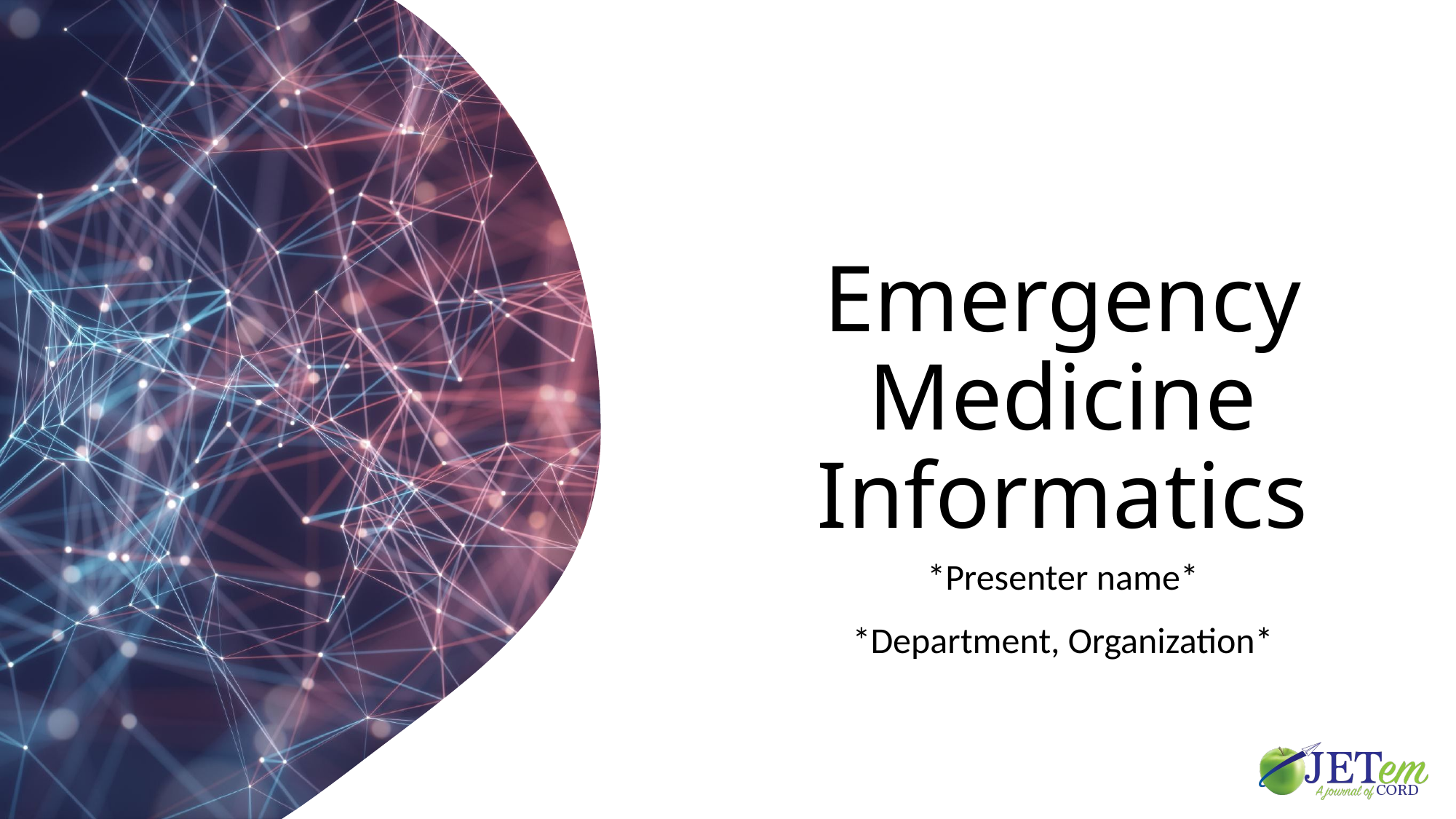

# Emergency Medicine Informatics
*Presenter name*
*Department, Organization*

## Slide 2
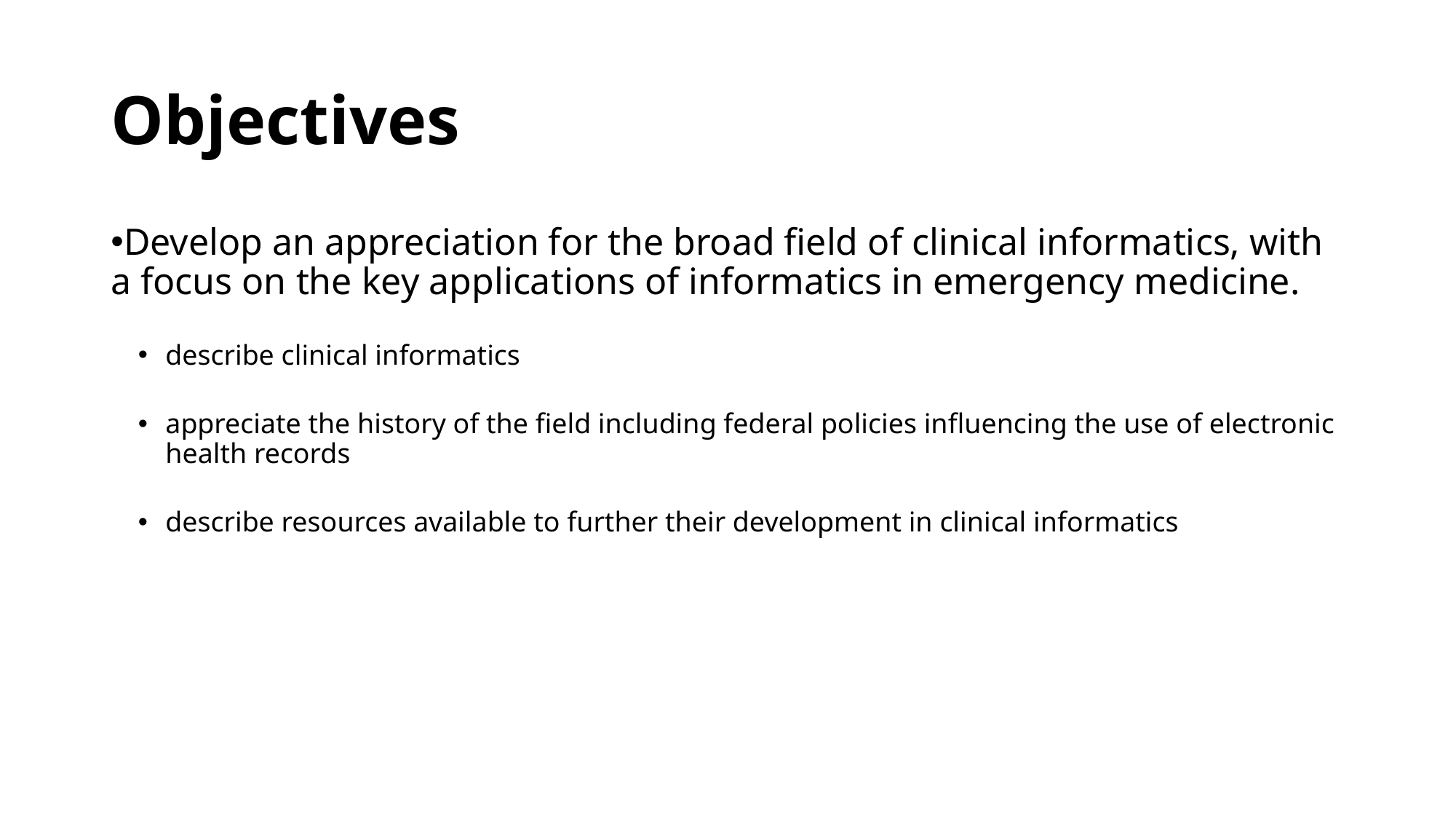

# Objectives
Develop an appreciation for the broad field of clinical informatics, with a focus on the key applications of informatics in emergency medicine.
describe clinical informatics
appreciate the history of the field including federal policies influencing the use of electronic health records
describe resources available to further their development in clinical informatics

## Slide 3
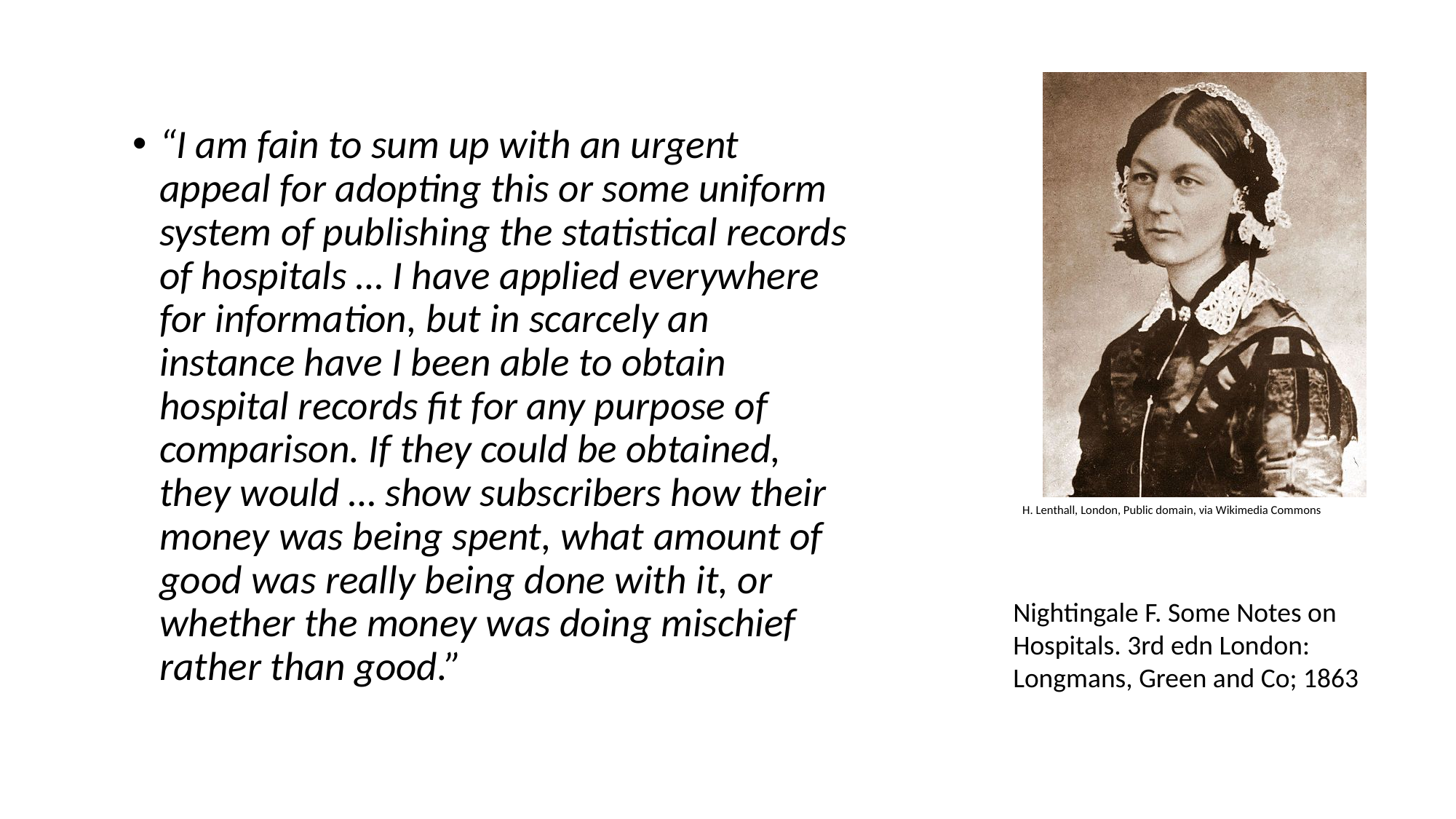

“I am fain to sum up with an urgent appeal for adopting this or some uniform system of publishing the statistical records of hospitals … I have applied everywhere for information, but in scarcely an instance have I been able to obtain hospital records fit for any purpose of comparison. If they could be obtained, they would … show subscribers how their money was being spent, what amount of good was really being done with it, or whether the money was doing mischief rather than good.”
H. Lenthall, London, Public domain, via Wikimedia Commons
Nightingale F. Some Notes on Hospitals. 3rd edn London: Longmans, Green and Co; 1863

## Slide 4
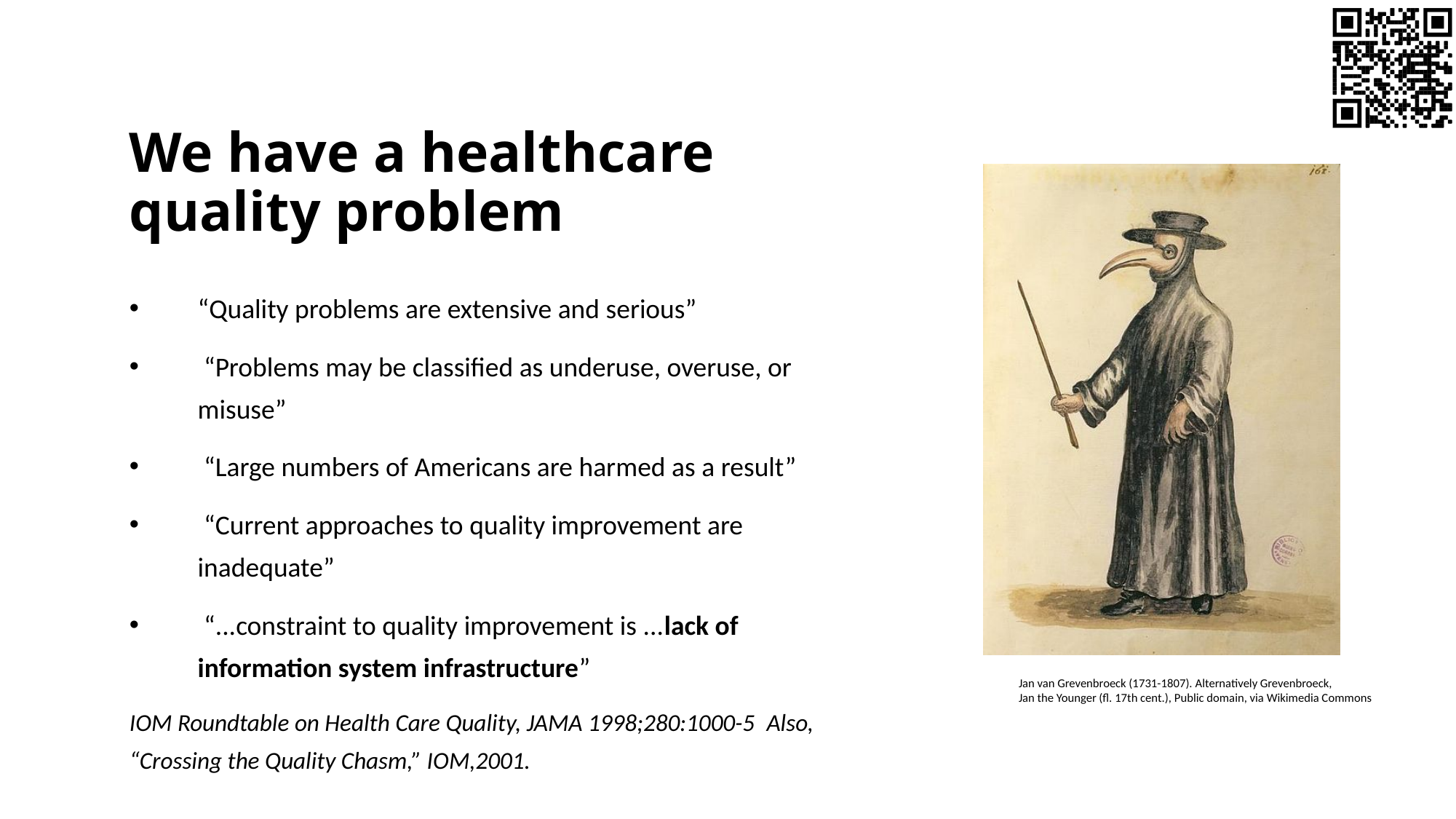

# We have a healthcare quality problem
“Quality problems are extensive and serious”
 “Problems may be classified as underuse, overuse, or misuse”
 “Large numbers of Americans are harmed as a result”
 “Current approaches to quality improvement are inadequate”
 “...constraint to quality improvement is ...lack of information system infrastructure”
IOM Roundtable on Health Care Quality, JAMA 1998;280:1000-5 Also, “Crossing the Quality Chasm,” IOM,2001.
Jan van Grevenbroeck (1731-1807). Alternatively Grevenbroeck,
Jan the Younger (fl. 17th cent.), Public domain, via Wikimedia Commons

## Slide 5
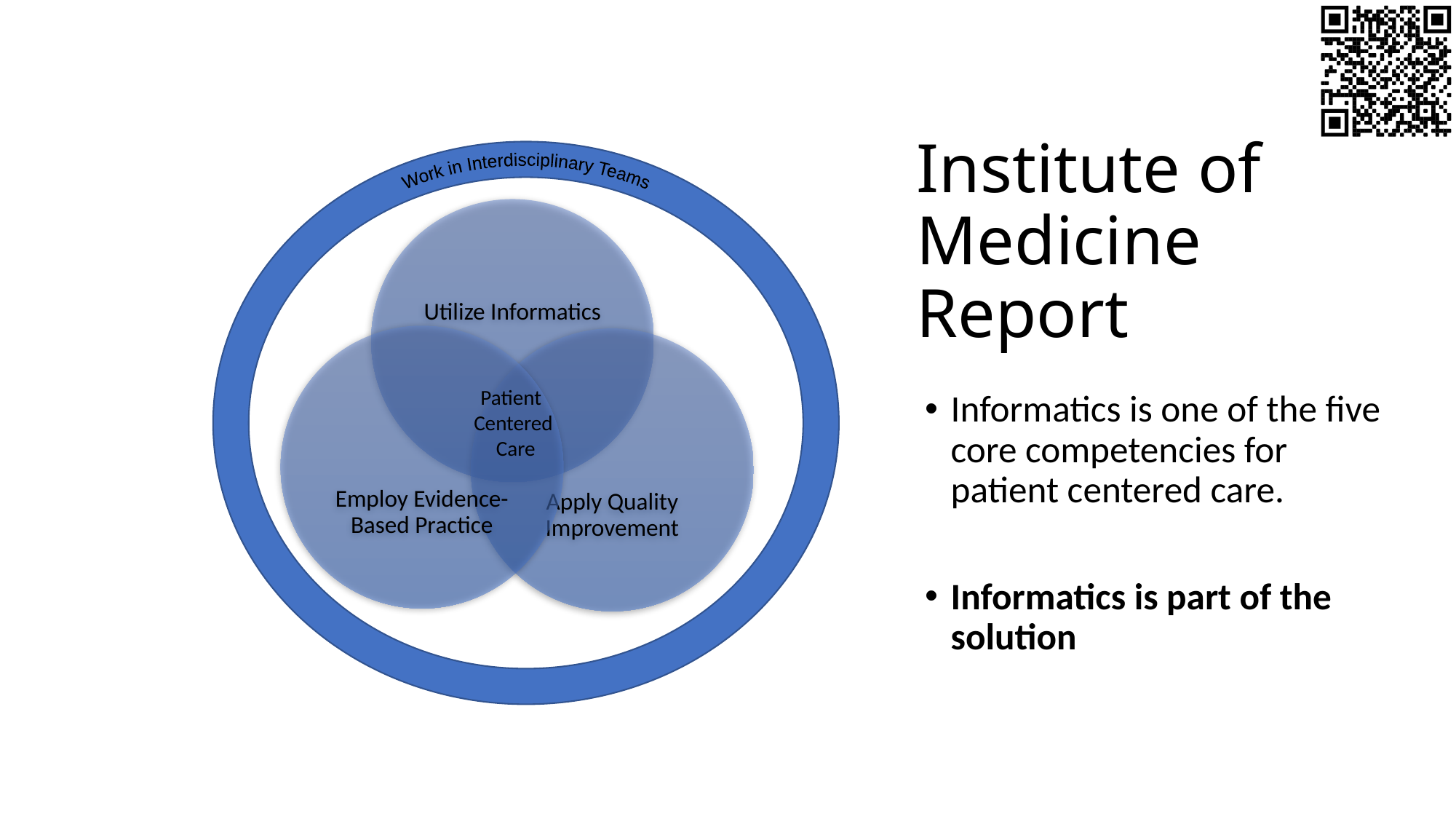

# Institute of Medicine Report
Work in Interdisciplinary Teams
Patient
Centered
 Care
Informatics is one of the five core competencies for patient centered care.
Informatics is part of the solution

## Slide 6
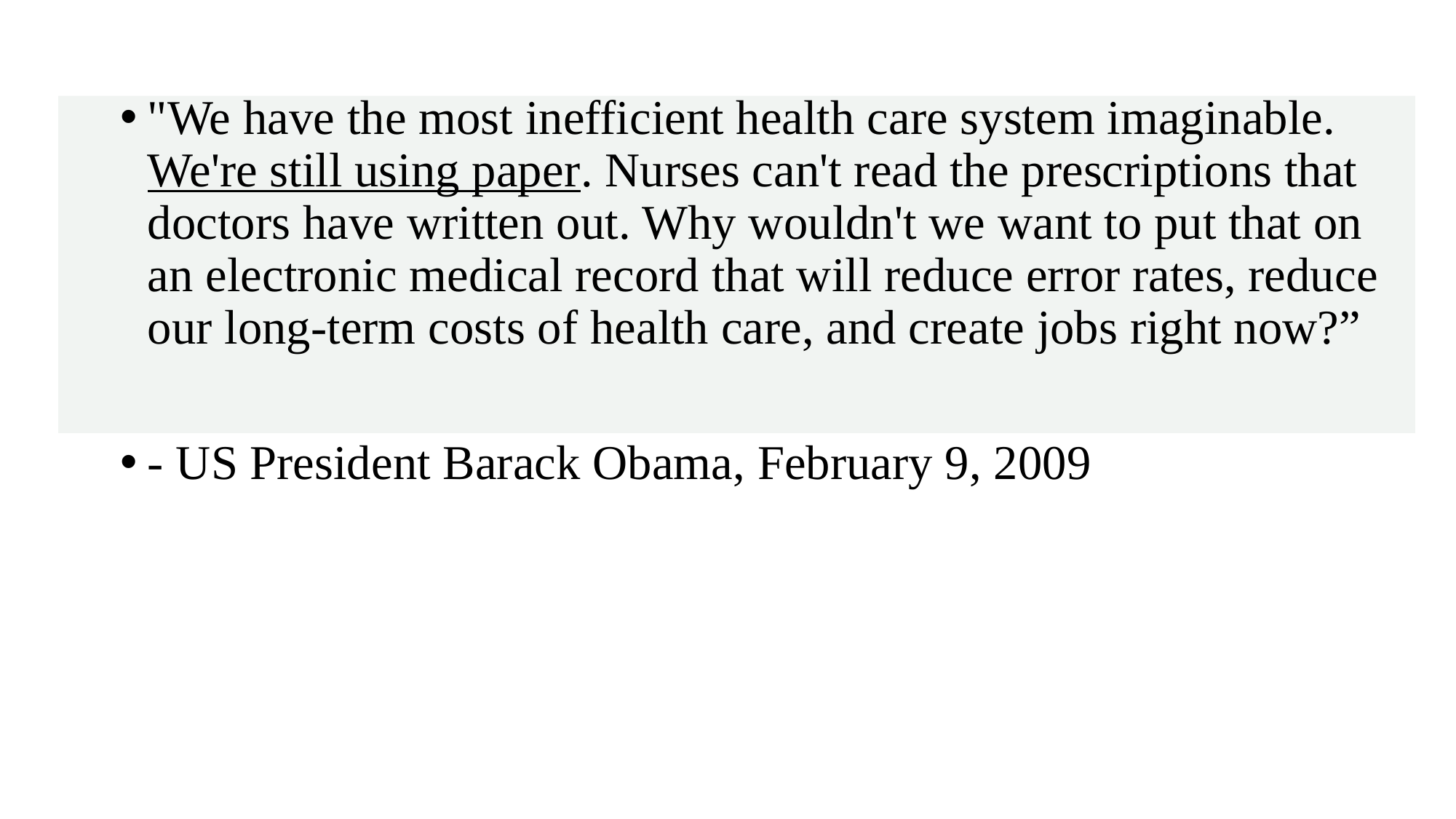

"We have the most inefficient health care system imaginable. We're still using paper. Nurses can't read the prescriptions that doctors have written out. Why wouldn't we want to put that on an electronic medical record that will reduce error rates, reduce our long-term costs of health care, and create jobs right now?”
- US President Barack Obama, February 9, 2009

## Slide 7
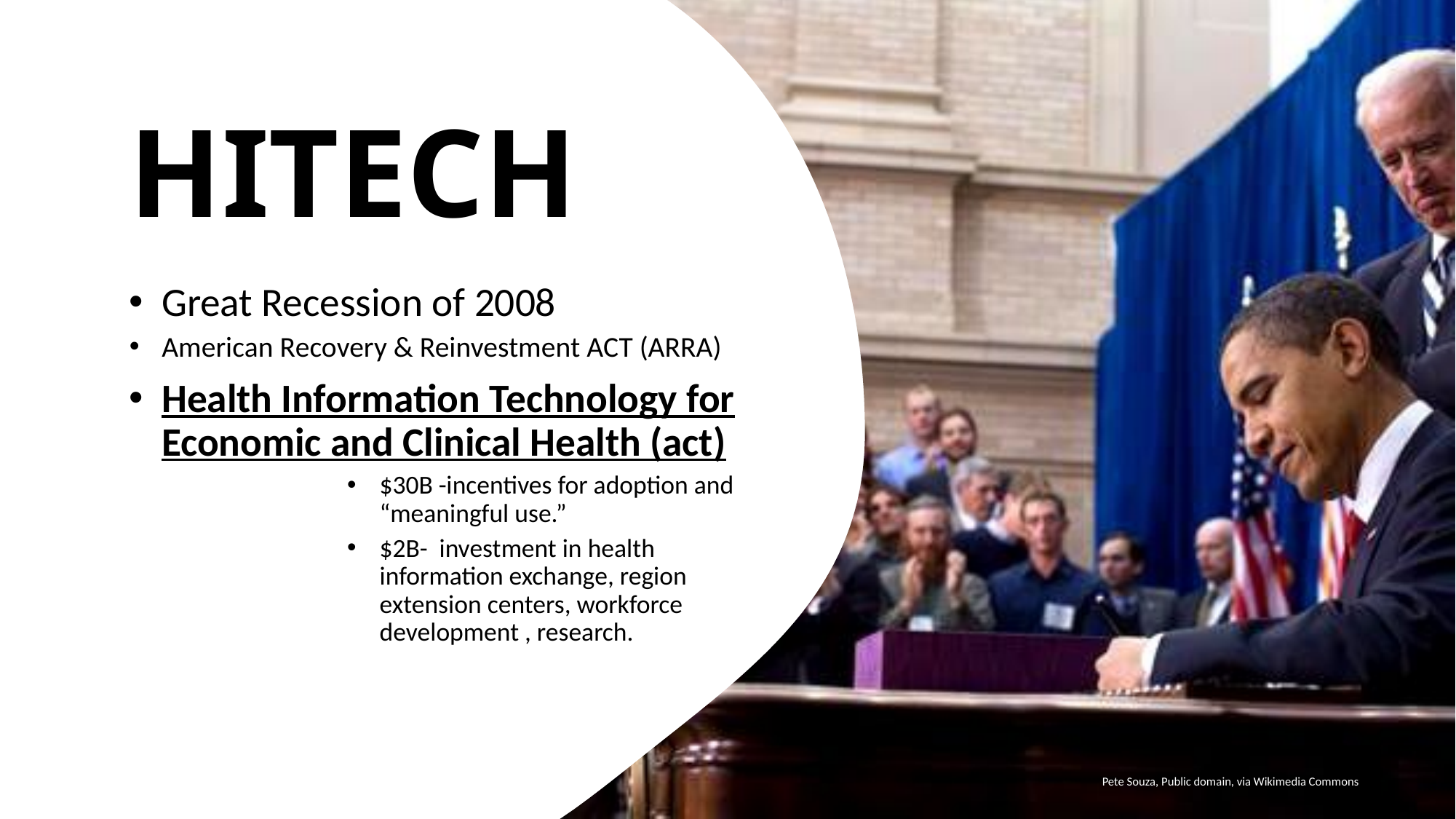

# HITECH
Great Recession of 2008
American Recovery & Reinvestment ACT (ARRA)
Health Information Technology for Economic and Clinical Health (act)
$30B -incentives for adoption and “meaningful use.”
$2B- investment in health information exchange, region extension centers, workforce development , research.
Pete Souza, Public domain, via Wikimedia Commons

## Slide 8
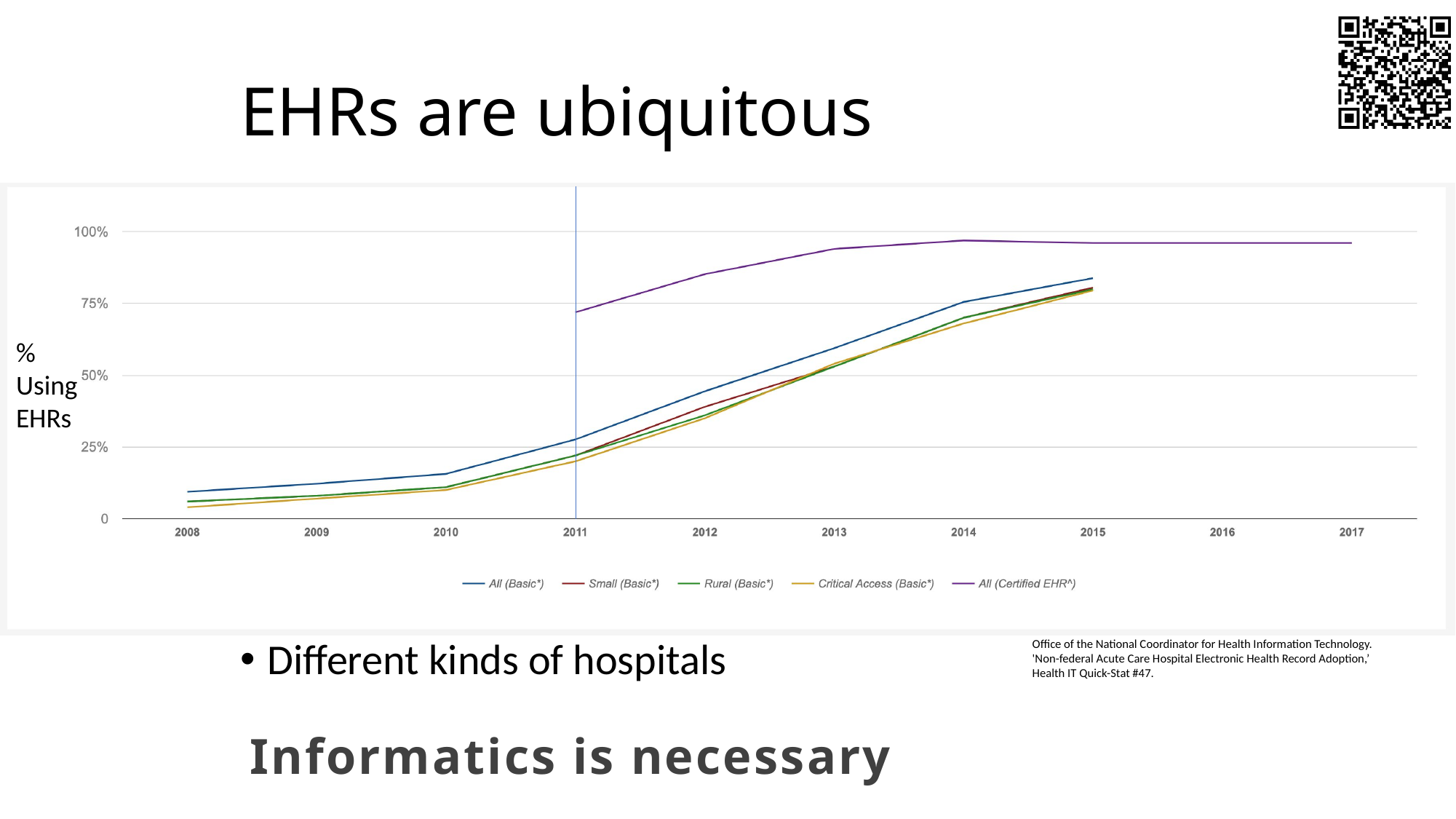

# EHRs are ubiquitous
%
Using EHRs
Office of the National Coordinator for Health Information Technology.
'Non-federal Acute Care Hospital Electronic Health Record Adoption,’
Health IT Quick-Stat #47.
Different kinds of hospitals
Informatics is necessary

## Slide 9
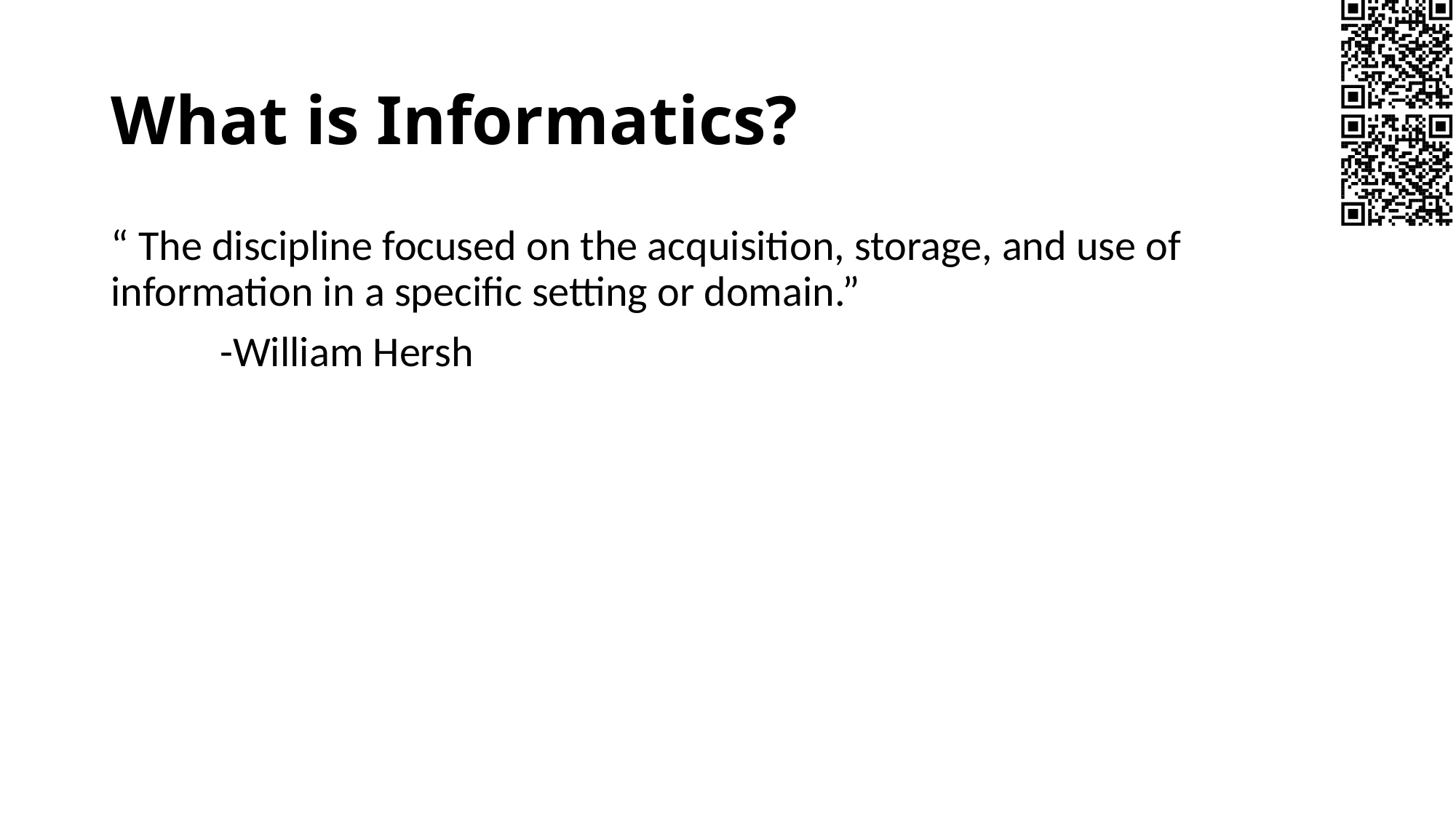

# What is Informatics?
“ The discipline focused on the acquisition, storage, and use of 	information in a specific setting or domain.”
	-William Hersh

## Slide 10
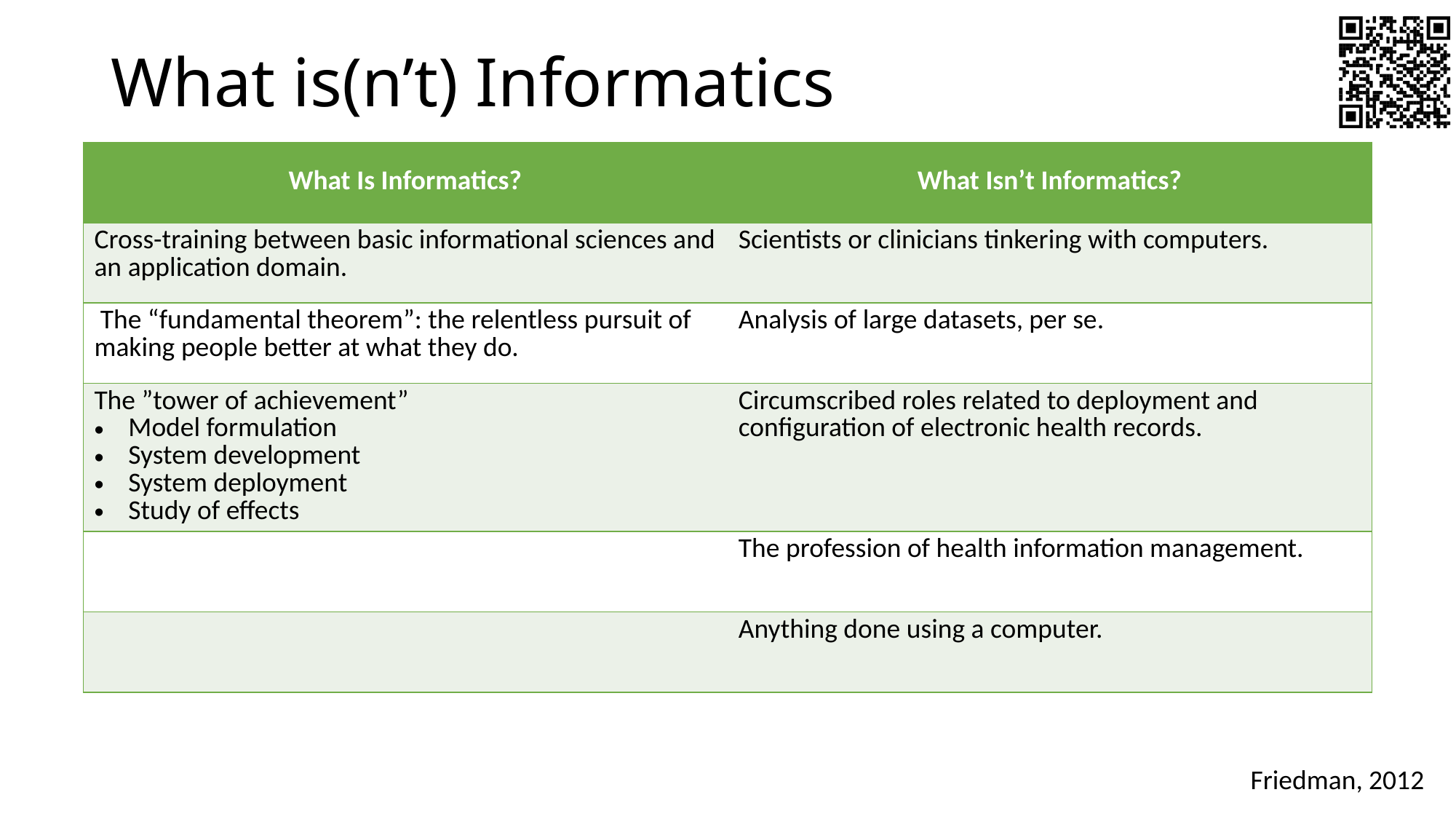

# What is(n’t) Informatics
| What Is Informatics? | What Isn’t Informatics? |
| --- | --- |
| Cross-training between basic informational sciences and an application domain. | Scientists or clinicians tinkering with computers. |
| The “fundamental theorem”: the relentless pursuit of making people better at what they do. | Analysis of large datasets, per se. |
| The ”tower of achievement” Model formulation System development System deployment Study of effects | Circumscribed roles related to deployment and configuration of electronic health records. |
| | The profession of health information management. |
| | Anything done using a computer. |
Friedman, 2012

## Slide 11
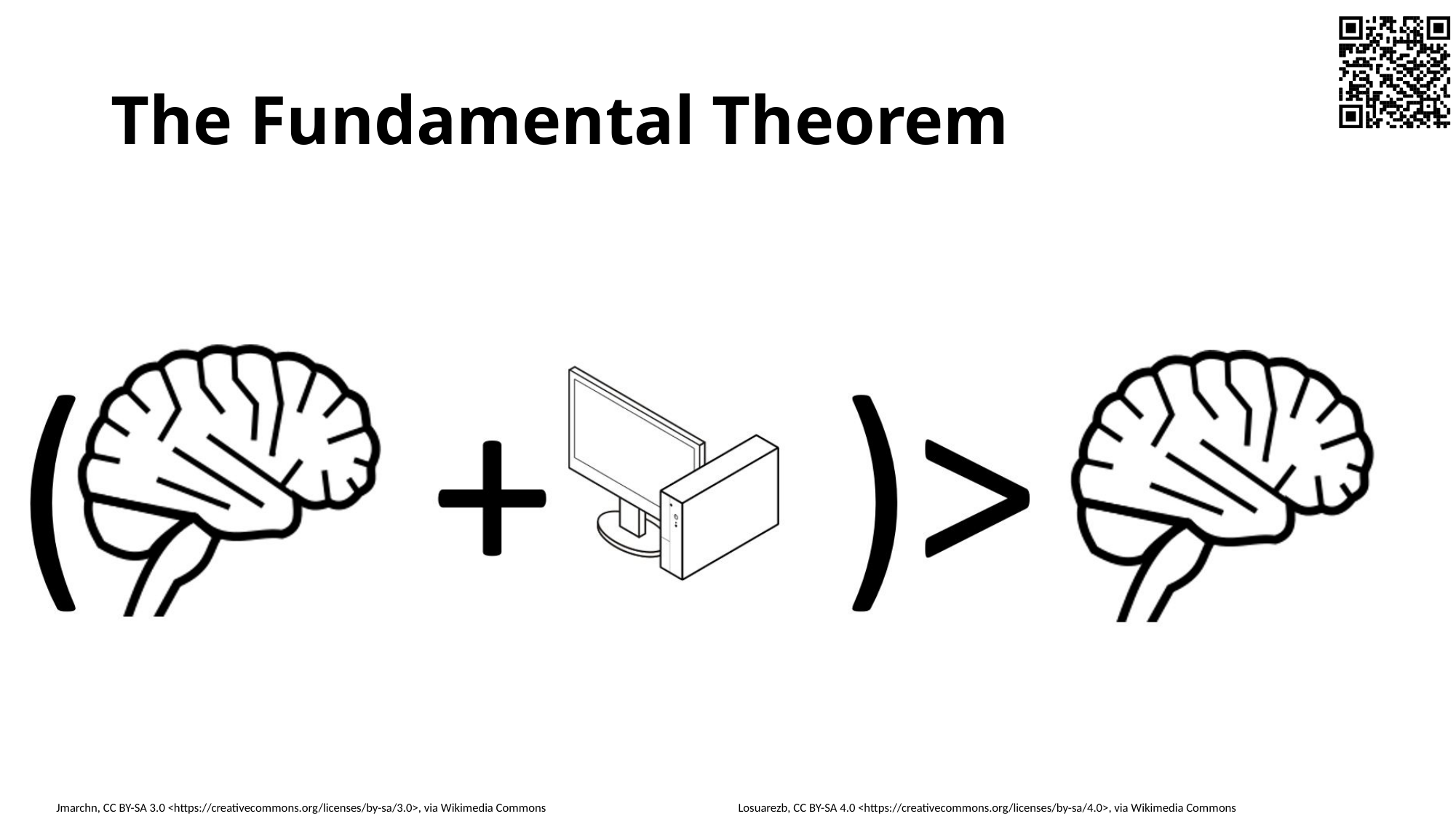

# The Fundamental Theorem
( + )>
Jmarchn, CC BY-SA 3.0 <https://creativecommons.org/licenses/by-sa/3.0>, via Wikimedia Commons
Losuarezb, CC BY-SA 4.0 <https://creativecommons.org/licenses/by-sa/4.0>, via Wikimedia Commons

## Slide 12
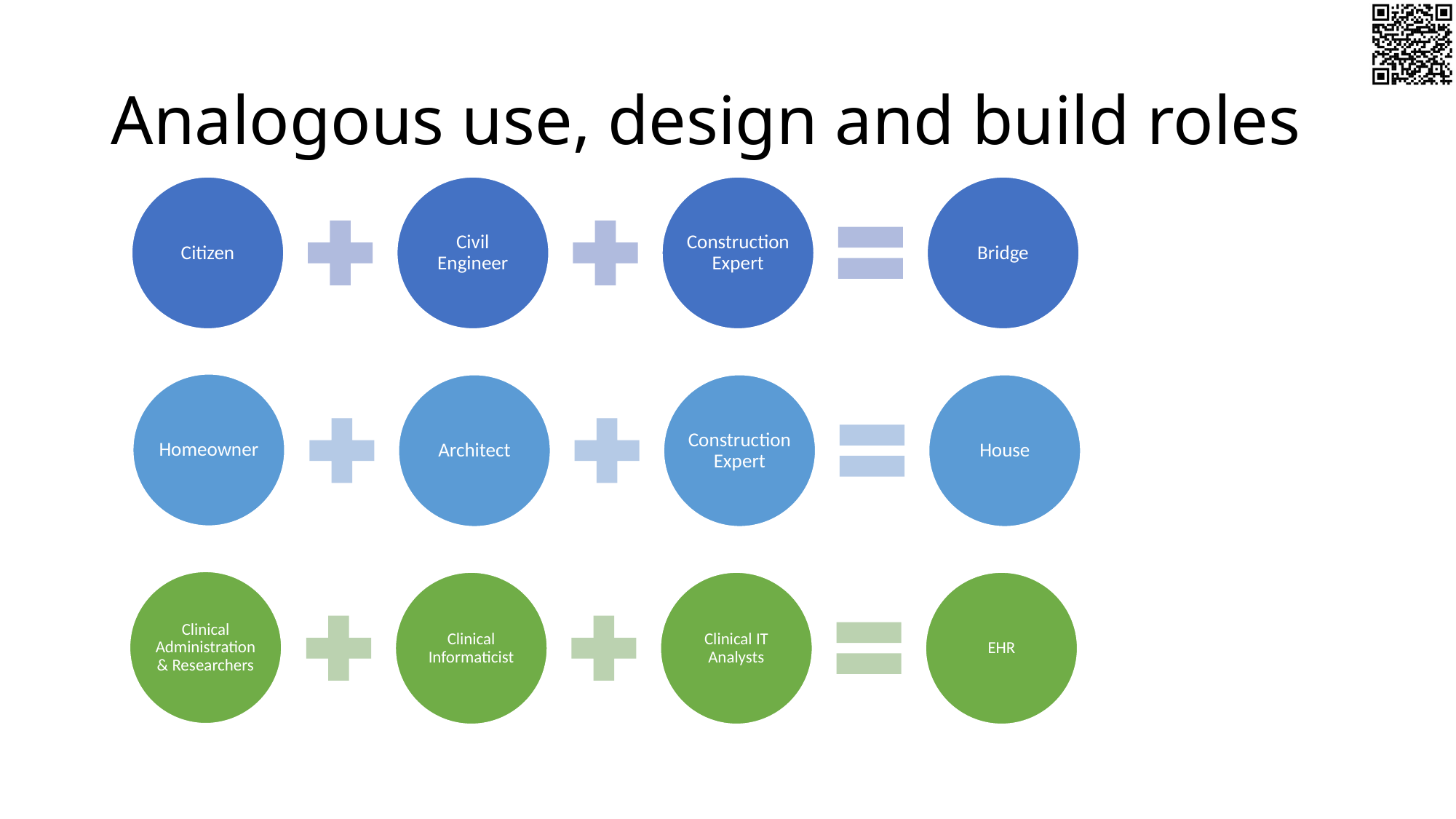

# Analogous use, design and build roles

## Slide 13
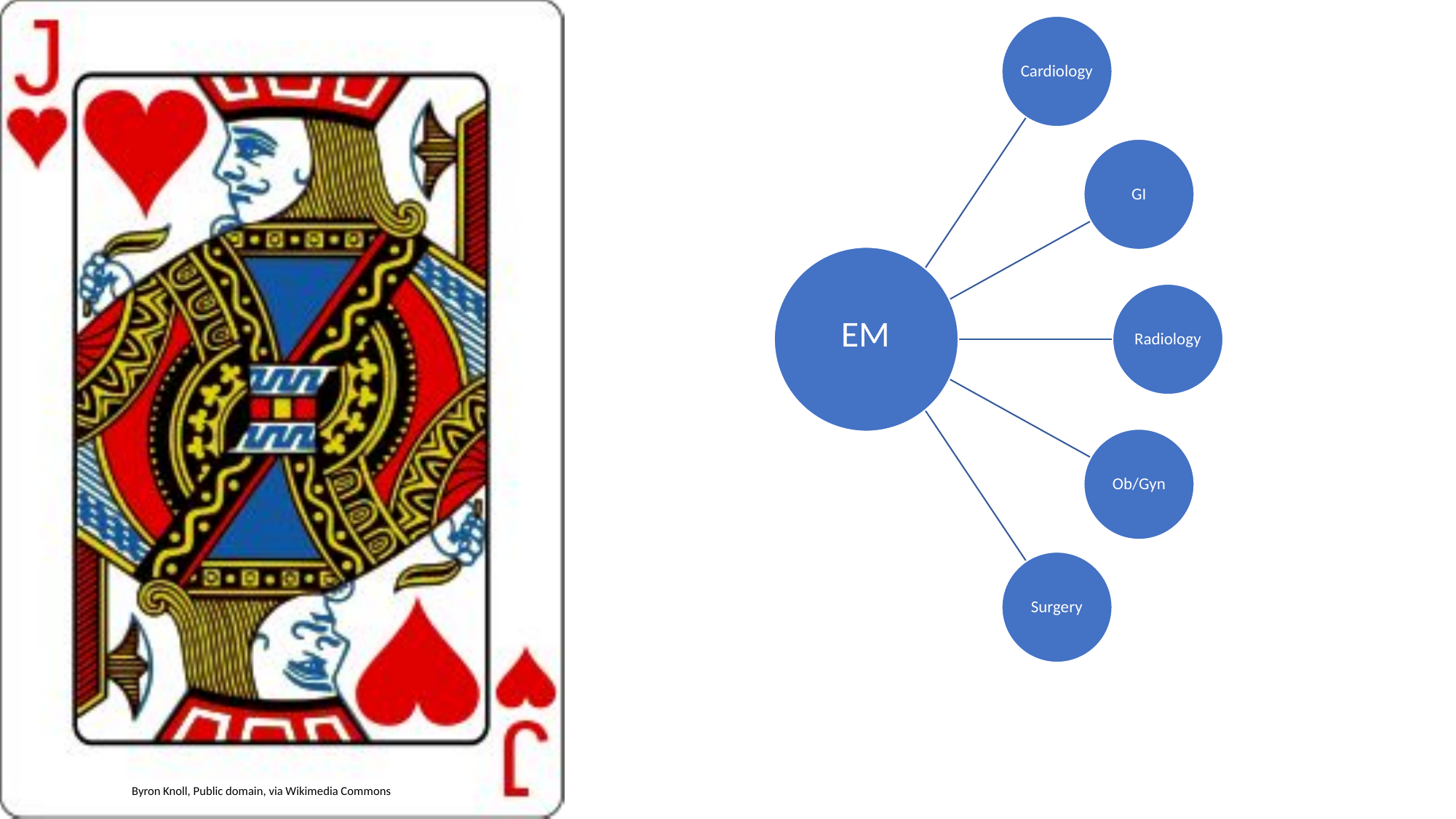

EM
Byron Knoll, Public domain, via Wikimedia Commons

## Slide 14
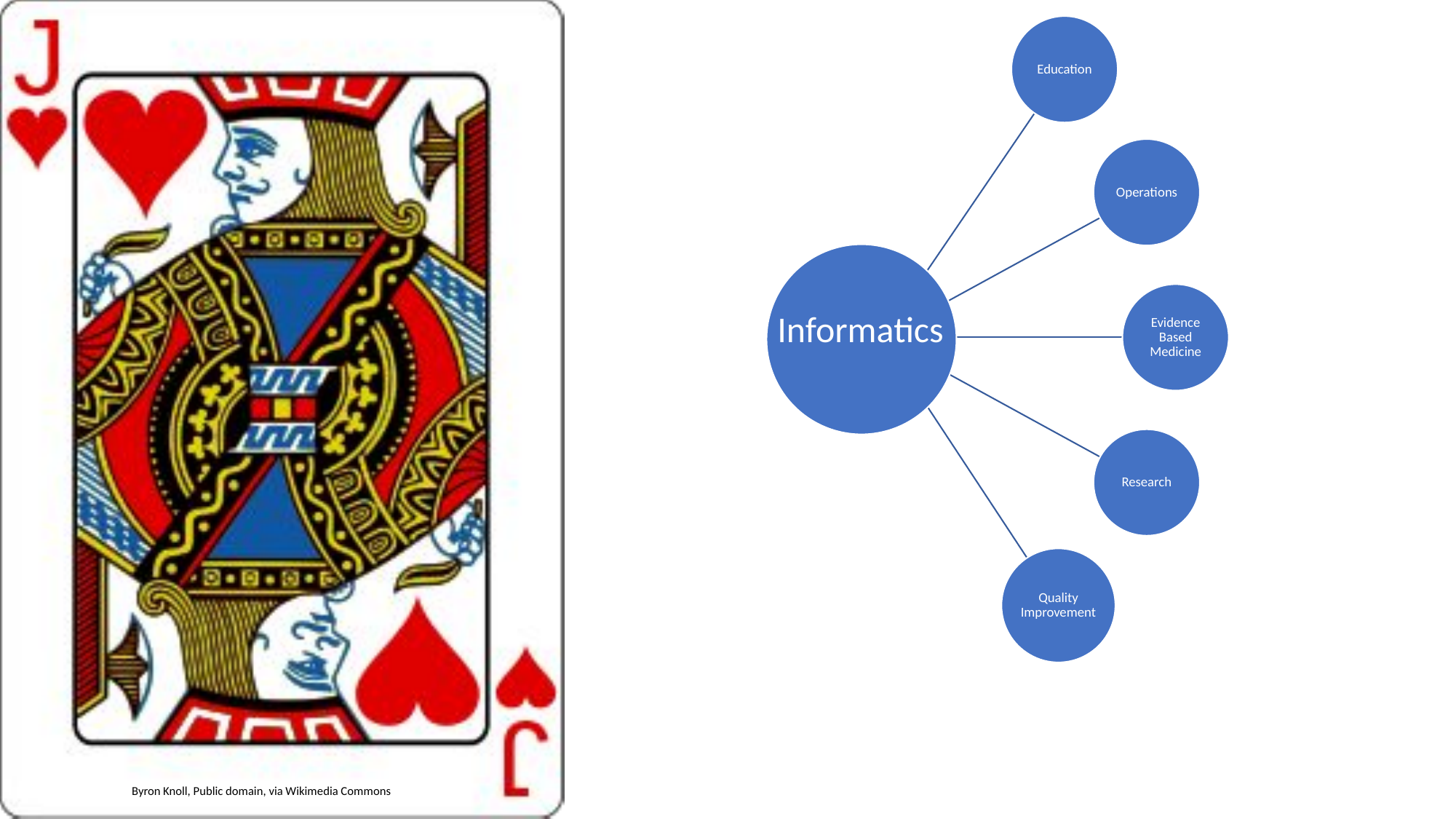

Informatics
Byron Knoll, Public domain, via Wikimedia Commons

## Slide 15
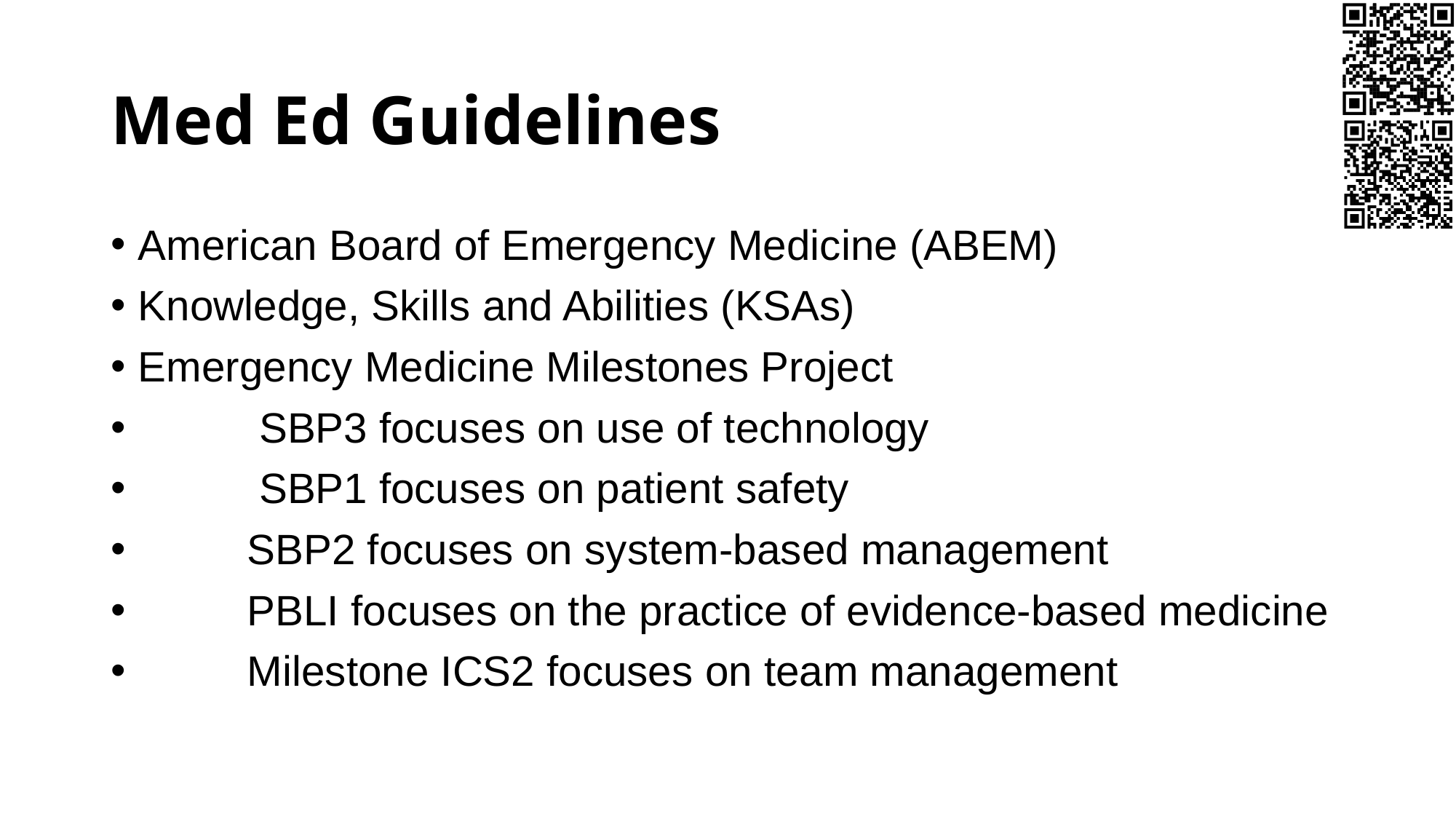

# Med Ed Guidelines
American Board of Emergency Medicine (ABEM)
Knowledge, Skills and Abilities (KSAs)
Emergency Medicine Milestones Project
	 SBP3 focuses on use of technology
	 SBP1 focuses on patient safety
	SBP2 focuses on system-based management
	PBLI focuses on the practice of evidence-based medicine
	Milestone ICS2 focuses on team management

## Slide 16
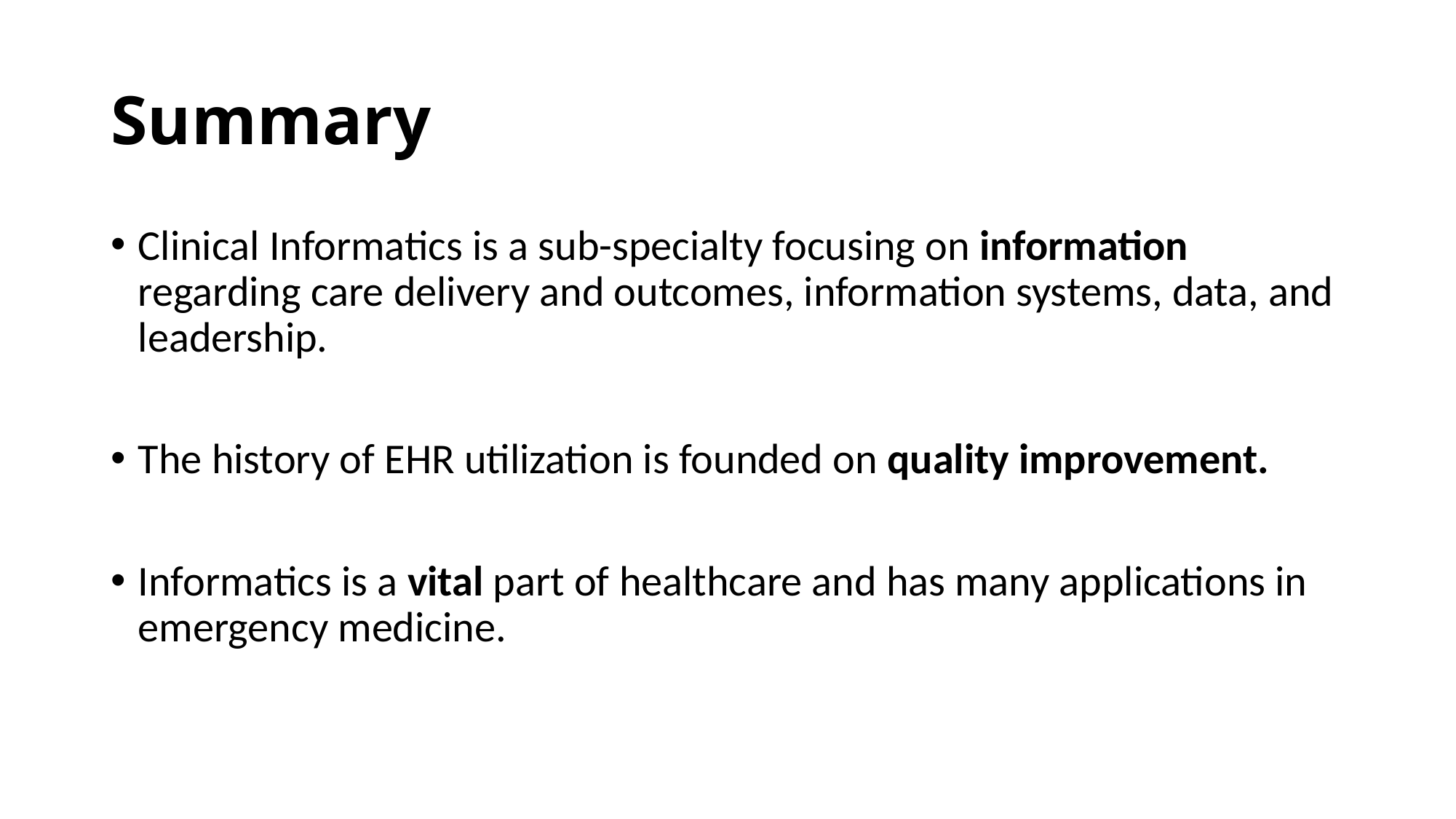

# Summary
Clinical Informatics is a sub-specialty focusing on information regarding care delivery and outcomes, information systems, data, and leadership.
The history of EHR utilization is founded on quality improvement.
Informatics is a vital part of healthcare and has many applications in emergency medicine.

## Slide 17
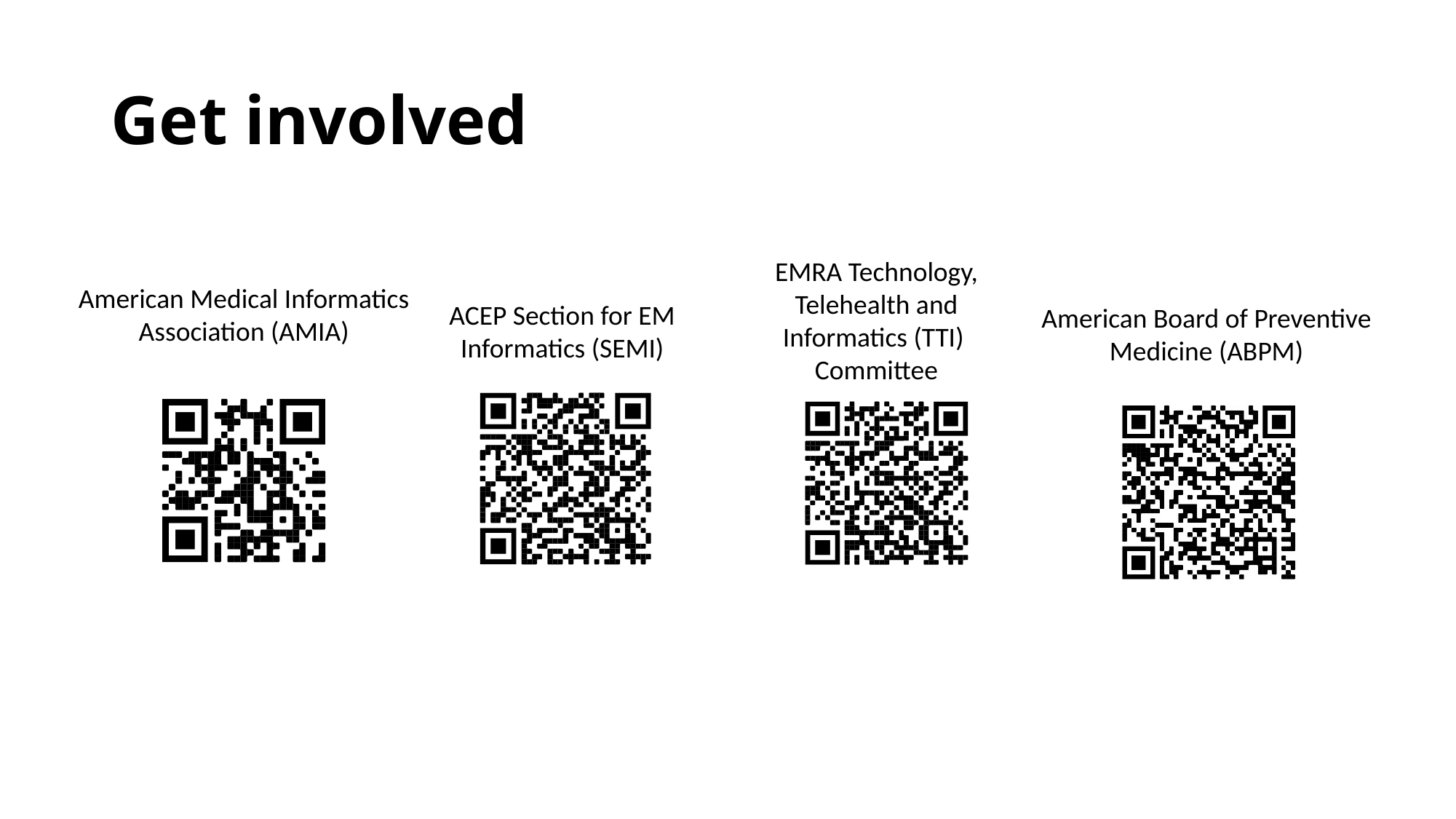

# Get involved
EMRA Technology, Telehealth and Informatics (TTI) Committee
American Medical Informatics Association (AMIA)
ACEP Section for EM Informatics (SEMI)
American Board of Preventive Medicine (ABPM)
