# Supplementary material for: A Model Curriculum for an Emergency Medicine Residency Rotation in Clinical Informatics
Source: J Educ Teach Emerg Med. 2022 Oct 15;7(4):C1–C50. doi: 10.21980/J82P9H (PMC10332664; doi:10.21980/J82P9H)
Supplement: Supplementary file 7 [file JETem-7-4-C1-AppendixE1c.docx]

Appendix E.1.b:

CI Fundamentals Instructor Material

1. Define Informatics in your own words, being sure to identify what it is and what it is not. Define clinical informatics and explain in detail how it differs from other fields traditionally associated with Health Information Technology.
   - 1. An appropriate definition should include the themes of acquisition, storage, and use of information. While there is a current association with information technology this is not a critical part of the definition itself.
     2. Informatics is an association between informational science and a domain. It is not doctors “tinkering” with computers, nor large data analysis (though this can be part of the development of informatics interventions).
     3. It is not health information management, nor solely implementation science.
2. Using your own words, generate an example of Friedman’s goal of informatics. Can you think of an example of how the rule can be applied in your clinical workflows?
   - 1. Friedman’s “fundamental theorem” is the concept that a human plus an information system will perform superiorly to a human alone. In the context of clinical informatics, we generally assume this to be that despite some of their pitfalls, humans working with an associated clinical information system will be superior to one working without an information system. An important note is that the human element is critical to the interaction.
3. Take an example from your clinical work and demonstrate how data can progress to information, then knowledge, then wisdom.
   - 1. An example might be how the data point for a blood test can be used to generate information about the result if there is a trend (ie, serial elevated blood glucose levels). This information could result in knowledge via the diagnoses of a patient’s condition (ie, a diagnosis of diabetes). The knowledge of the condition could contribute to wisdom and the appropriate interventions to manage the condition.
4. Describe the critical events leading up to the HITECH act and how it incentivized the use of electronic health records.
   - 1. The Great Recession of 2008 resulted in the passing of the American Recovery and Reinvestment Act (ARRA).
     2. A subset of ARRA was the Health Information Technology for Economic and Clinical Health (HITECH) act. The goal was to stimulate the economy and increase adoption of electronic health records (EHR) with improved quality and safety.
     3. Over $30B in incentives for the “meaningful use” of EHRs along with workforce development and research.
5. Define interoperability. Describe some of the past issues with it and how some policies are being developed to improve it. Describe an experience from your clinical work that demonstrated use of or a lack of interoperability.
   - 1. Interoperability implies the ability for systems to share information, but an important distinction in clinical informatics is not just the ability to share but also make use of the information.
     2. The HITECH act didn’t adequately promote interoperability due to a lack of development of standards, thus leading to a world of EHRs that didn’t talk to each other.
     3. The 21^st^ century Cures Act attempts to correct some of the issues with interoperability by prohibiting information blocking and setting a standard for information transfer (SMART on FHIR).
