# Supplementary material for: A Model Curriculum for an Emergency Medicine Residency Rotation in Clinical Informatics
Source: J Educ Teach Emerg Med. 2022 Oct 15;7(4):C1–C50. doi: 10.21980/J82P9H (PMC10332664; doi:10.21980/J82P9H)
Supplement: Supplementary file 8 [file JETem-7-4-C1-AppendixE1d.docx]

Appendix E.1.c:

CI Fundamentals Learner Material

1. Define Informatics in your own words, being sure to identify what it is and what it is not. Define clinical informatics and explain in detail how it differs from other fields traditionally associated with Health Information Technology.
2. Using your own words, generate an example of Friedman’s goal of informatics. Can you think of an example of how the rule can be applied in your clinical workflows?
3. Take an example from your clinical work and demonstrate how data can progress to information, then knowledge, then wisdom.
4. Describe the critical events leading up to the HITECH act and how it incentivized the use of electronic health records.
5. Define interoperability. Describe some of the past issues with it and how some policies are being developed to improve it. Describe an experience from your clinical work that demonstrated use of or a lack of interoperability.
