# Supplementary material for: A Model Curriculum for an Emergency Medicine Residency Rotation in Clinical Informatics
Source: J Educ Teach Emerg Med. 2022 Oct 15;7(4):C1–C50. doi: 10.21980/J82P9H (PMC10332664; doi:10.21980/J82P9H)
Supplement: Supplementary file 9 [file JETem-7-4-C1-AppendixE2a.docx]

Appendix E.2:

Small Group Discussion: Improving Care Delivery and Outcomes

**Pre-Session Preparation:**

This small group session is to be held at the end of the second week of the rotation. See the proposed calendar for details. Learners will have completed pre-readings during their asynchronous learning sessions. The instructor will be familiar with the presentation and will be familiar with the studies cited. Depending on the instructor’s baseline understanding, they may need to spend up to 6 hours becoming familiar with the published studies. The lecture will require about an hour to present depending on how much emphasis is placed on discussion, which we encourage. The CDS development worksheet will require another 45 minutes to an hour.

**Recommended Pre-Reading:**

Note: Items 1-6 are general background, and items 7-13 are for specific discussion during the PowerPoint lecture.

1. Ozkaynak M, Unertl KM, Johnson SA, Brixey JJ, Haque SN. Clinical Workflow Analysis, Process Redesign and Quality Improvement. In: Finnell JT, Dixon BE, eds. *Clinical Informatics Study Guide.* 2^nd^ ed. Springer; 2022:103-118.
2. Jenders RA. Clinical Decision Support. In: Hersh WR, ed. *Health Informatics: Practical* Guide. 8^th^ ed. Informatics Education. 2022:117-130.
3. Genes N. Digital Medicine for the Emergency Physician. ACEP Section for Emergency Medicine Informatics. Published 2016. Accessed April 13, 2022. At: https://www.acep.org/administration/quality/health-information-technology/hit-articles/digital-medicine-for-the-emergency-physician/
4. Hripcsak G, Clayton PD, Jenders RA, Cimino JJ, Johnson SB. Design of a clinical event monitor. *Comput Biomed Res*. 1996;29(3):194-221. doi:10.1006/cbmr.1996.0016
5. Han YY, Carcillo JA, Venkataraman ST, et al. Unexpected increased mortality after implementation of a commercially sold computerized physician order entry system [published correction appears in *Pediatrics*. 2006 Feb;117(2):594]. *Pediatrics*. 2005;116(6):1506-1512. doi:10.1542/peds.2005-1287
6. Farley HL, Baumlin KM, Hamedani AG, et al. Quality and safety implications of emergency department information systems. *Ann Emerg Med*. 2013;62(4):399-407. doi:10.1016/j.annemergmed.2013.05.019
7. Weingart SN, Simchowitz B, Padolsky H, et al. An empirical model to estimate the potential impact of medication safety alerts on patient safety, health care utilization, and cost in ambulatory care. *Arch Intern Med*. 2009;169(16):1465-1473. doi:10.1001/archinternmed.2009.252
8. Terrell KM, Perkins AJ, Hui SL, Callahan CM, Dexter PR, Miller DK. Computerized decision support for medication dosing in renal insufficiency: a randomized, controlled trial. *Ann Emerg Med*. 2010;56(6):623-629. doi:10.1016/j.annemergmed.2010.03.025
9. Powers EM, Shiffman RN, Melnick ER, Hickner A, Sharifi M. Efficacy and unintended consequences of hard-stop alerts in electronic health record systems: a systematic review. *J Am Med Inform Assoc*. 2018;25(11):1556-1566. doi:10.1093/jamia/ocy112
10. Levick D, Saldana L, Osheroff JA, eds. Healthcare Information and Management Systems Society, eds*. Improving Outcomes with Clinical Decision Support: An Implementer’s Guide*. 2nd ed. HIMSS; 2012.
11. Melnick ER, Genes NG, Chawla NK, Akerman M, Baumlin KM, Jagoda A. Knowledge translation of the American College of Emergency Physicians' clinical policy on syncope using computerized clinical decision support. *Int J Emerg Med*. 2010;3(2):97-104. Published 2010 Jun 1. doi:10.1007/s12245-010-0168-x
12. Bates DW, Kuperman GJ, Wang S, et al. Ten commandments for effective clinical decision support: making the practice of evidence-based medicine a reality. *J Am Med Inform Assoc*. 2003;10(6):523-530. doi:10.1197/jamia.M1370
13. Campbell RJ. The Five Rights of Clinical Decision Support: CDS Tools Helpful for Meeting Meaningful Use. Published online October 2013. Accessed April 1, 2022. At: https://library.ahima.org/doc?oid=300027#.YlXJ9t-M4uU
14. Strom BL, Schinnar R, Aberra F, et al. Unintended effects of a computerized physician order entry nearly hard-stop alert to prevent a drug interaction: a randomized controlled trial. *Arch Intern Med*. 2010;170(17):1578-1583. doi:10.1001/archinternmed.2010.324

**Objectives:**

By the end of this small group session, the learner will be able to:

1. Explain the relationship between physical workflow and the EHR workflow.
2. List the types of CDS.
3. Describe potential side effects of CDS.
4. Cite scientific evaluations of CDS.
5. Develop a short CDS proposal.

**Linked objectives and methods:**

This small group exercise begins with a slide presentation and discussion of CDS types and CDS evaluation, with guidance from the small group instructor. This is found in “Appendix E.2.a. Care Delivery Outcomes CDS PPT.” This presentation covers the first four objectives listed. As described above, the instructor will need to become familiar with the cited studies so that they can be discussed as a group. The slides take about 60 minutes to cover but are much more interesting if they include discussion about how their current EHR setup and prior experiences correspond to the study outcomes.

The fifth objective is "Develop a short CDS proposal.” This requires dividing the group into small teams of 2-5 individuals. Smaller groups are usually better so that everyone has a chance to contribute. Each learner is given a copy of “Appendix E.2.b. Care Delivery Outcomes CDS Development Form.” They are asked to consider a way that EHR might support their workflow, correct an error, or somehow make it easier to do the right thing. In their group they should discuss how it might be implemented and answer the questions on the sheet. After about 20 minutes of discussion in small groups, they should present their CDS suggestion to the larger group and discuss their design rationale.
