# Supplementary material for: A Model Curriculum for an Emergency Medicine Residency Rotation in Clinical Informatics
Source: J Educ Teach Emerg Med. 2022 Oct 15;7(4):C1–C50. doi: 10.21980/J82P9H (PMC10332664; doi:10.21980/J82P9H)
Supplement: Supplementary file 10 — Please see associated PowerPoint file [file JETem-7-4-C1-AppendixE2b.pptx]

## Slide 1
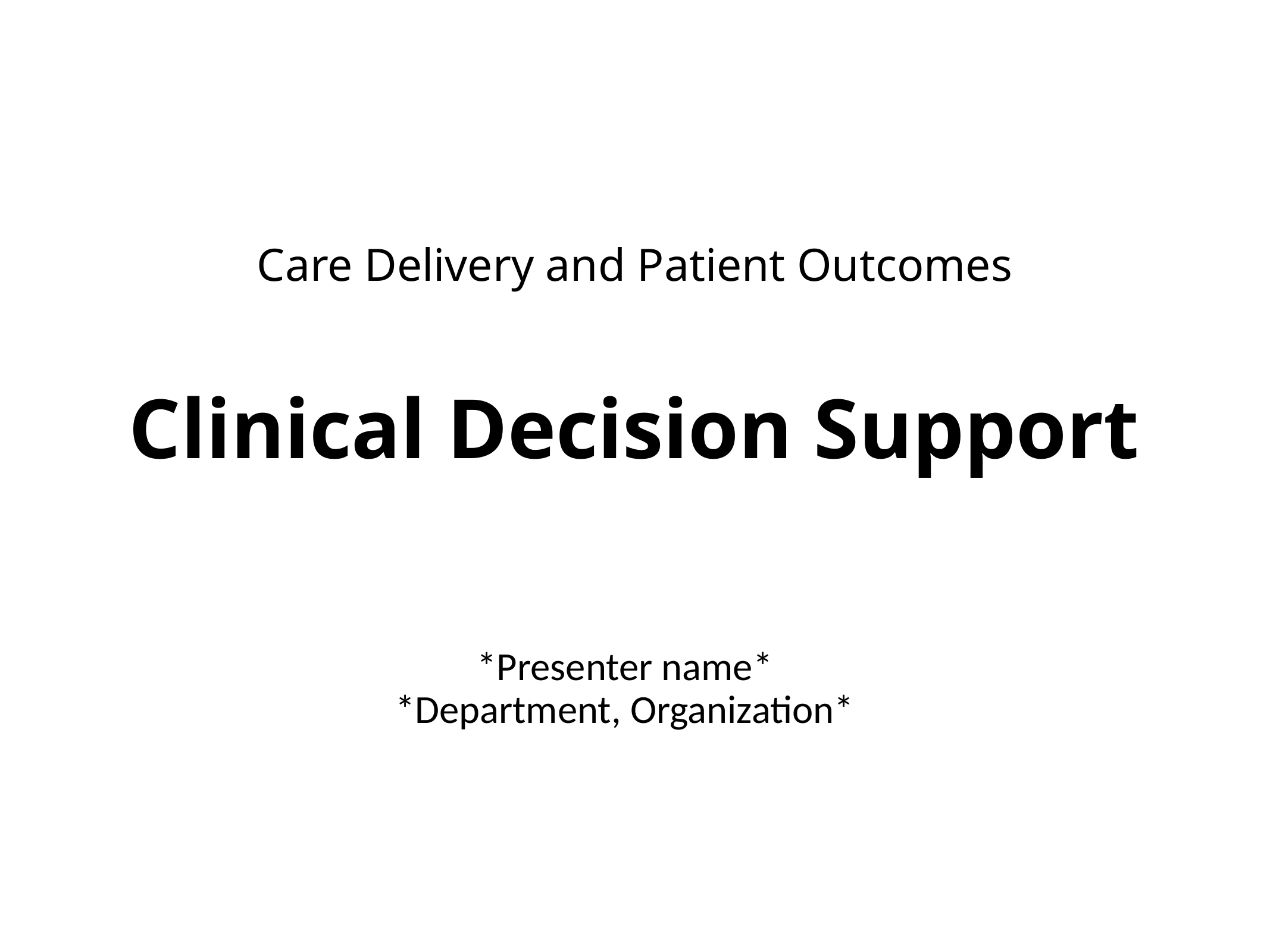

# Care Delivery and Patient Outcomes Clinical Decision Support
*Presenter name*
*Department, Organization*

## Slide 2
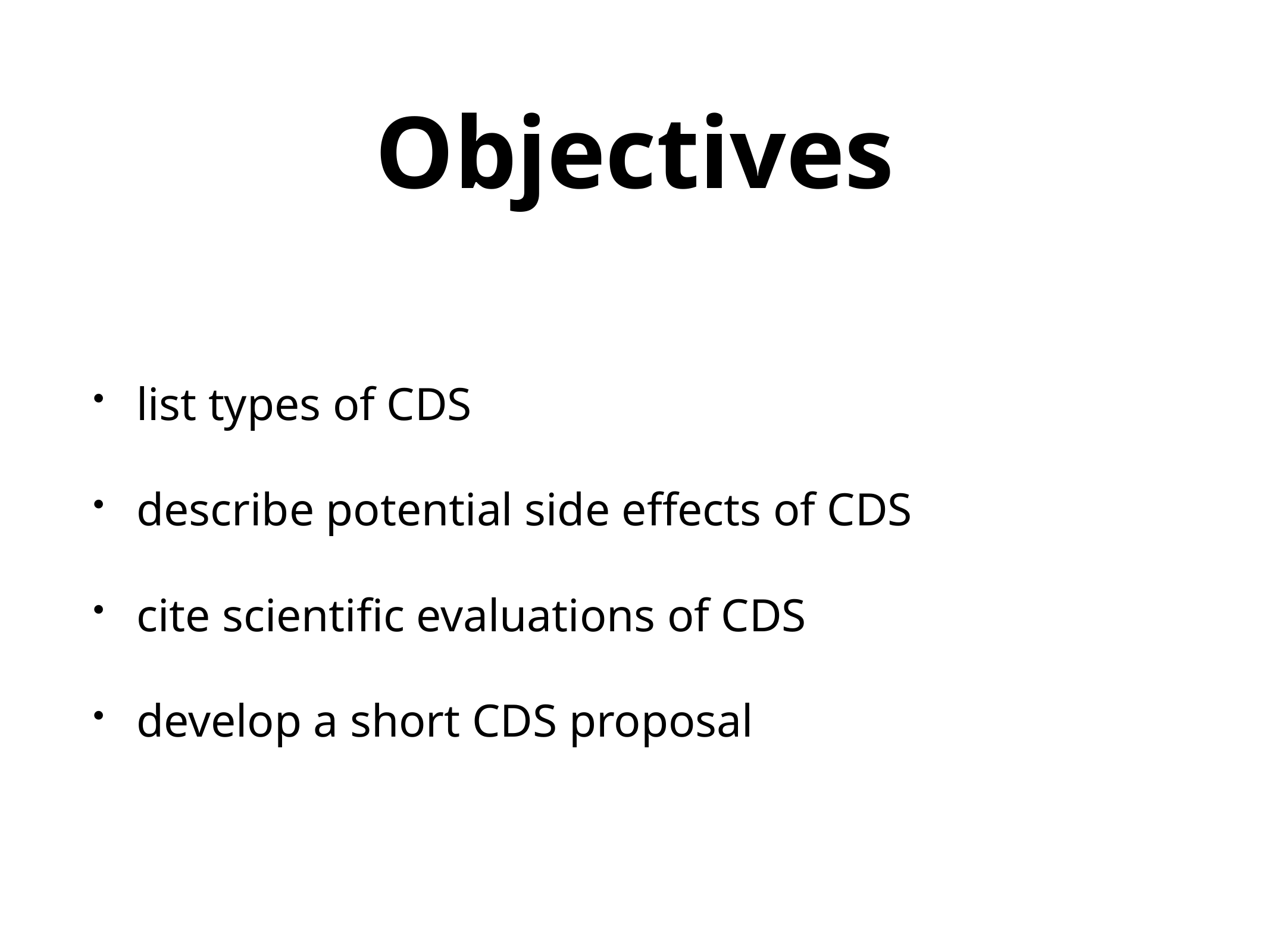

# Objectives
list types of CDS
describe potential side effects of CDS
cite scientific evaluations of CDS
develop a short CDS proposal

## Slide 3
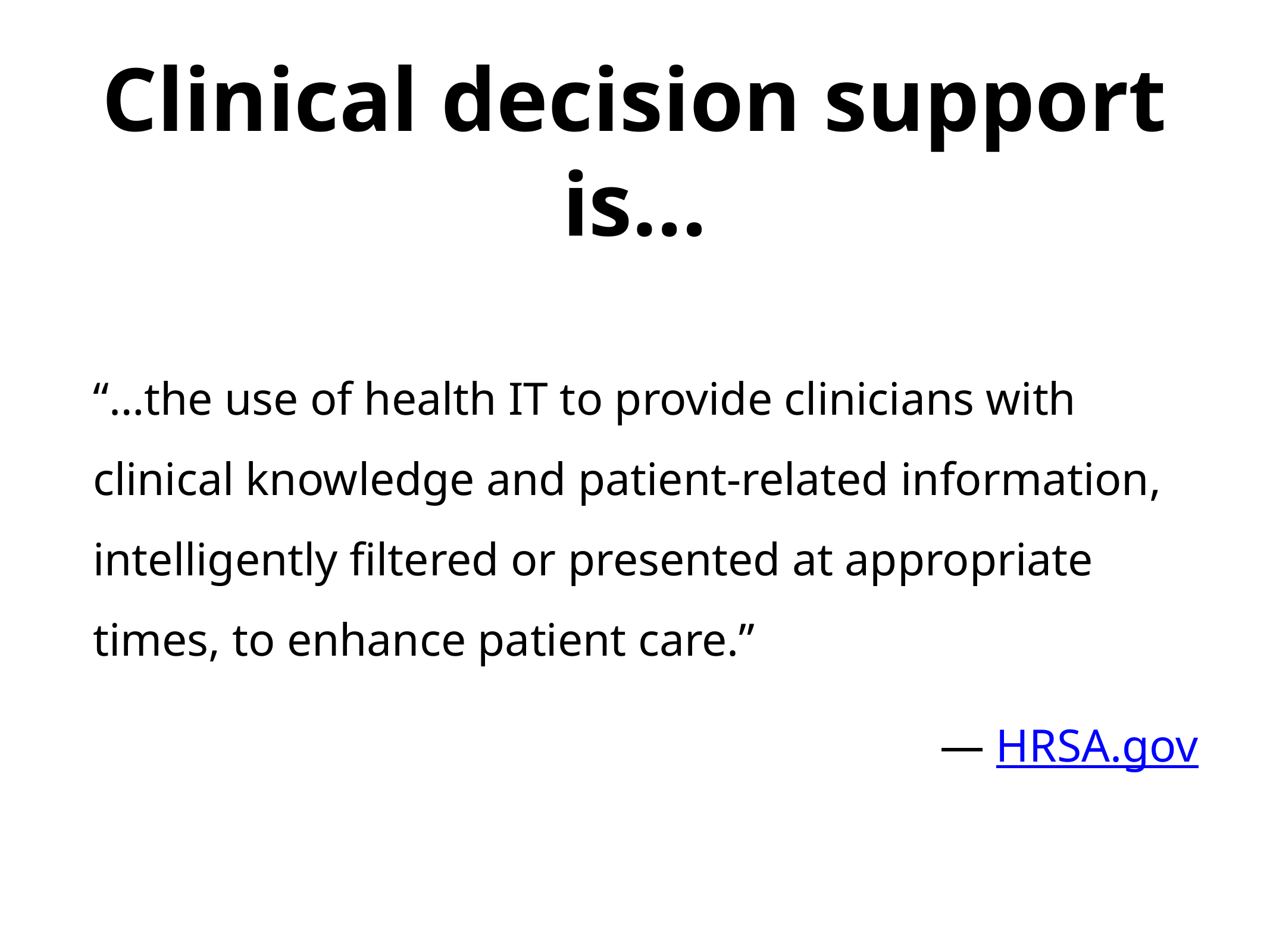

# Clinical decision support is…
“…the use of health IT to provide clinicians with clinical knowledge and patient-related information, intelligently filtered or presented at appropriate times, to enhance patient care.”
— HRSA.gov

## Slide 4
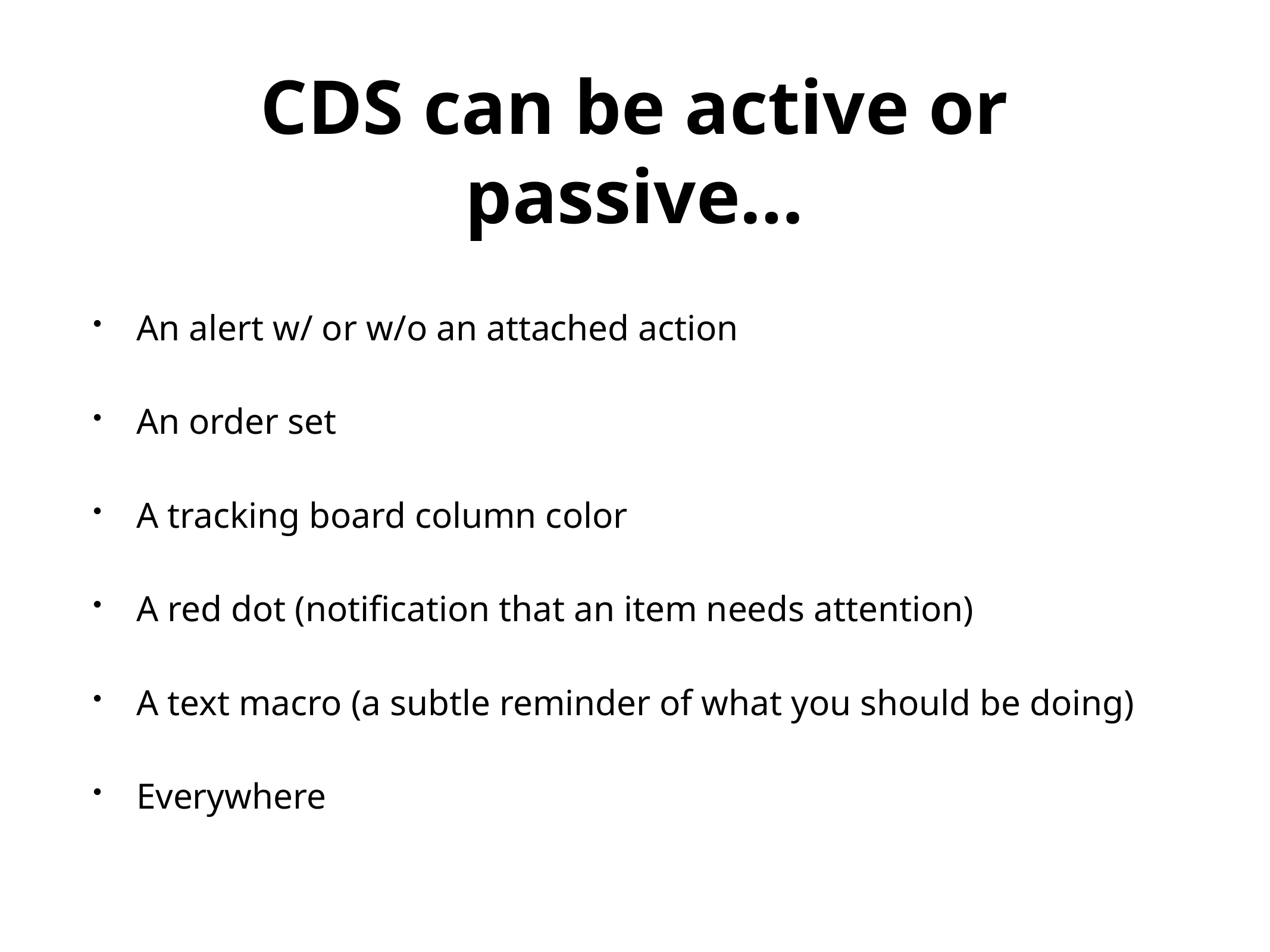

# CDS can be active or passive…
An alert w/ or w/o an attached action
An order set
A tracking board column color
A red dot (notification that an item needs attention)
A text macro (a subtle reminder of what you should be doing)
Everywhere

## Slide 5
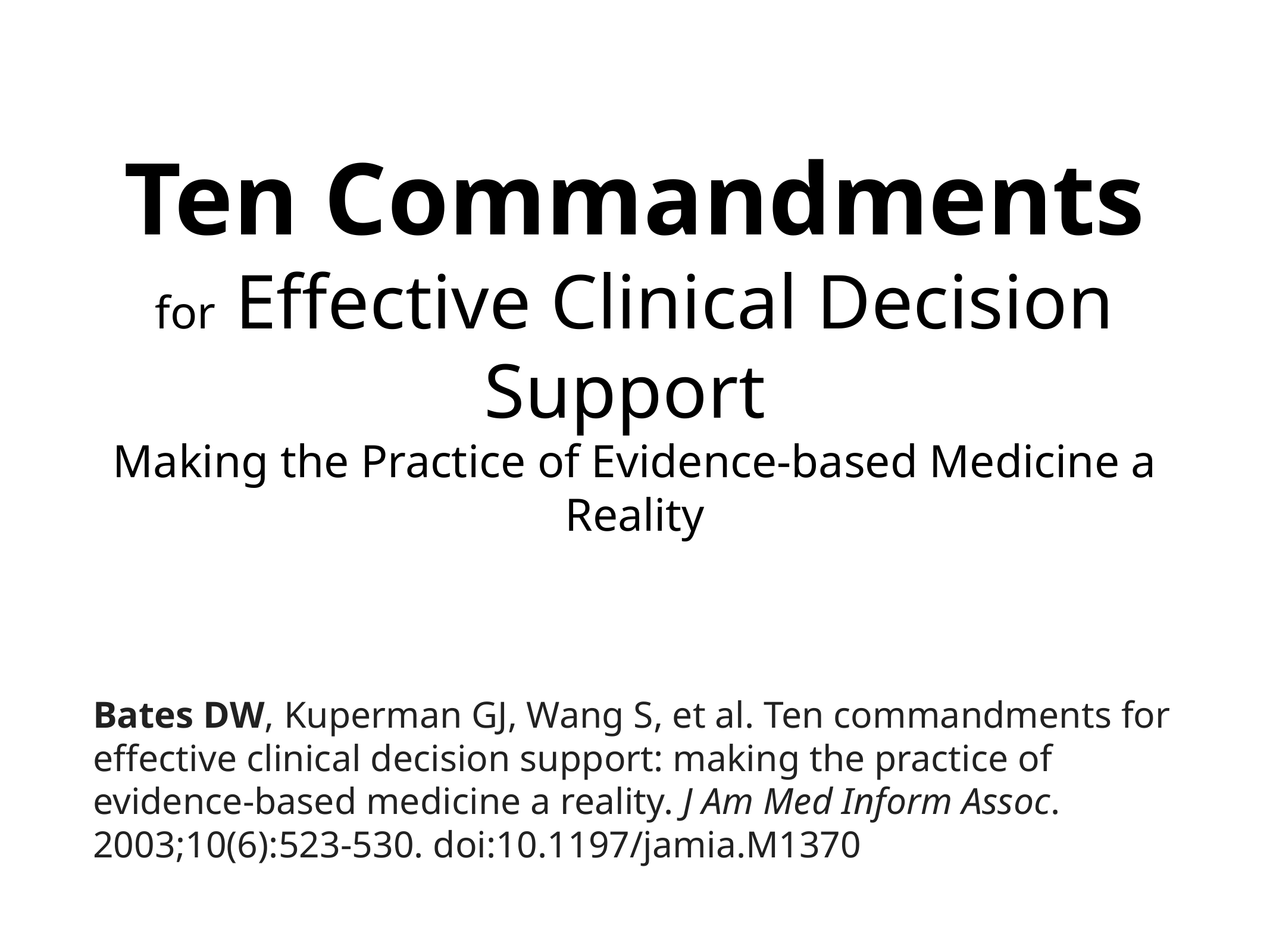

# Ten Commandments for Effective Clinical Decision Support Making the Practice of Evidence-based Medicine a Reality
Bates DW, Kuperman GJ, Wang S, et al. Ten commandments for effective clinical decision support: making the practice of evidence-based medicine a reality. J Am Med Inform Assoc. 2003;10(6):523-530. doi:10.1197/jamia.M1370

## Slide 6
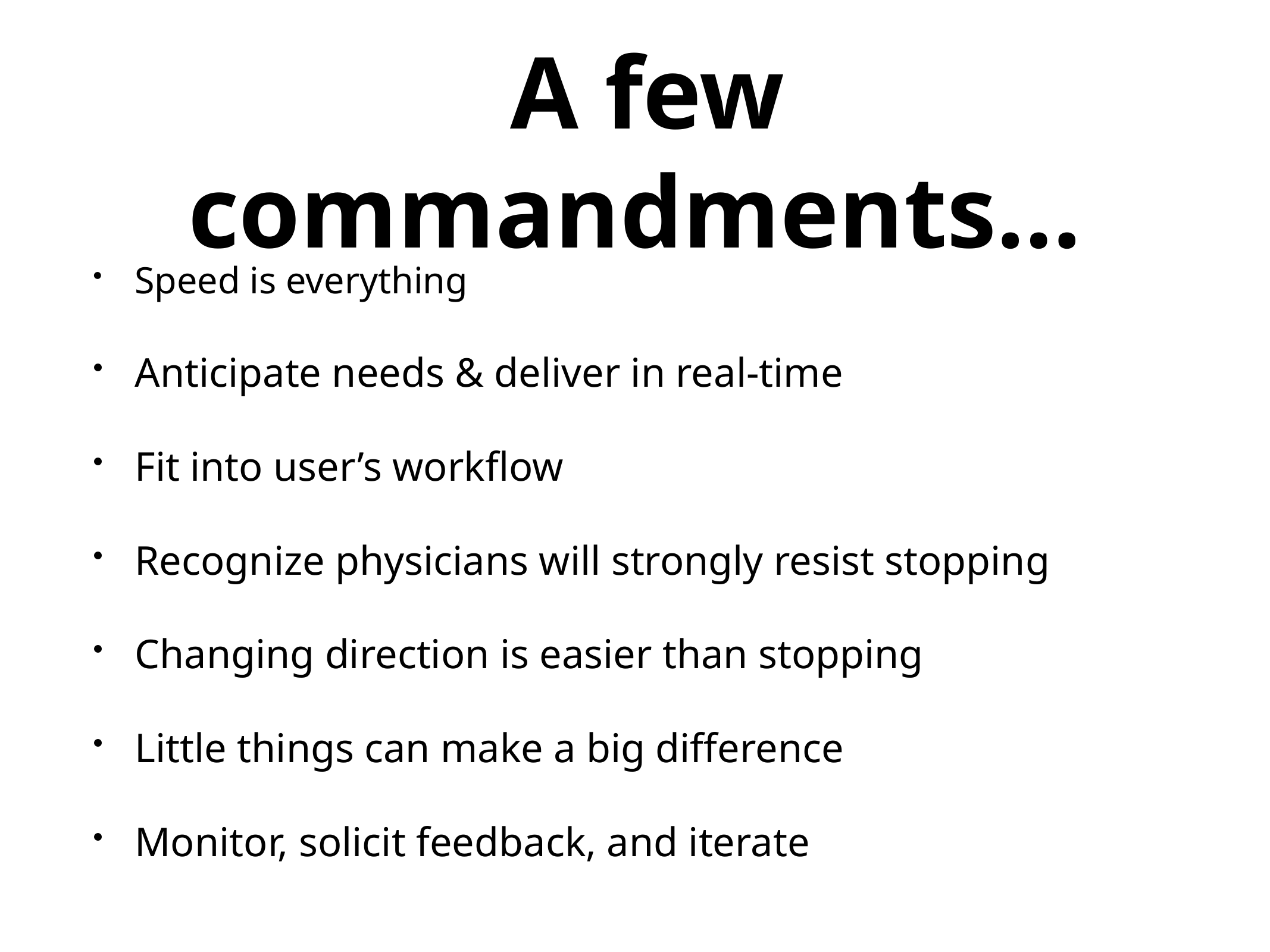

# A few commandments…
Speed is everything
Anticipate needs & deliver in real-time
Fit into user’s workflow
Recognize physicians will strongly resist stopping
Changing direction is easier than stopping
Little things can make a big difference
Monitor, solicit feedback, and iterate

## Slide 7
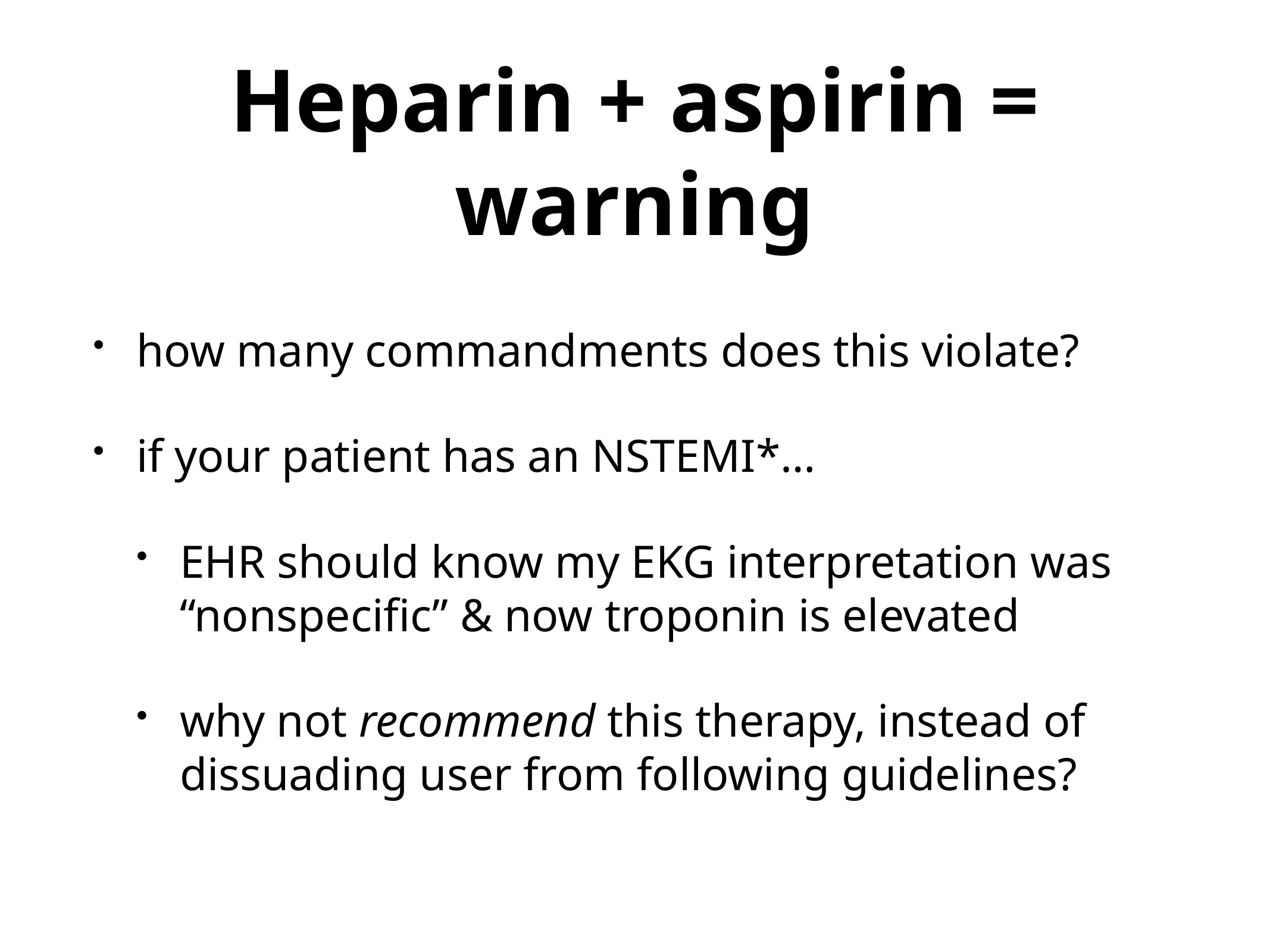

# Heparin + aspirin = warning
how many commandments does this violate?
if your patient has an NSTEMI*…
EHR should know my EKG interpretation was “nonspecific” & now troponin is elevated
why not recommend this therapy, instead of dissuading user from following guidelines?

## Slide 8
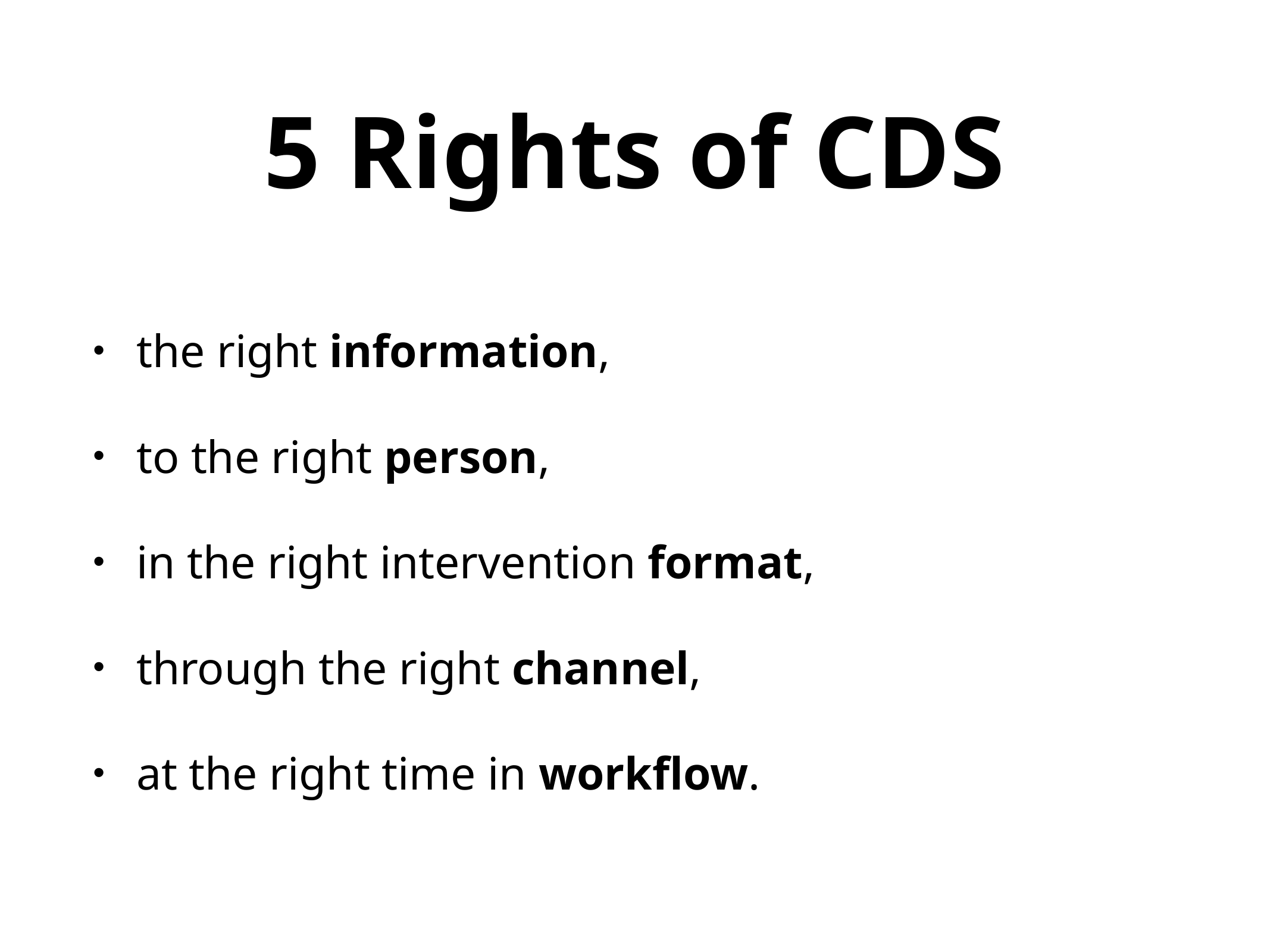

# 5 Rights of CDS
the right information,
to the right person,
in the right intervention format,
through the right channel,
at the right time in workflow.

## Slide 9
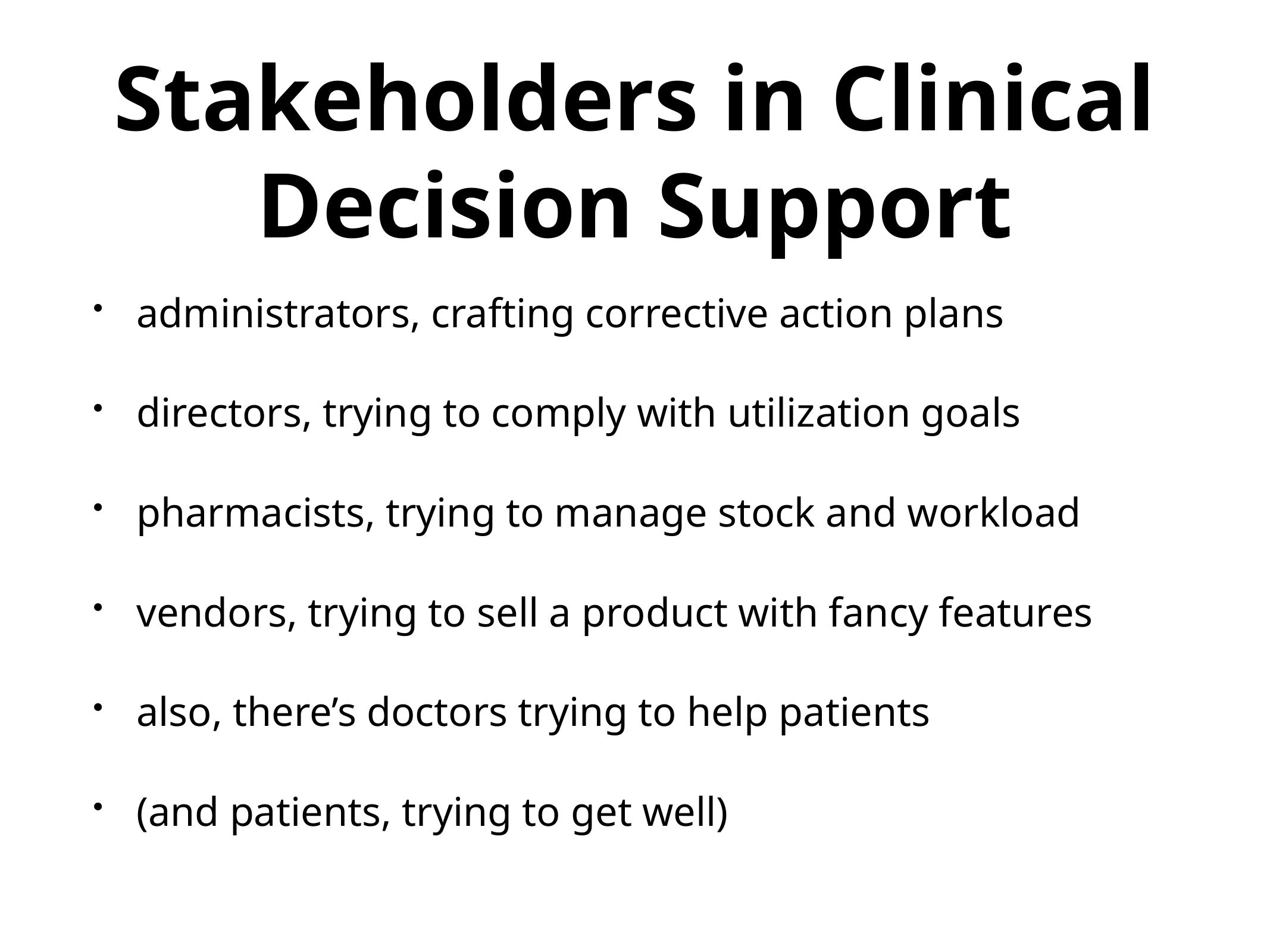

# Stakeholders in Clinical Decision Support
administrators, crafting corrective action plans
directors, trying to comply with utilization goals
pharmacists, trying to manage stock and workload
vendors, trying to sell a product with fancy features
also, there’s doctors trying to help patients
(and patients, trying to get well)

## Slide 10
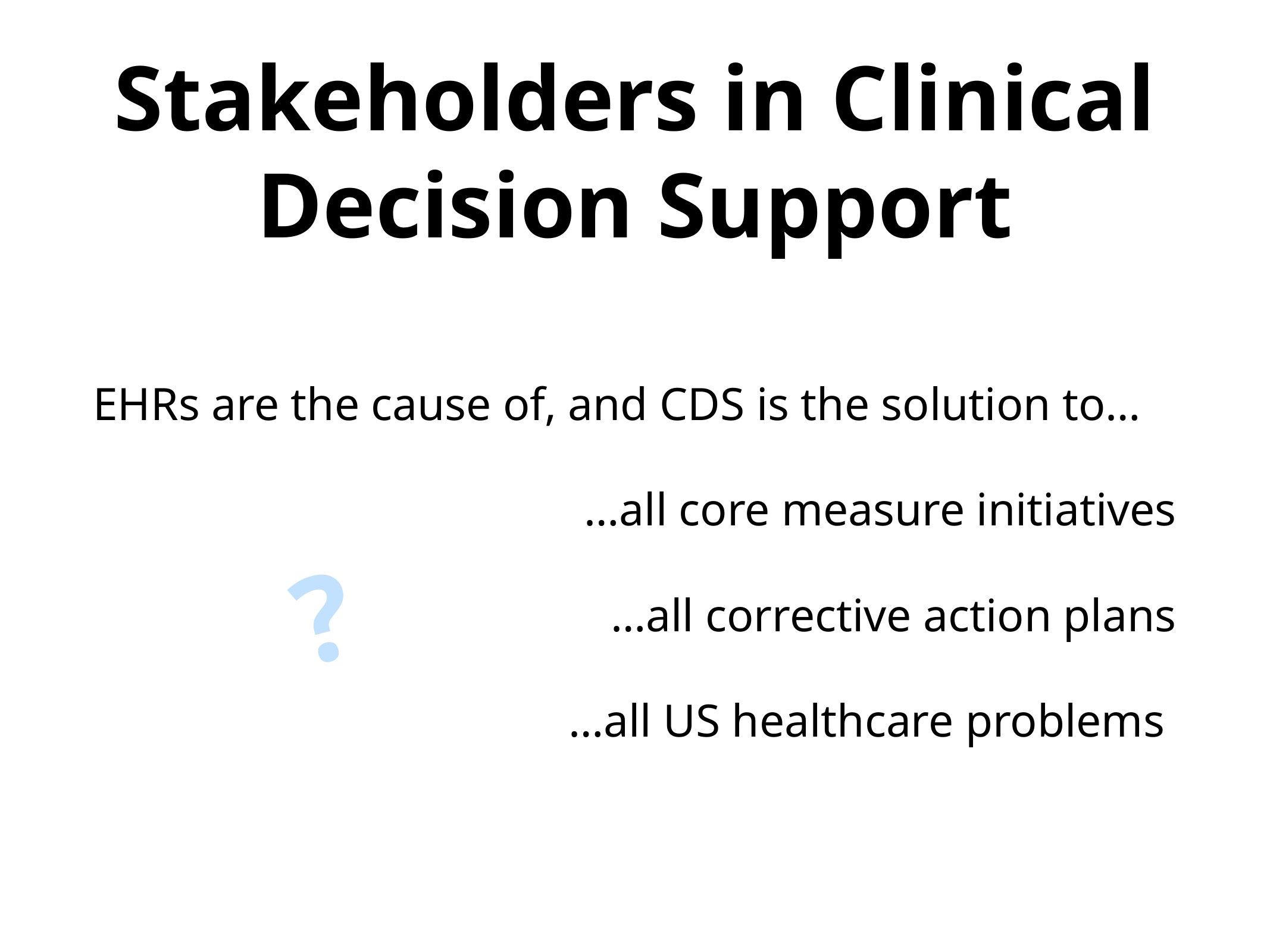

# Stakeholders in Clinical Decision Support
EHRs are the cause of, and CDS is the solution to…
…all core measure initiatives
…all corrective action plans
…all US healthcare problems
?

## Slide 11
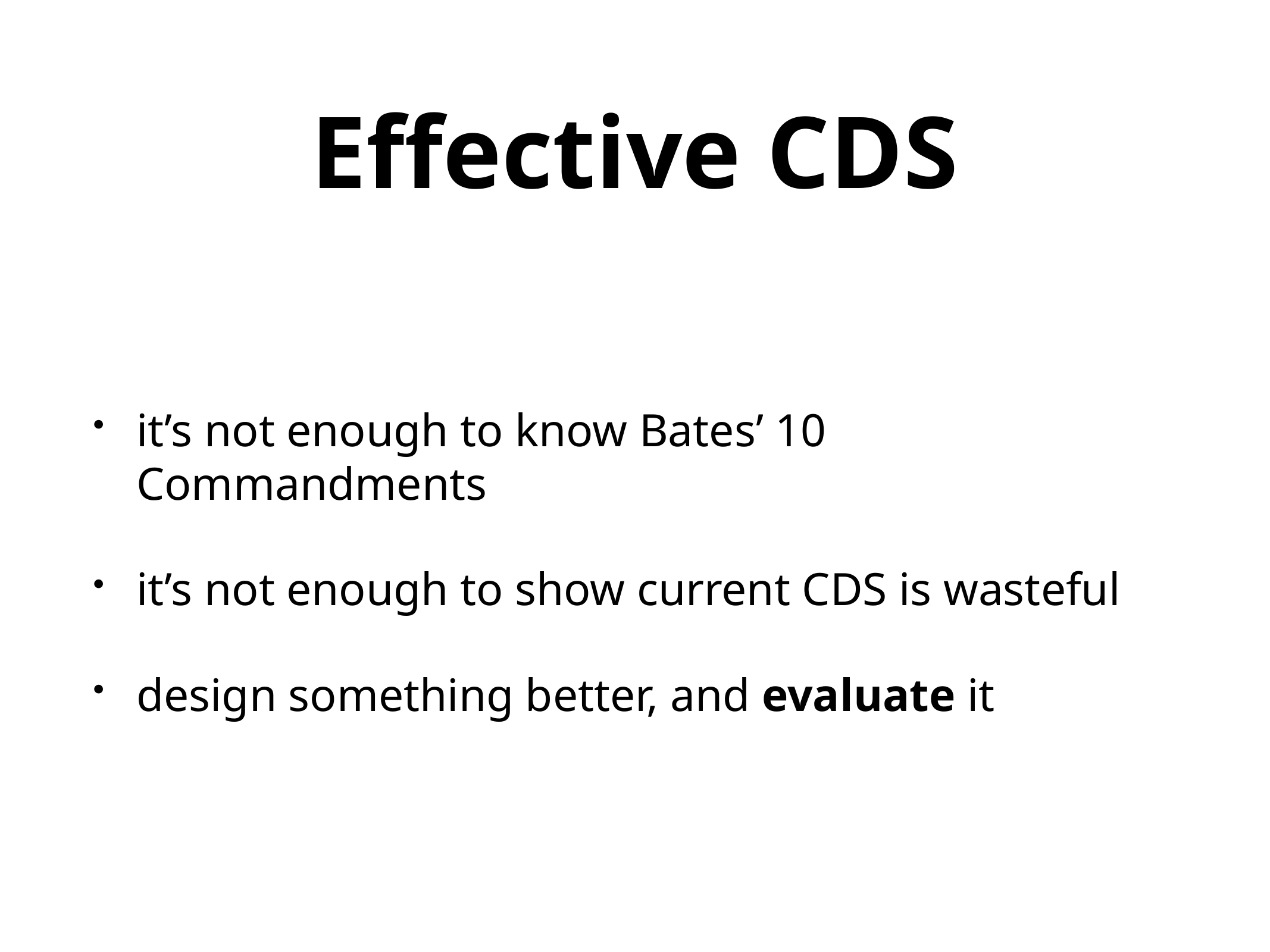

# Effective CDS
it’s not enough to know Bates’ 10 Commandments
it’s not enough to show current CDS is wasteful
design something better, and evaluate it

## Slide 12
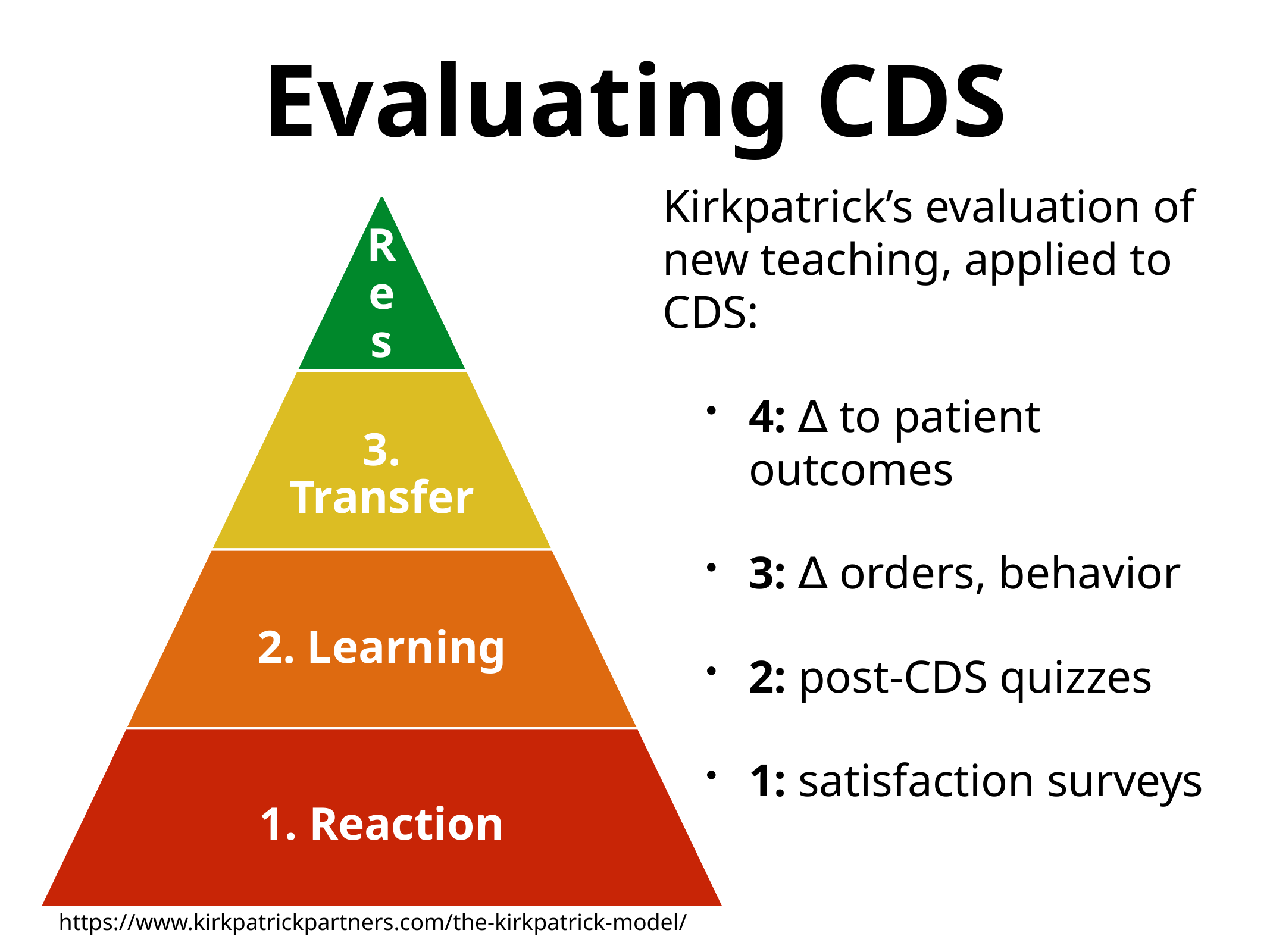

# Evaluating CDS
Kirkpatrick’s evaluation of new teaching, applied to CDS:
4: ∆ to patient outcomes
3: ∆ orders, behavior
2: post-CDS quizzes
1: satisfaction surveys
https://www.kirkpatrickpartners.com/the-kirkpatrick-model/

## Slide 13
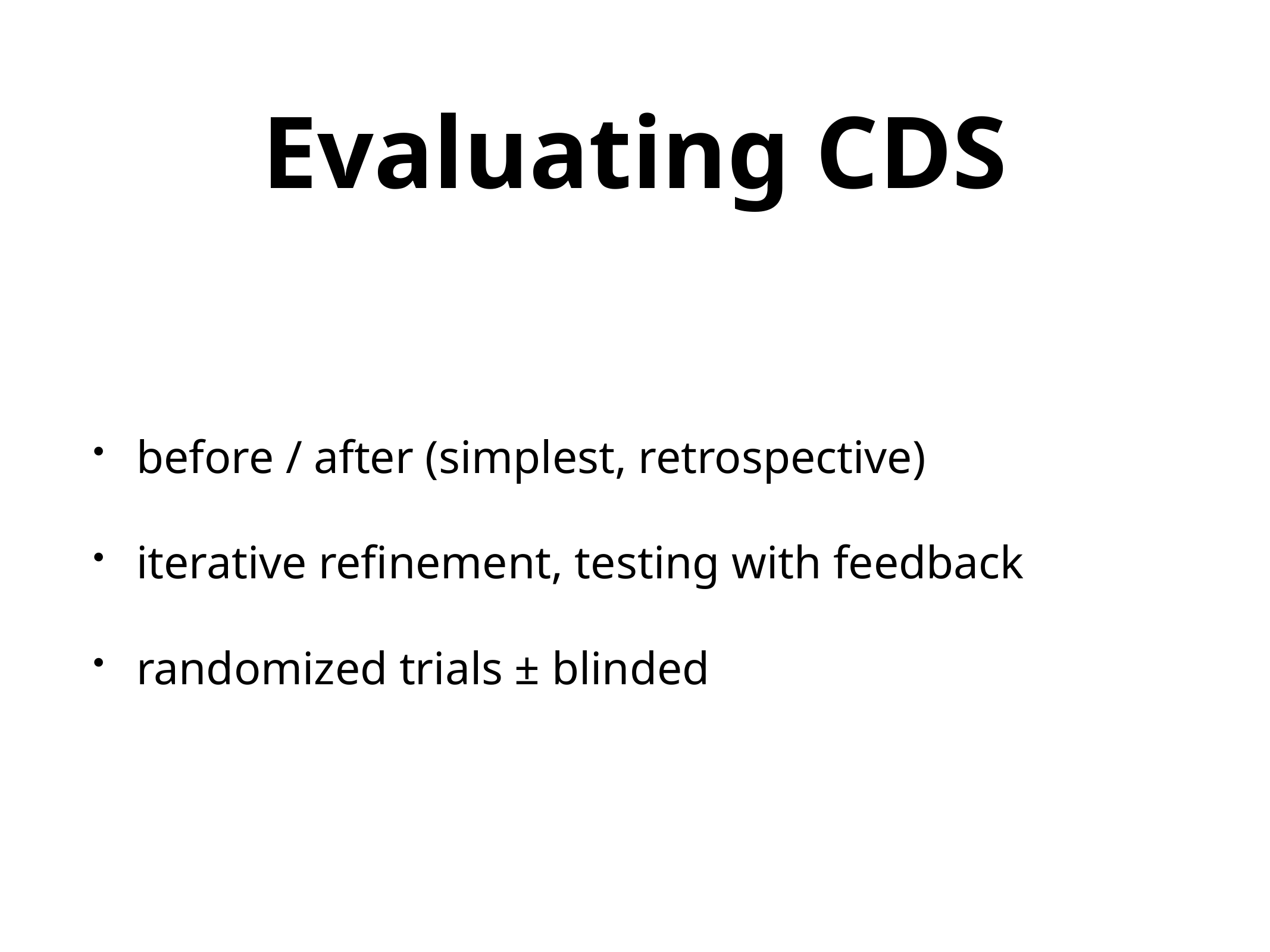

# Evaluating CDS
before / after (simplest, retrospective)
iterative refinement, testing with feedback
randomized trials ± blinded

## Slide 14
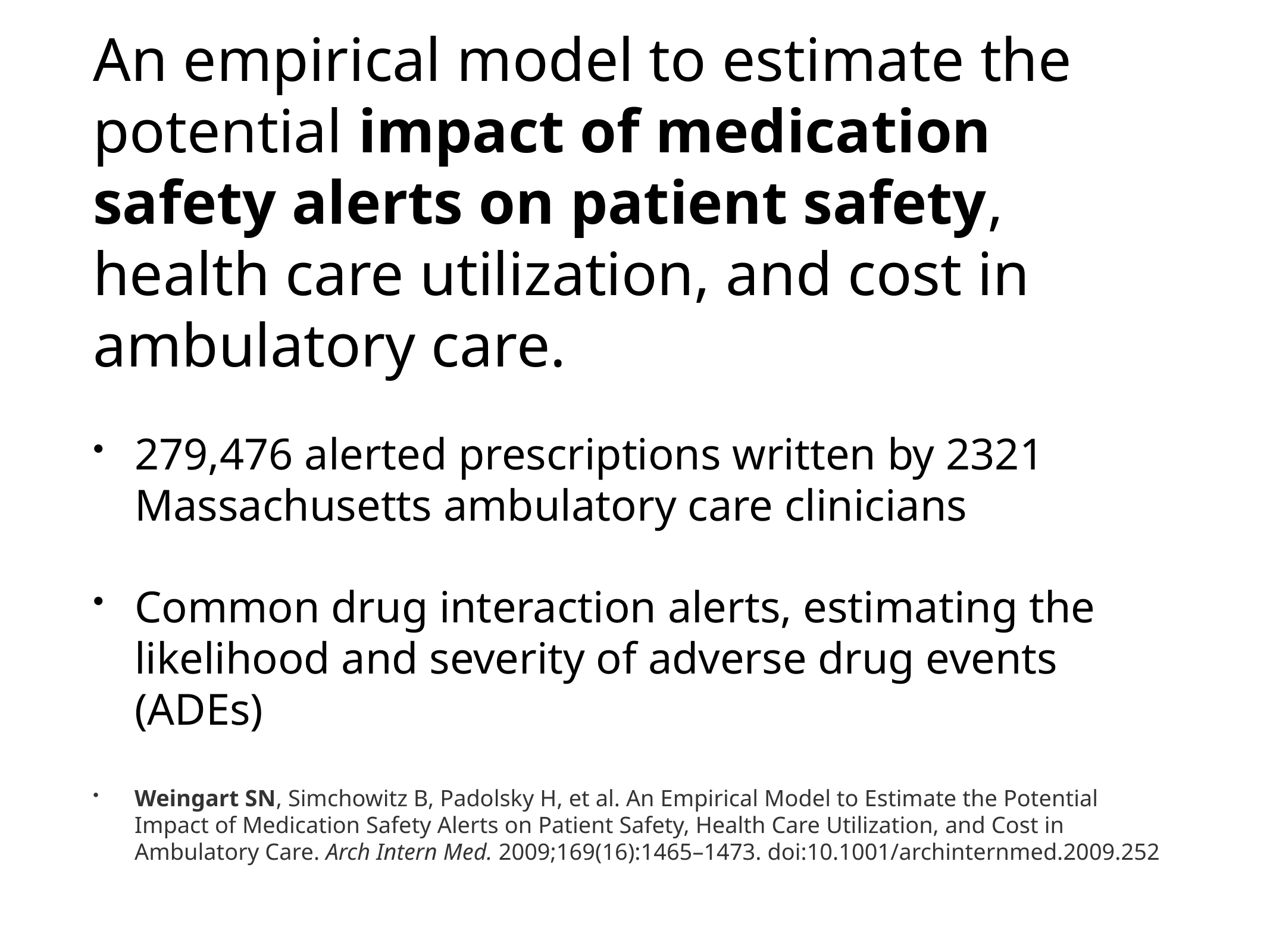

# An empirical model to estimate the potential impact of medication safety alerts on patient safety, health care utilization, and cost in ambulatory care.
279,476 alerted prescriptions written by 2321 Massachusetts ambulatory care clinicians
Common drug interaction alerts, estimating the likelihood and severity of adverse drug events (ADEs)
Weingart SN, Simchowitz B, Padolsky H, et al. An Empirical Model to Estimate the Potential Impact of Medication Safety Alerts on Patient Safety, Health Care Utilization, and Cost in Ambulatory Care. Arch Intern Med. 2009;169(16):1465–1473. doi:10.1001/archinternmed.2009.252

## Slide 15
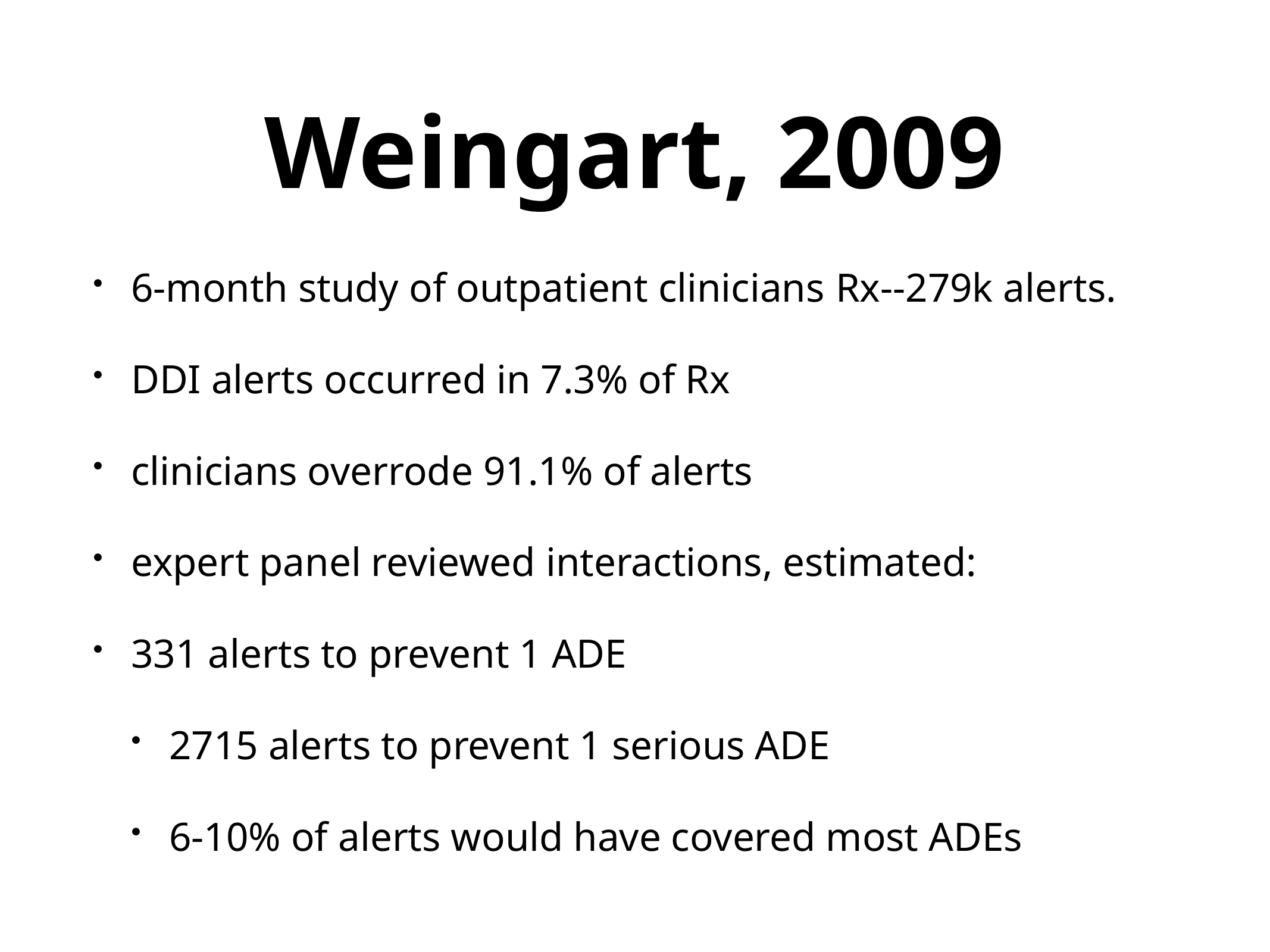

# Weingart, 2009
6-month study of outpatient clinicians Rx--279k alerts.
DDI alerts occurred in 7.3% of Rx
clinicians overrode 91.1% of alerts
expert panel reviewed interactions, estimated:
331 alerts to prevent 1 ADE
2715 alerts to prevent 1 serious ADE
6-10% of alerts would have covered most ADEs

## Slide 16
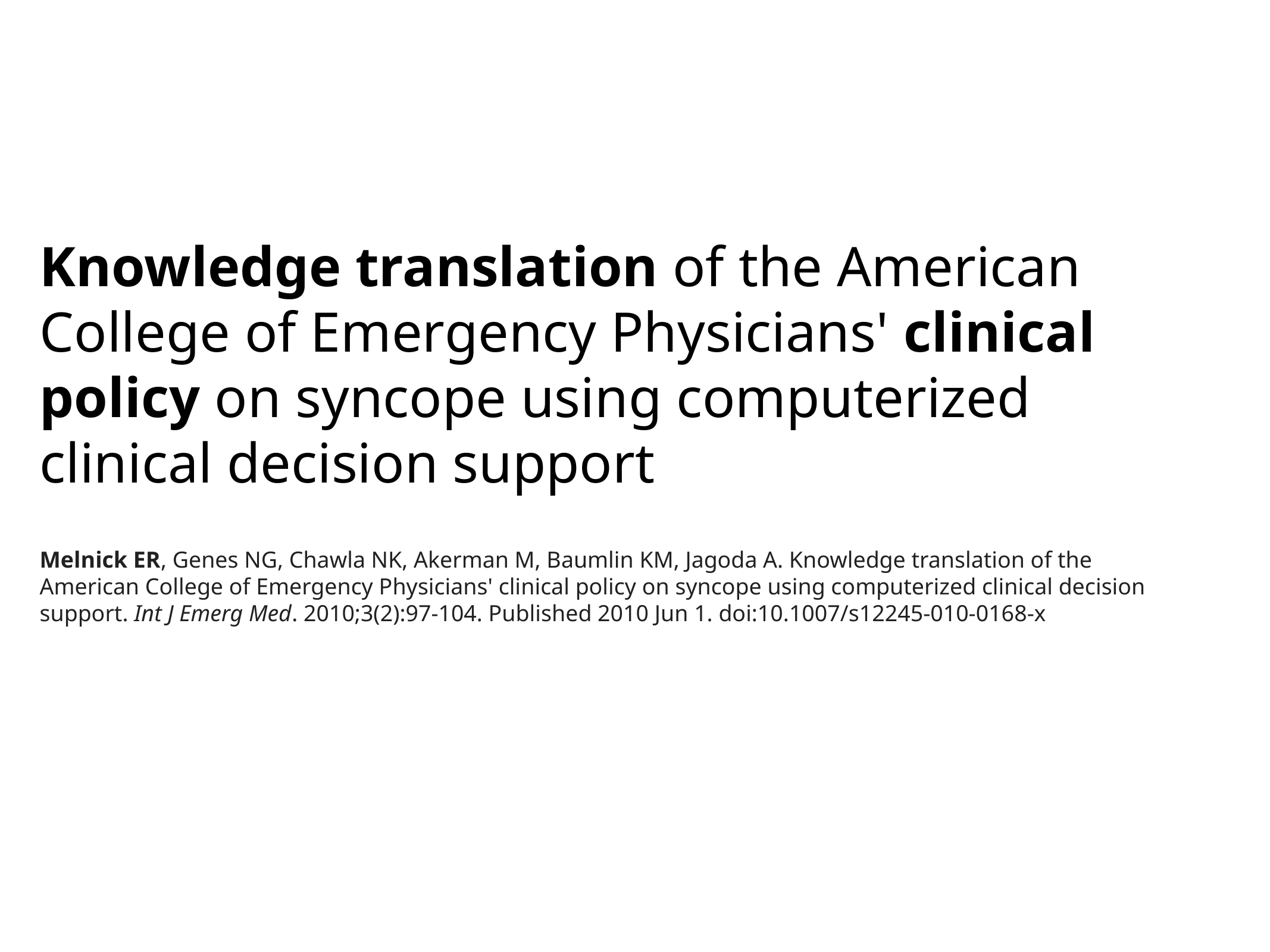

Knowledge translation of the American College of Emergency Physicians' clinical policy on syncope using computerized clinical decision support
Melnick ER, Genes NG, Chawla NK, Akerman M, Baumlin KM, Jagoda A. Knowledge translation of the American College of Emergency Physicians' clinical policy on syncope using computerized clinical decision support. Int J Emerg Med. 2010;3(2):97-104. Published 2010 Jun 1. doi:10.1007/s12245-010-0168-x

## Slide 17
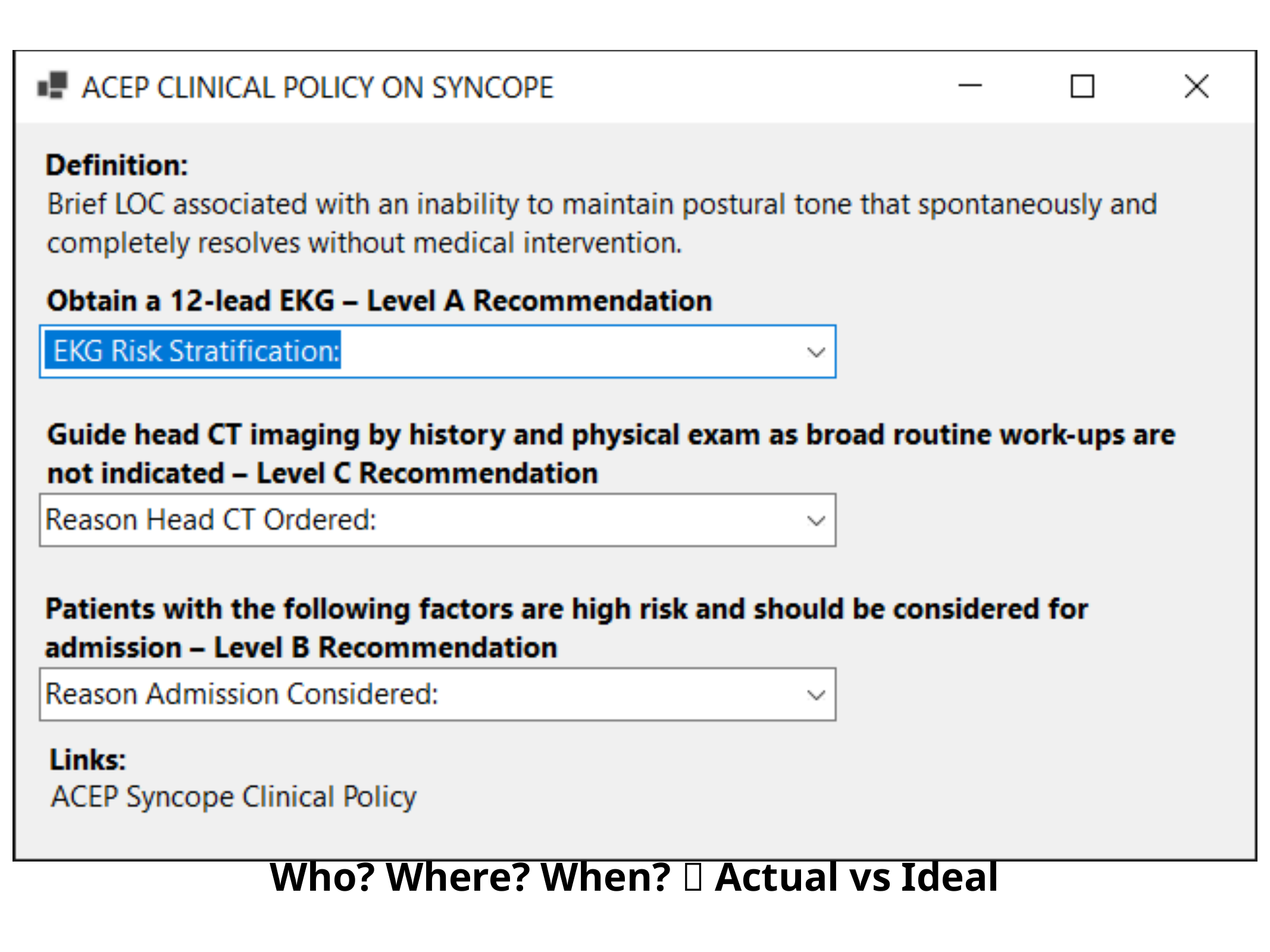

Who? Where? When?  Actual vs Ideal

## Slide 18
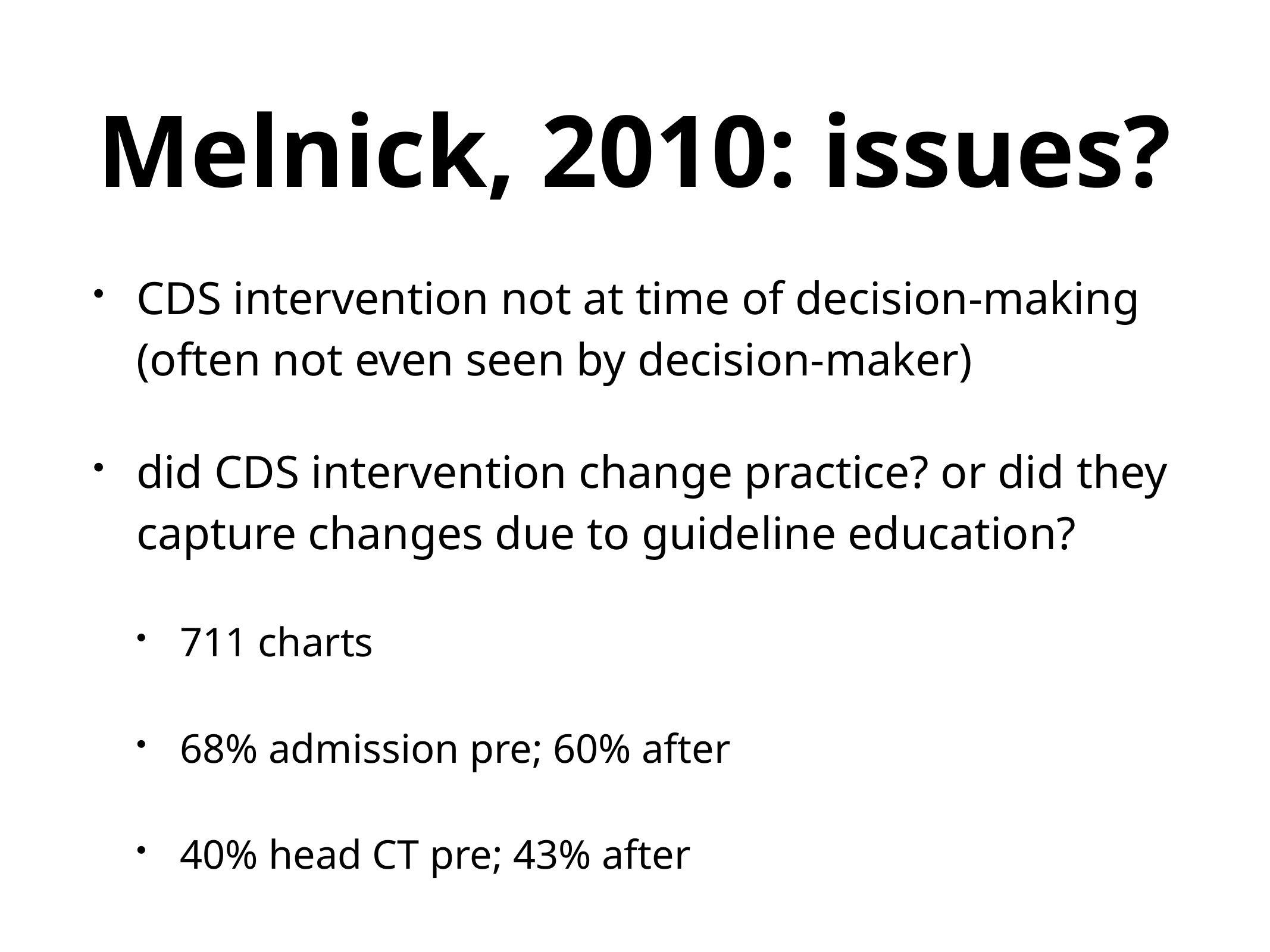

# Melnick, 2010: issues?
CDS intervention not at time of decision-making (often not even seen by decision-maker)
did CDS intervention change practice? or did they capture changes due to guideline education?
711 charts
68% admission pre; 60% after
40% head CT pre; 43% after

## Slide 19
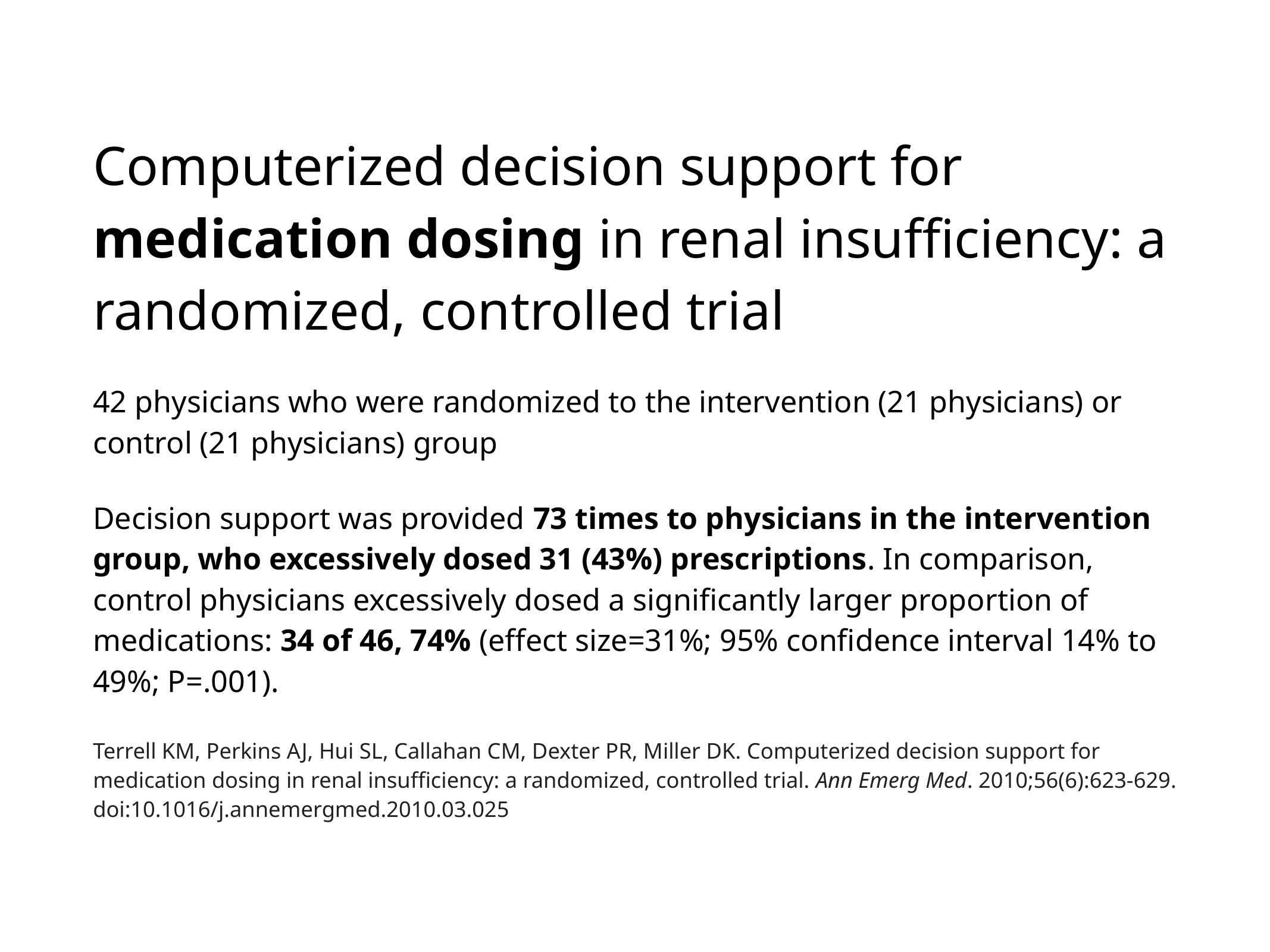

Computerized decision support for medication dosing in renal insufficiency: a randomized, controlled trial
42 physicians who were randomized to the intervention (21 physicians) or control (21 physicians) group
Decision support was provided 73 times to physicians in the intervention group, who excessively dosed 31 (43%) prescriptions. In comparison, control physicians excessively dosed a significantly larger proportion of medications: 34 of 46, 74% (effect size=31%; 95% confidence interval 14% to 49%; P=.001).
Terrell KM, Perkins AJ, Hui SL, Callahan CM, Dexter PR, Miller DK. Computerized decision support for medication dosing in renal insufficiency: a randomized, controlled trial. Ann Emerg Med. 2010;56(6):623-629. doi:10.1016/j.annemergmed.2010.03.025

## Slide 20
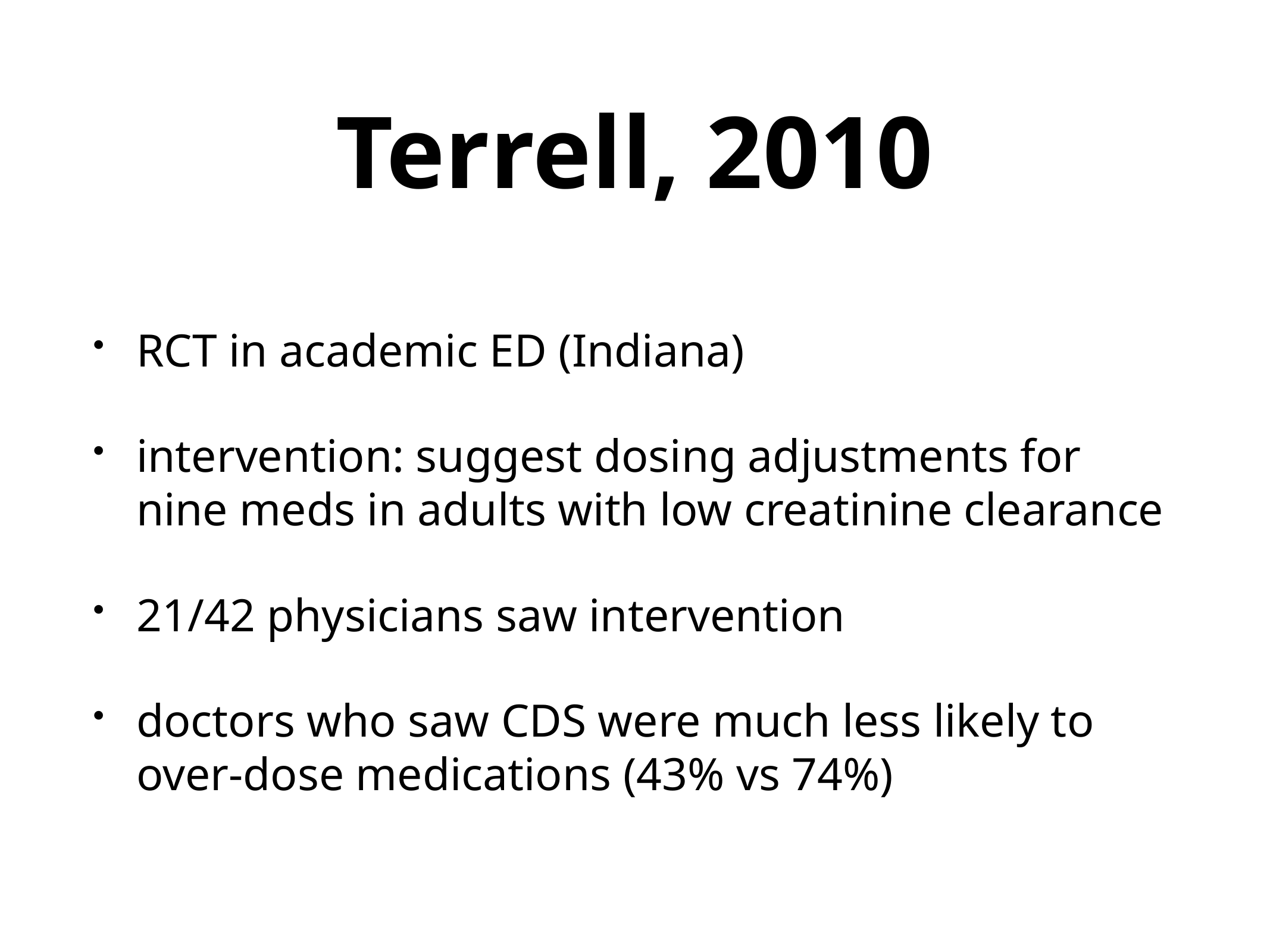

# Terrell, 2010
RCT in academic ED (Indiana)
intervention: suggest dosing adjustments for nine meds in adults with low creatinine clearance
21/42 physicians saw intervention
doctors who saw CDS were much less likely to over-dose medications (43% vs 74%)

## Slide 21
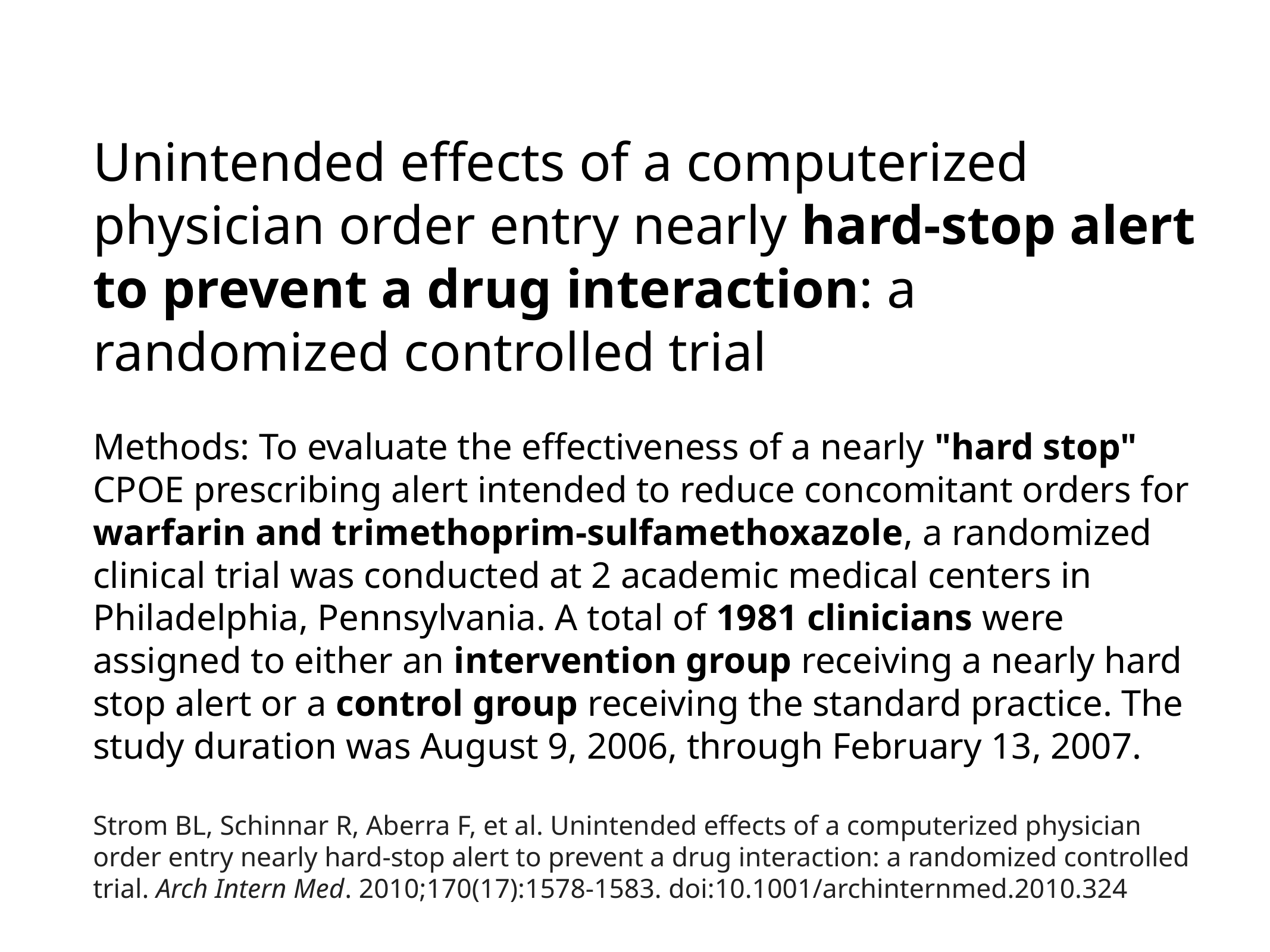

Unintended effects of a computerized physician order entry nearly hard-stop alert to prevent a drug interaction: a randomized controlled trial
Methods: To evaluate the effectiveness of a nearly "hard stop" CPOE prescribing alert intended to reduce concomitant orders for warfarin and trimethoprim-sulfamethoxazole, a randomized clinical trial was conducted at 2 academic medical centers in Philadelphia, Pennsylvania. A total of 1981 clinicians were assigned to either an intervention group receiving a nearly hard stop alert or a control group receiving the standard practice. The study duration was August 9, 2006, through February 13, 2007.
Strom BL, Schinnar R, Aberra F, et al. Unintended effects of a computerized physician order entry nearly hard-stop alert to prevent a drug interaction: a randomized controlled trial. Arch Intern Med. 2010;170(17):1578-1583. doi:10.1001/archinternmed.2010.324

## Slide 22
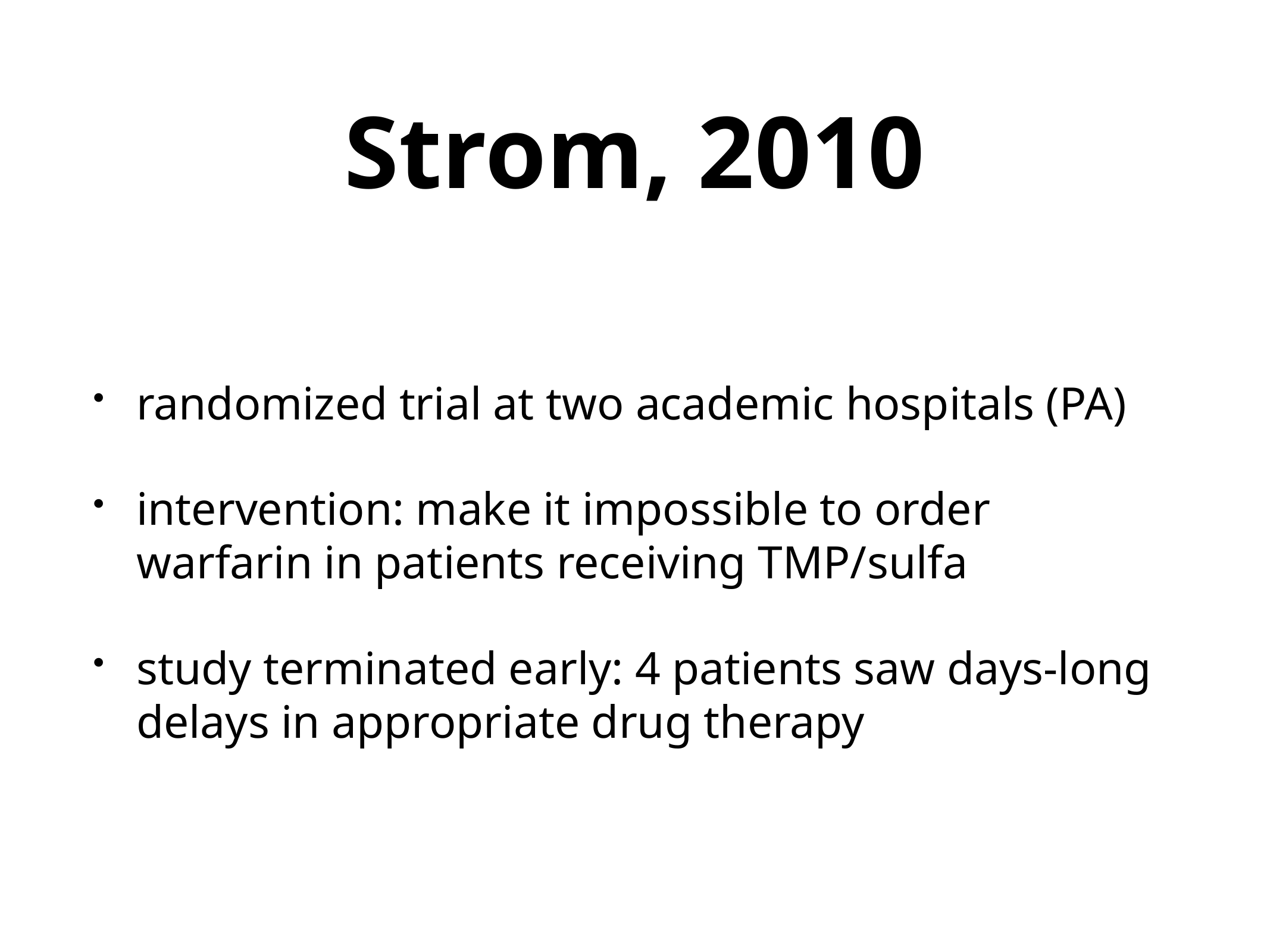

# Strom, 2010
randomized trial at two academic hospitals (PA)
intervention: make it impossible to order warfarin in patients receiving TMP/sulfa
study terminated early: 4 patients saw days-long delays in appropriate drug therapy

## Slide 23
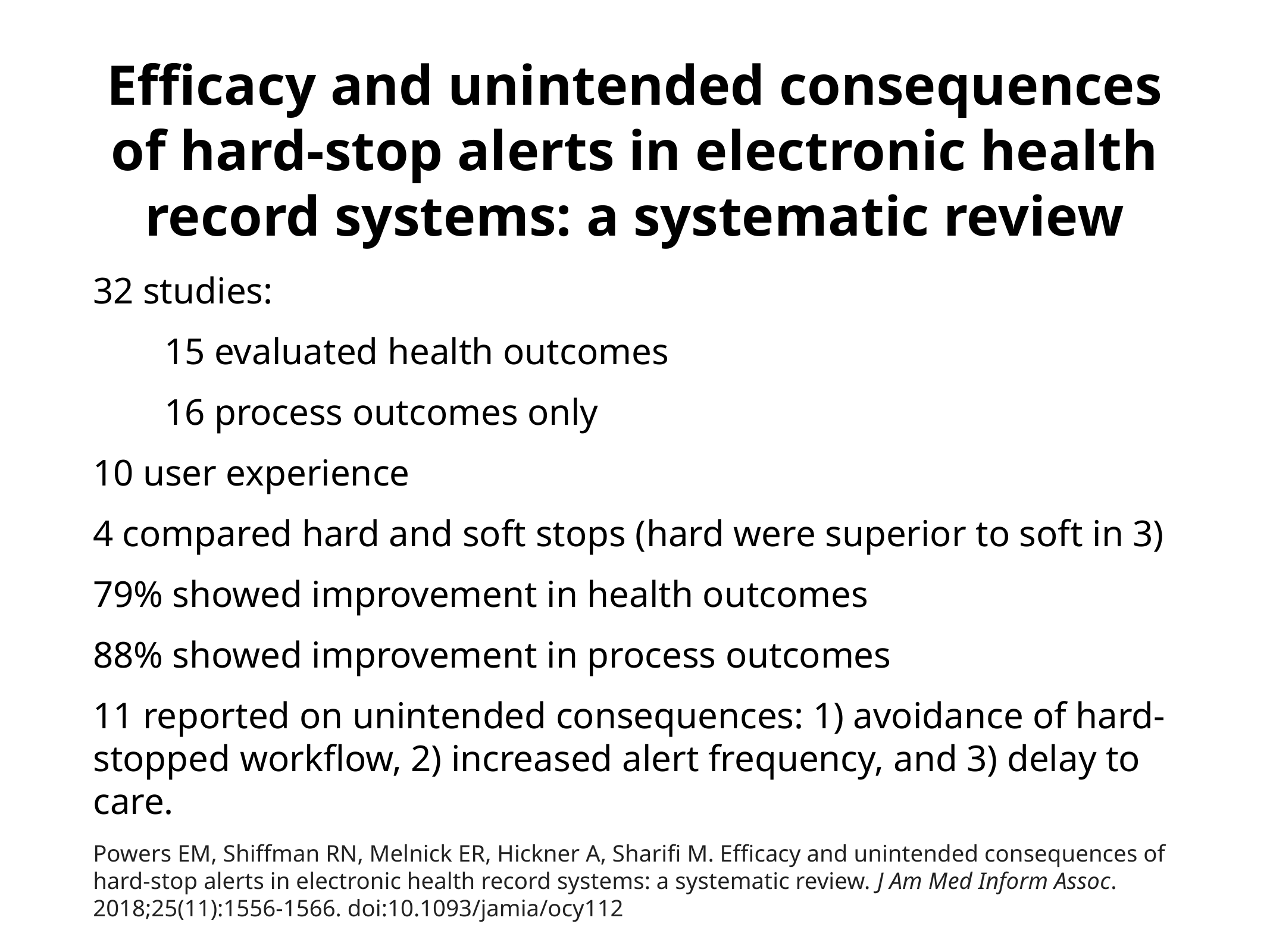

# Efficacy and unintended consequences of hard-stop alerts in electronic health record systems: a systematic review
32 studies:
	15 evaluated health outcomes
	16 process outcomes only
10 user experience
4 compared hard and soft stops (hard were superior to soft in 3)
79% showed improvement in health outcomes
88% showed improvement in process outcomes
11 reported on unintended consequences: 1) avoidance of hard-stopped workflow, 2) increased alert frequency, and 3) delay to care.
Powers EM, Shiffman RN, Melnick ER, Hickner A, Sharifi M. Efficacy and unintended consequences of hard-stop alerts in electronic health record systems: a systematic review. J Am Med Inform Assoc. 2018;25(11):1556-1566. doi:10.1093/jamia/ocy112

## Slide 24
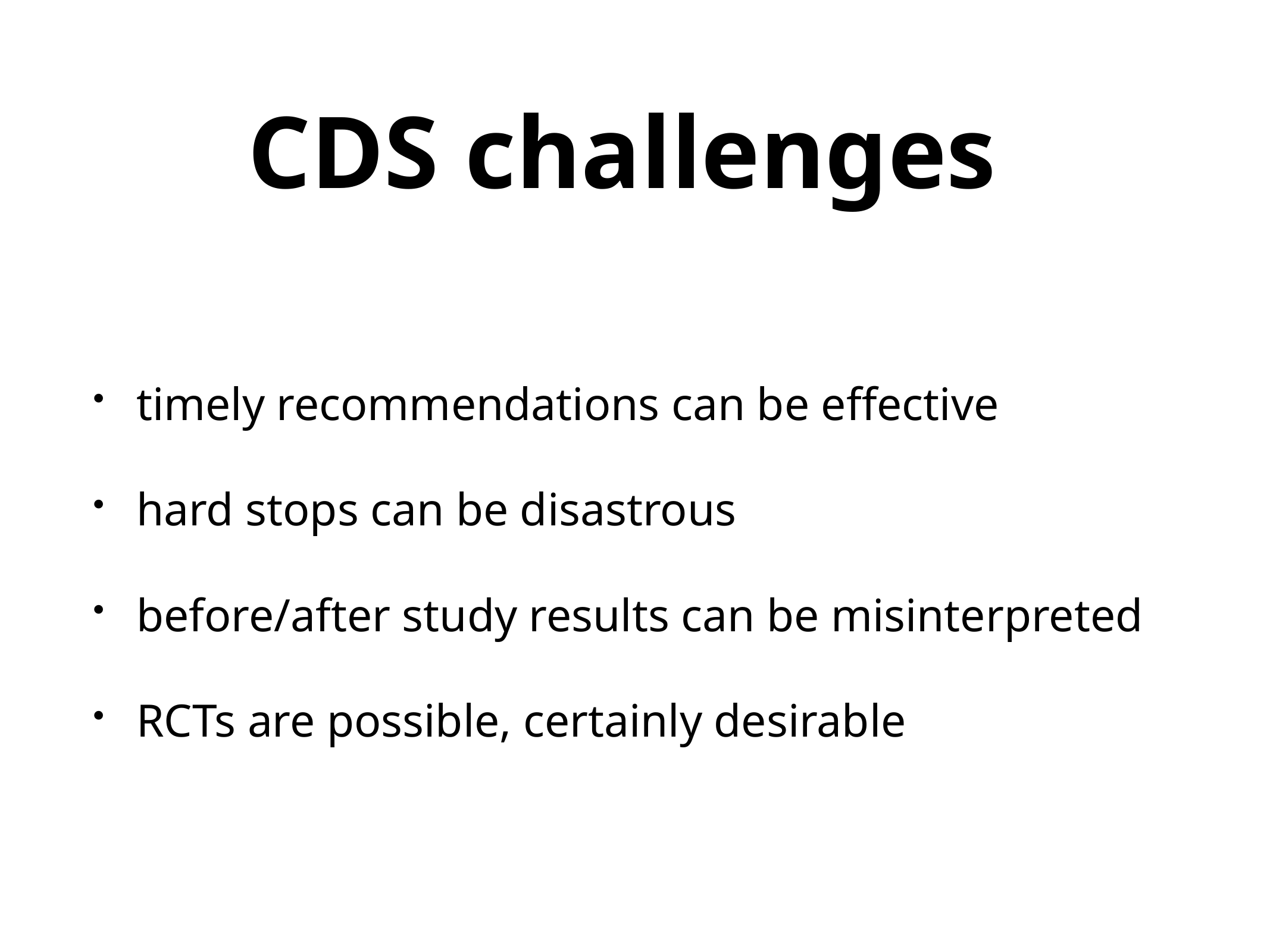

# CDS challenges
timely recommendations can be effective
hard stops can be disastrous
before/after study results can be misinterpreted
RCTs are possible, certainly desirable

## Slide 25
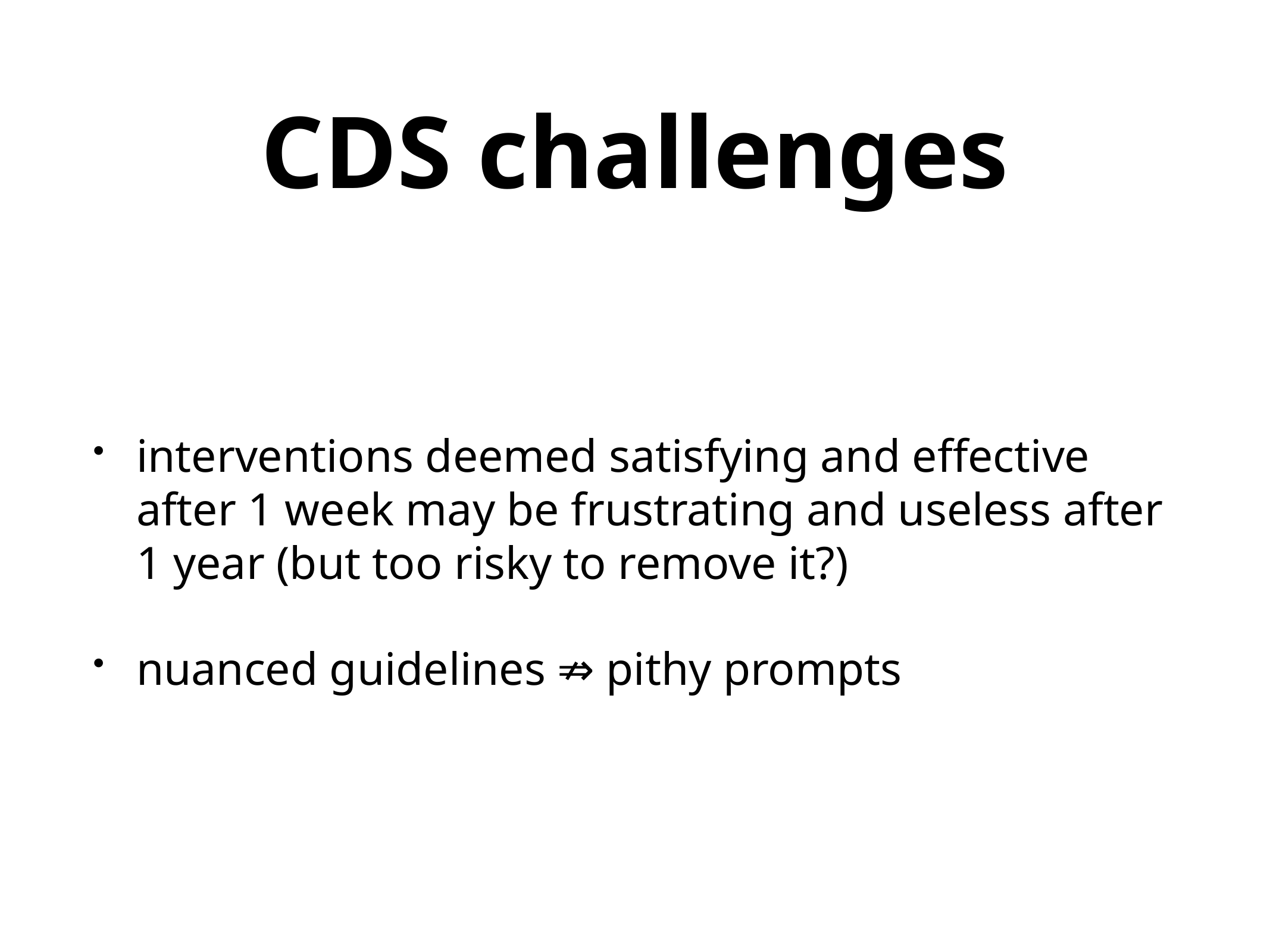

# CDS challenges
interventions deemed satisfying and effective after 1 week may be frustrating and useless after 1 year (but too risky to remove it?)
nuanced guidelines ⇏ pithy prompts

## Slide 26
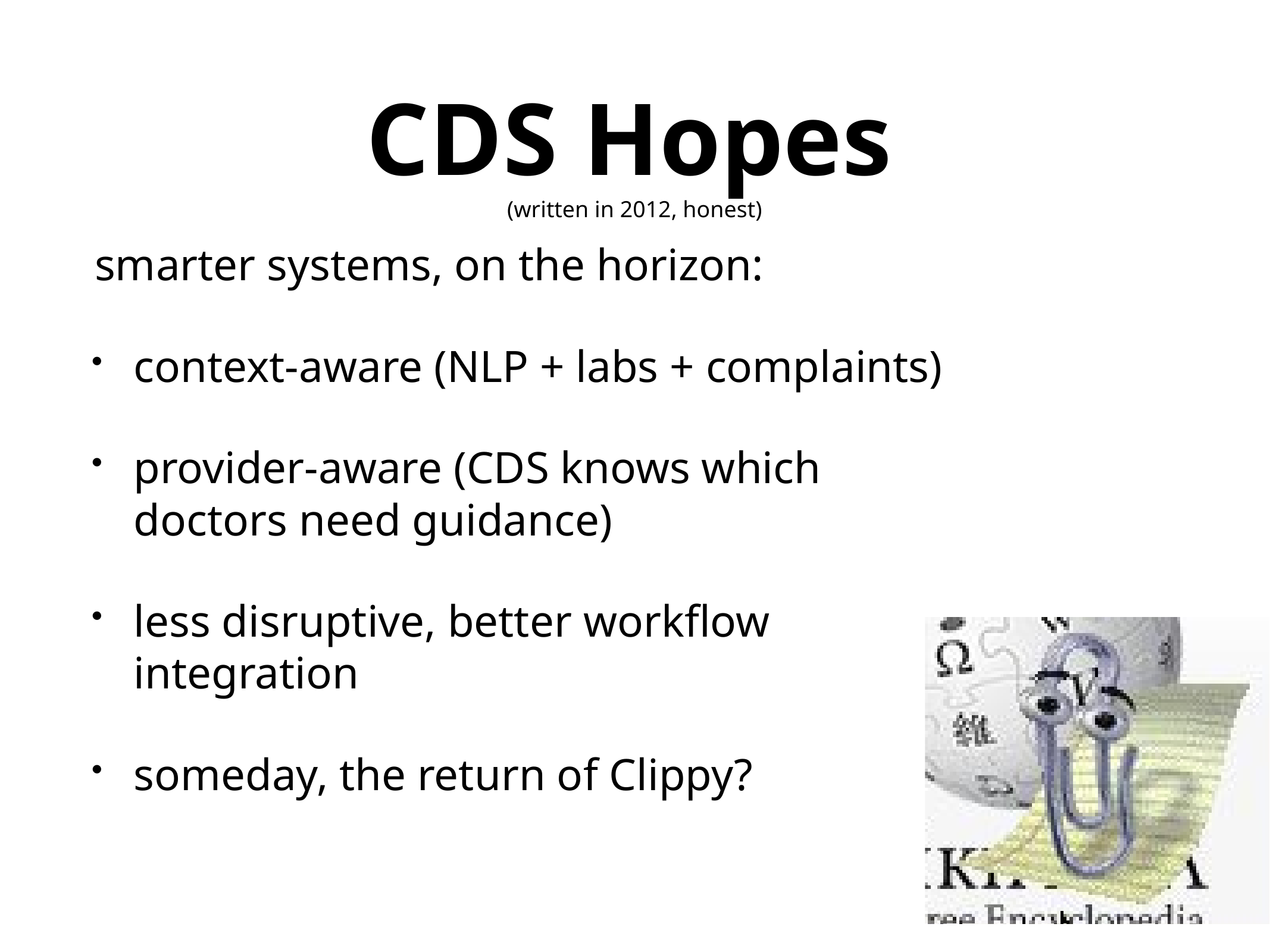

# CDS Hopes (written in 2012, honest)
 smarter systems, on the horizon:
context-aware (NLP + labs + complaints)
provider-aware (CDS knows which doctors need guidance)
less disruptive, better workflow integration
someday, the return of Clippy?

## Slide 27
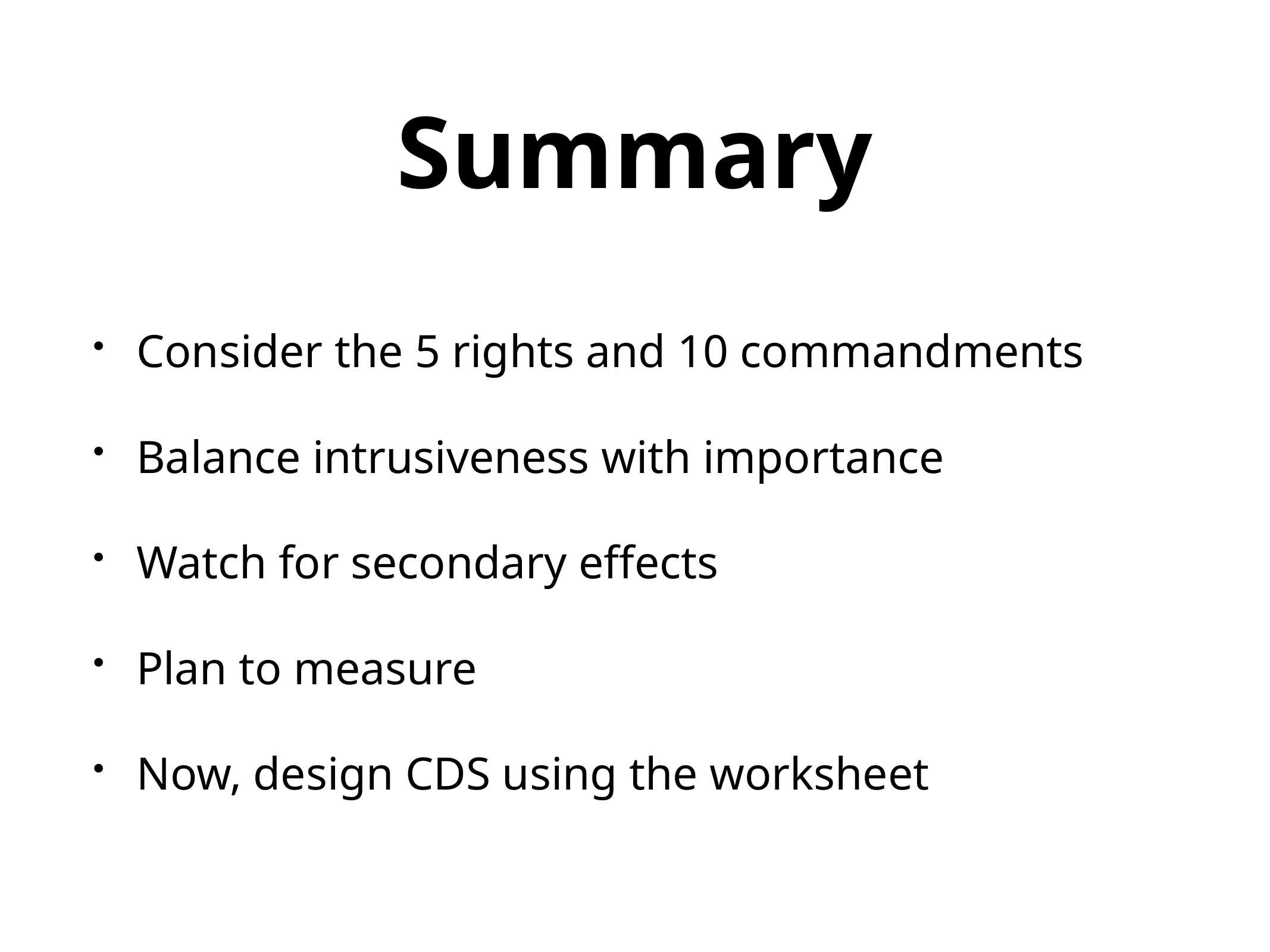

# Summary
Consider the 5 rights and 10 commandments
Balance intrusiveness with importance
Watch for secondary effects
Plan to measure
Now, design CDS using the worksheet
