# Supplementary material for: A Model Curriculum for an Emergency Medicine Residency Rotation in Clinical Informatics
Source: J Educ Teach Emerg Med. 2022 Oct 15;7(4):C1–C50. doi: 10.21980/J82P9H (PMC10332664; doi:10.21980/J82P9H)
Supplement: Supplementary file 11 [file JETem-7-4-C1-AppendixE2c.docx]

Appendix E.2.b:

Care Delivery Outcomes CDS Form

Select a topic:

List Stakeholders:

Define the right information:

1. What should the alert or change be?
2. What should the suggested outcome be?
3. What science/recommendation is CDS based on?

Define the Right Intervention format: (soft stop/hard stop/notification/precheck/order set/protocol/ info buttons):

Define the Right Channel (EHR/flowsheet/Order Entry)

Define the Right Person to receive CDS:

Define the Right Time in the Workflow (what is the trigger):

Write the clinical scenario:

Describe possible unintended consequences:

Using the following framework, begin drafting a Team Charter: from Schleyer T, Zappone S, Wells-Meyers C, Saxton T. Effective Interdisciplinary Teams. In: Finnell JT, Dixon BE, eds. *Clinical Informatics Study Guide.* 2nd ed. Springer; 2022: 285-306. You will present this at the end of the final session.

| **Team Charter** | |
| --- | --- |
| **Purpose:** Why does a team exist?  What is it expected to accomplish? |  |
| **1. Statement of Work:** Accomplish, Expected outcomes |  |
| **2. Duration:** Timeline |  |
| **3. Scope:** In/Out |  |
| **4. End result:** |  |
| **Members:** |  |
| **5: Team**, Team Leads, Members |  |
| **6. External stakeholders:** Who else might benefit or be affected by this? |  |
| **Structure and Process:** |  |
| **7. Roles and responsibilities:** |  |
| **8. Meeting plan:** |  |
| **9. Reporting plan:** |  |
| **10. Deliverables and Timetable:** |  |
| **Resources:** |  |
| **11. Financial Resources:** |  |
| **12. Technological Resources:** |  |
| **13. Support Resources:** |  |
