# Supplementary material for: A Model Curriculum for an Emergency Medicine Residency Rotation in Clinical Informatics
Source: J Educ Teach Emerg Med. 2022 Oct 15;7(4):C1–C50. doi: 10.21980/J82P9H (PMC10332664; doi:10.21980/J82P9H)
Supplement: Supplementary file 12 [file JETem-7-4-C1-AppendixE3a.docx]

Appendix E.3:

Small Group Discussion: Data Analytics and Governance

**Pre-Session Preparation:**

Data Analytics and Governance content will be reviewed during the third week of the rotation. The small group discussion will be held at the end of the week. It begins with a slide presentation and follows with discussion of four scenarios covering issues in data analytics and data governance. The 20-minute presentation is found in “Appendix E.3.a. Data Analytics Governance PPT.” A framework for a 40-minute small group discussion and answers are included in the documents, “Appendix E.3.b. Data Analytics Governance Instructor Material” and “Appendix E.3.c. Data Analytics Governance Learner Material.”

**Recommended Pre-Reading:**

1. Finnell JT, Dixon BE, eds. *Clinical Informatics Study Guide.* Springer; 2022.
   1. Kasthurirathne SN, Grannis SJ. Analytics. In: Finnell JT, Dixon BE, eds. *Clinical Informatics Study Guide.* Springer; 2022: 227.
   2. Dixon BE, Holmgren AJ, Adler-Milstein J, Grannis SJ. Health Information Exchange and Interoperability. In: Finnell JT, Dixon BE, eds. *Clinical Informatics Study Guide.* Springer; 2022:203-219.
2. Hersh WR. Data Science, Machine Learning, and Artificial Intelligence. In: Hersh WR, ed. *Health Informatics: Practical Guide*. 8^th^ ed. Informatics Education. 2022;89-115.
3. Rasmussen J. Health Information Privacy and Security. In: Hersh WR, ed. *Health Informatics: Practical Guide*. 8^th^ ed. Informatics Education. 2022:233-252.
4. Shapiro JS, Crowley D, Hoxhaj S, et al. Health Information Exchange in Emergency Medicine. *Ann Emerg Med*. 2016 Feb;67(2):216-26. Epub 2015 Jul 28. PMID: 26233924. doi: 10.1016/j.annemergmed.2015.06.018
5. Cimino JJ. Desiderata for controlled medical vocabularies in the twenty-first century. *Methods Inf Med*. 1998;37(4-5):394-403.
6. Hersh WR. Clinical Data. https://dmice.ohsu.edu/hersh/whatis/ Updated Jan 5, 2022. Accessed April 13, 2022. At: https://echo360.org/media/630e4d75-0024-4753-b9f2-a7475fbeff4f/public
7. McClay J. FHIR. ACEP Emergency Medicine Informatics Grand Rounds. Published Aug 2, 2016. Accessed April 12, 2022. At: <https://www.acep.org/administration/quality/health-information-technology/hit-tarticles/fhir-fast-health-information-resources/>
8. US Department of Health and Human Services, Office for Civil Rights. Breach Portal: Notice to the Secretary of HHS Breach of Unsecured Protected Health Information. Accessed April 12, 2022. At: https://ocrportal.hhs.gov/ocr/breach/breach_report.jsf
9. U.S. Department of Health & Human Services, Health Information Privacy. Cybersecurity Guidance Material. Accessed April 12, 2022. At: https://www.hhs.gov/hipaa/for-professionals/security/guidance/cybersecurity/index.html
10. Office of the National Coordinator for Health IT: Health IT Privacy and Security for Providers. Top 10 Tips for Cybersecurity in Health Care. Accessed April 12, 2022. At: https://www.healthit.gov/sites/default/files/Top_10_Tips_for_Cybersecurity.pdf

**Objectives:**

Residents will gain an introduction of the broad field of clinical informatics, with a focus on the key applications of informatics in emergency medicine. By the end of this rotation, the learner will be able to:

1. Understand the need for standards, clinical terminologies, and ontologies.
2. Recognize the need for data governance and analytics.

**Linked objectives and methods:**

Objectives are achieved through small group discussion with guidance from the small group instructor. This allows for knowledge translation in an informal setting. Learners can discuss their experiences and ideas in an open format. This presentation covers three objectives. Grounded in examples and discussion using the documents, “Appendix E.3.b. Data Analytics Governance Instructor Material,” and “Appendix E.3.c. Data Analytics Governance Learner Material,” learners will:

1. Develop a basic understanding of the concepts of data analytics for ED needs, including techniques of “AI”/machine learning and natural language processing.
2. Understand strategies for data warehouse access and methods of conducting research and quality projects to improve ED care and operations.
3. Appreciate the utility of health information exchange in the ED, as well as some of the challenges of interoperability, necessity of data standards, and tradeoffs involved with different consent models.

As described above, the instructor should be familiar with the cited studies and aspects of running queries and sharing data to facilitate discussion. The documents, “Appendix E.3.b. Data Analytics Governance Instructor Material,” and “Appendix E.3.c. Data Analytics Governance Learner Material,” serve as a discussion guide for the small group.
