# Supplementary material for: A Model Curriculum for an Emergency Medicine Residency Rotation in Clinical Informatics
Source: J Educ Teach Emerg Med. 2022 Oct 15;7(4):C1–C50. doi: 10.21980/J82P9H (PMC10332664; doi:10.21980/J82P9H)
Supplement: Supplementary file 13 — Please see associated PowerPoint file [file JETem-7-4-C1-AppendixE3b.pptx]

## Slide 1
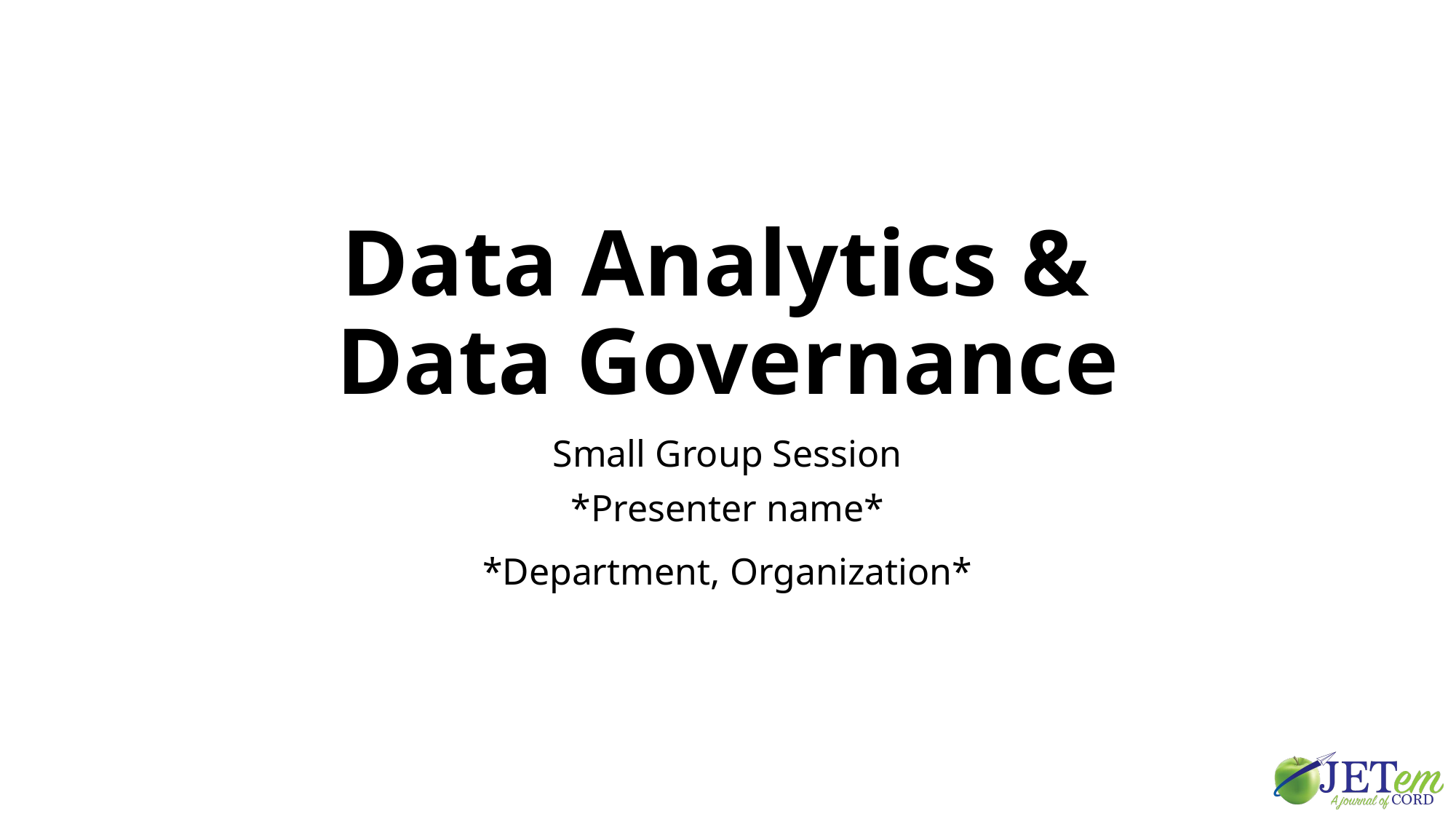

# Data Analytics & Data Governance
Small Group Session
*Presenter name*
*Department, Organization*

## Slide 2
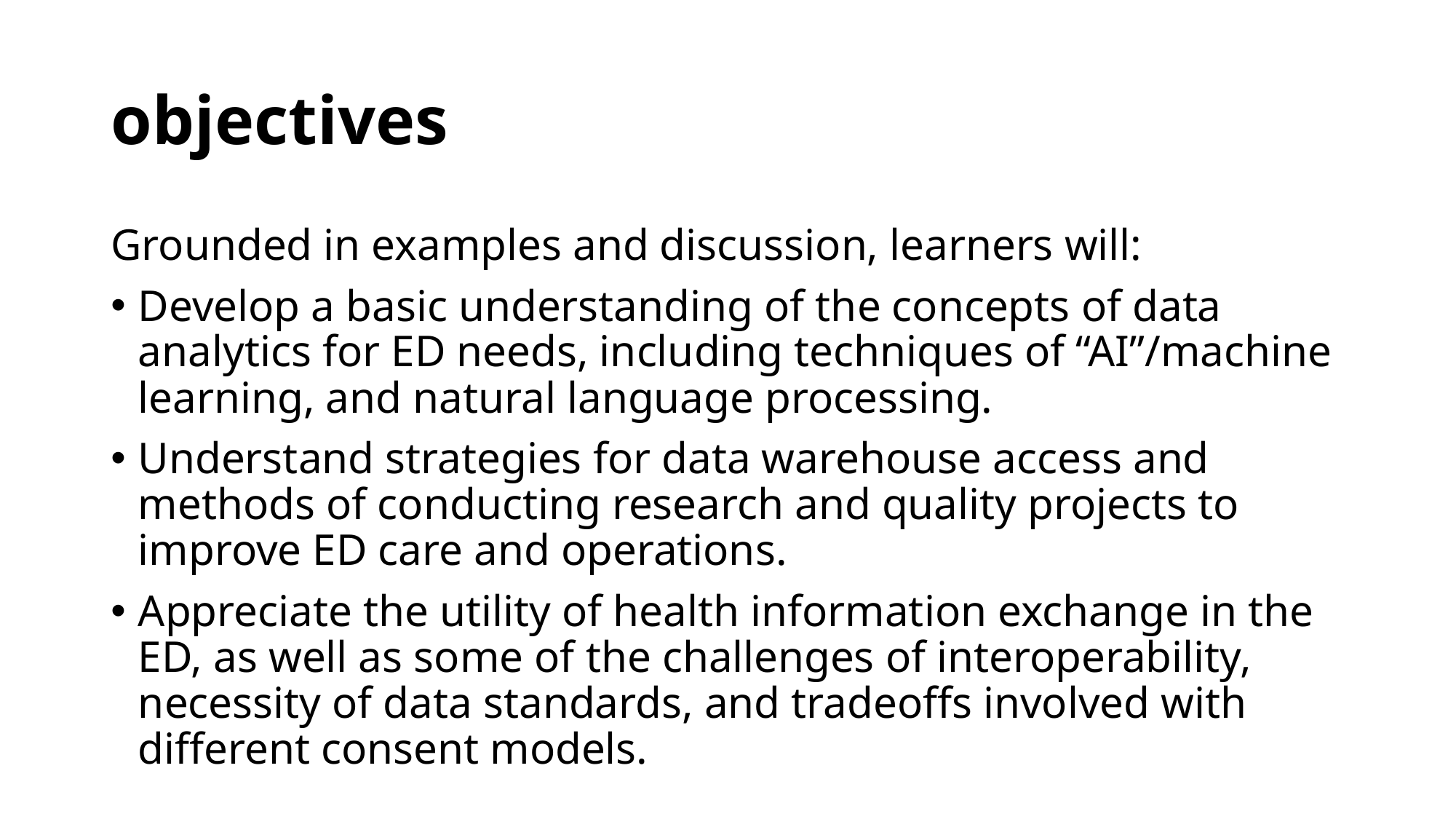

# objectives
Grounded in examples and discussion, learners will:
Develop a basic understanding of the concepts of data analytics for ED needs, including techniques of “AI”/machine learning, and natural language processing.
Understand strategies for data warehouse access and methods of conducting research and quality projects to improve ED care and operations.
Appreciate the utility of health information exchange in the ED, as well as some of the challenges of interoperability, necessity of data standards, and tradeoffs involved with different consent models.

## Slide 3
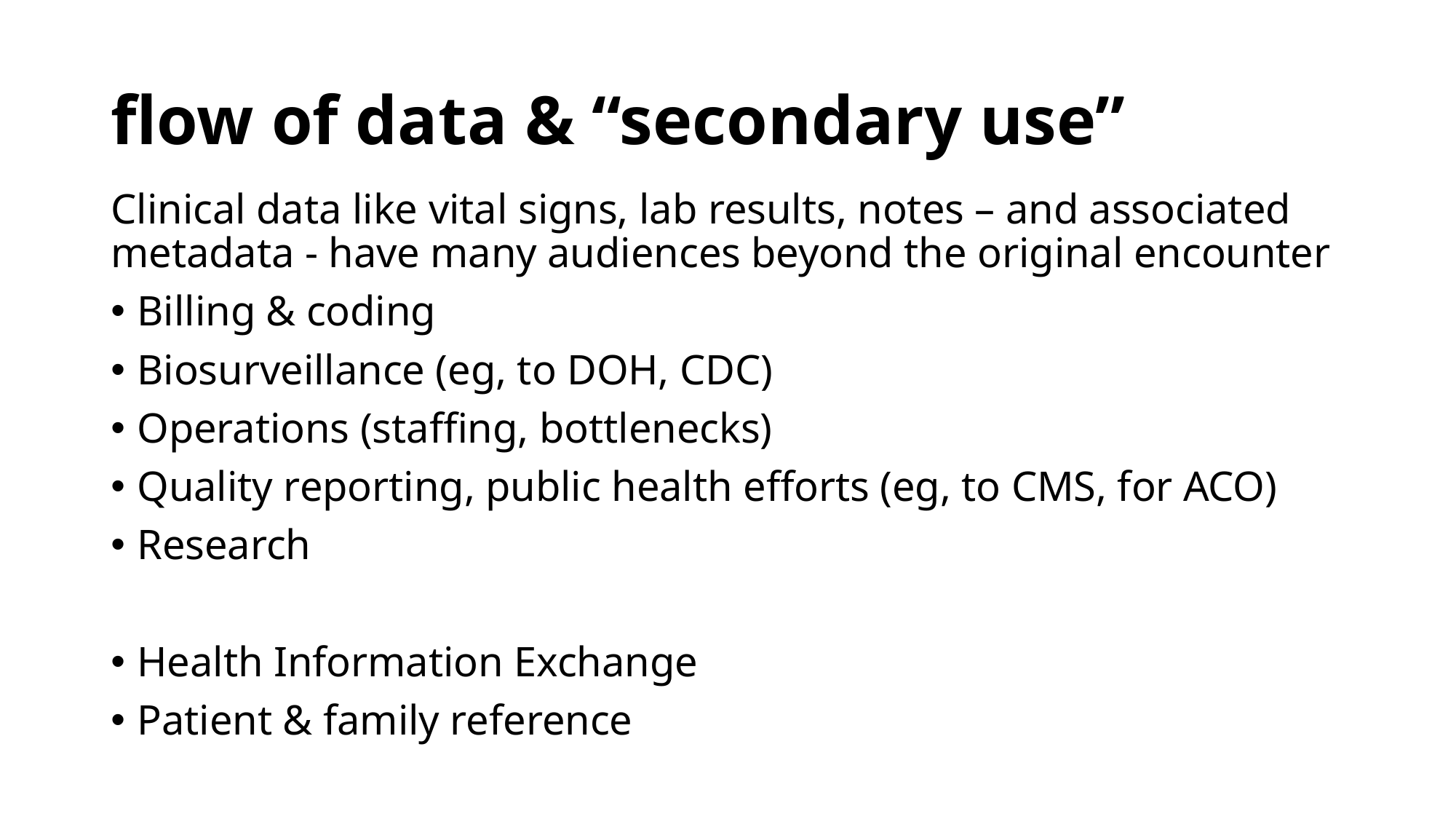

# flow of data & “secondary use”
Clinical data like vital signs, lab results, notes – and associated metadata - have many audiences beyond the original encounter
Billing & coding
Biosurveillance (eg, to DOH, CDC)
Operations (staffing, bottlenecks)
Quality reporting, public health efforts (eg, to CMS, for ACO)
Research
Health Information Exchange
Patient & family reference

## Slide 4
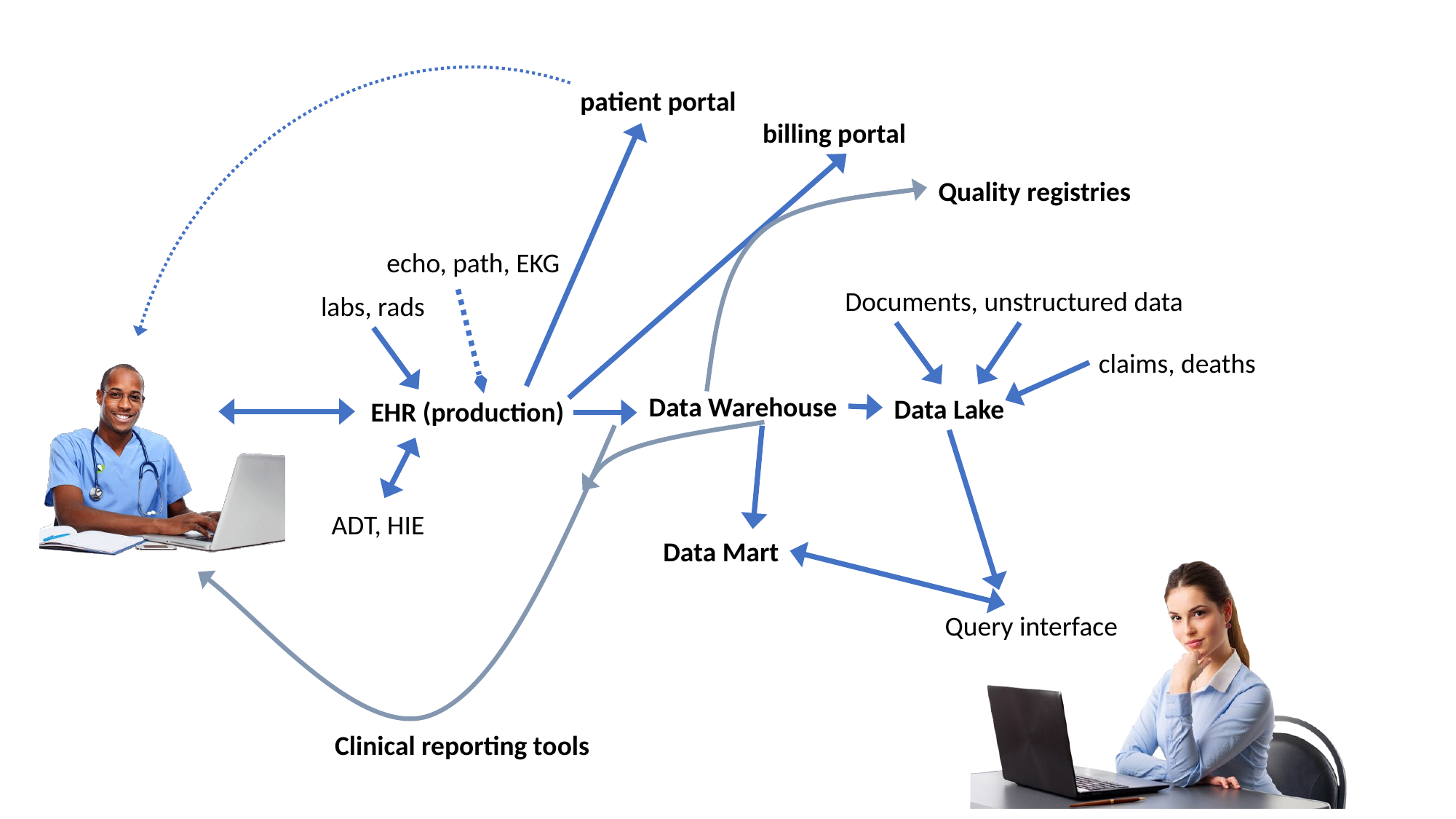

patient portal
billing portal
Quality registries
echo, path, EKG
Documents, unstructured data
labs, rads
claims, deaths
Data Warehouse
Data Lake
EHR (production)
ADT, HIE
Data Mart
Query interface
Clinical reporting tools

## Slide 5
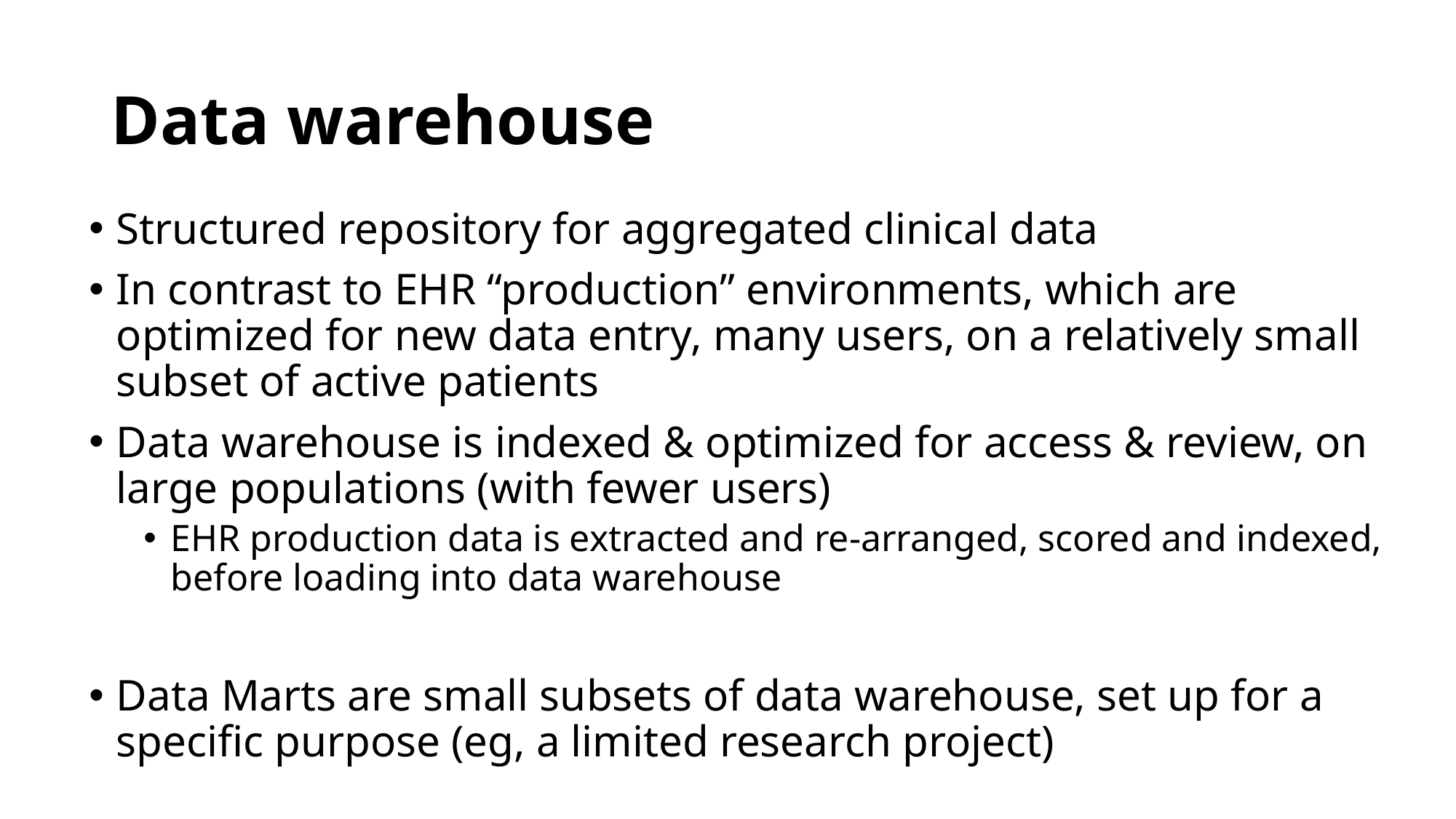

# Data warehouse
Structured repository for aggregated clinical data
In contrast to EHR “production” environments, which are optimized for new data entry, many users, on a relatively small subset of active patients
Data warehouse is indexed & optimized for access & review, on large populations (with fewer users)
EHR production data is extracted and re-arranged, scored and indexed, before loading into data warehouse
Data Marts are small subsets of data warehouse, set up for a specific purpose (eg, a limited research project)

## Slide 6
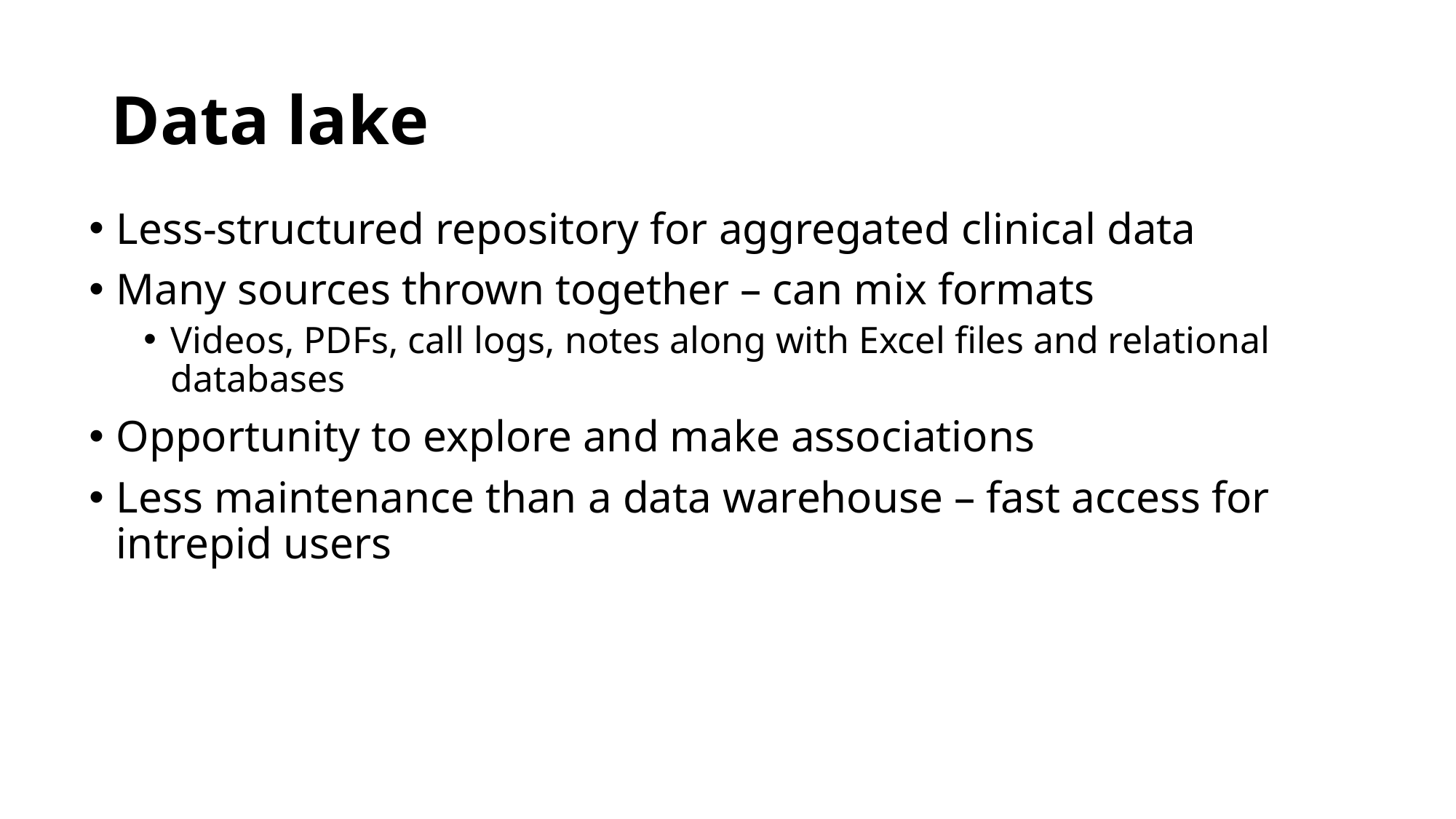

# Data lake
Less-structured repository for aggregated clinical data
Many sources thrown together – can mix formats
Videos, PDFs, call logs, notes along with Excel files and relational databases
Opportunity to explore and make associations
Less maintenance than a data warehouse – fast access for intrepid users

## Slide 7
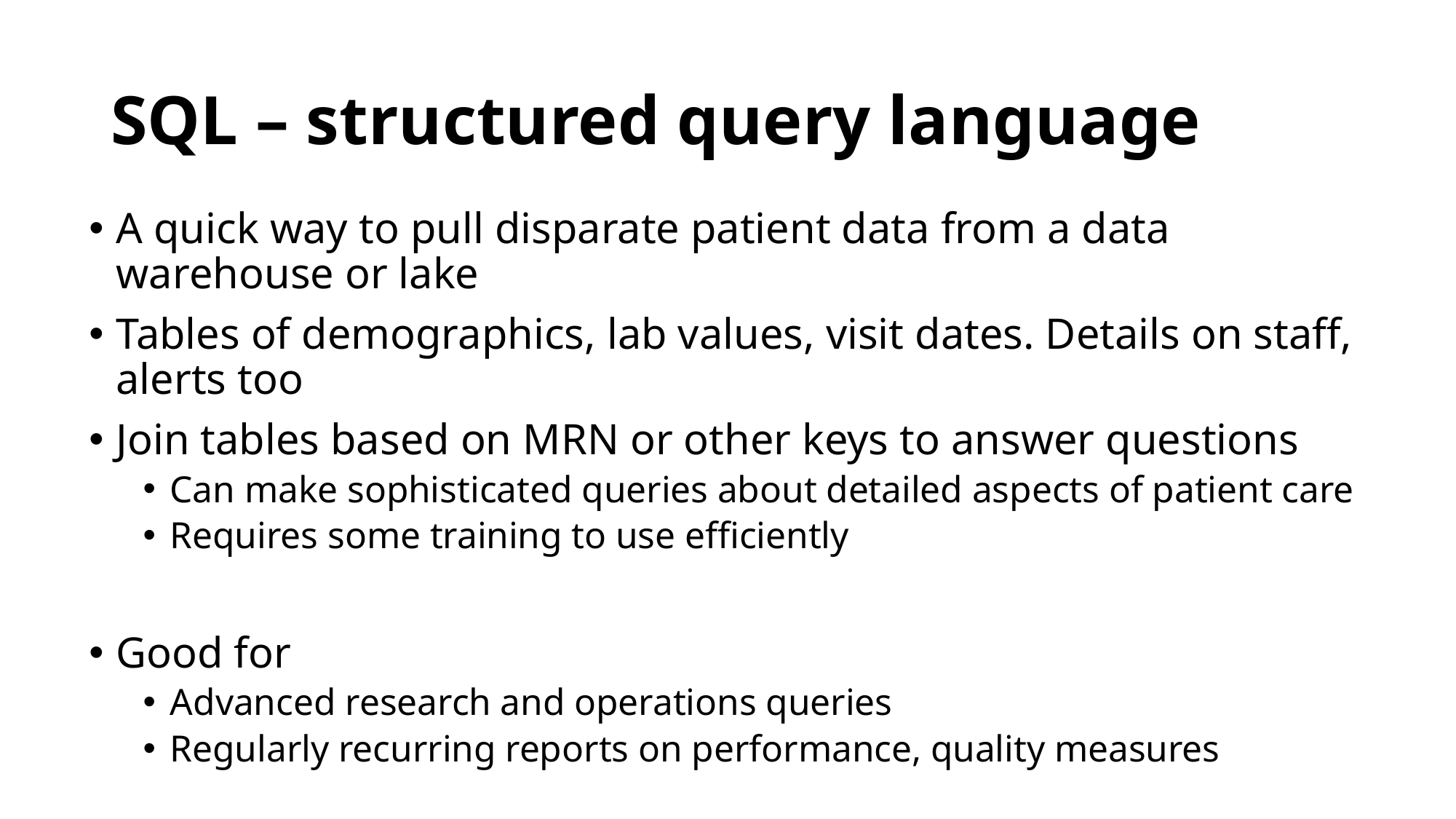

# SQL – structured query language
A quick way to pull disparate patient data from a data warehouse or lake
Tables of demographics, lab values, visit dates. Details on staff, alerts too
Join tables based on MRN or other keys to answer questions
Can make sophisticated queries about detailed aspects of patient care
Requires some training to use efficiently
Good for
Advanced research and operations queries
Regularly recurring reports on performance, quality measures

## Slide 8
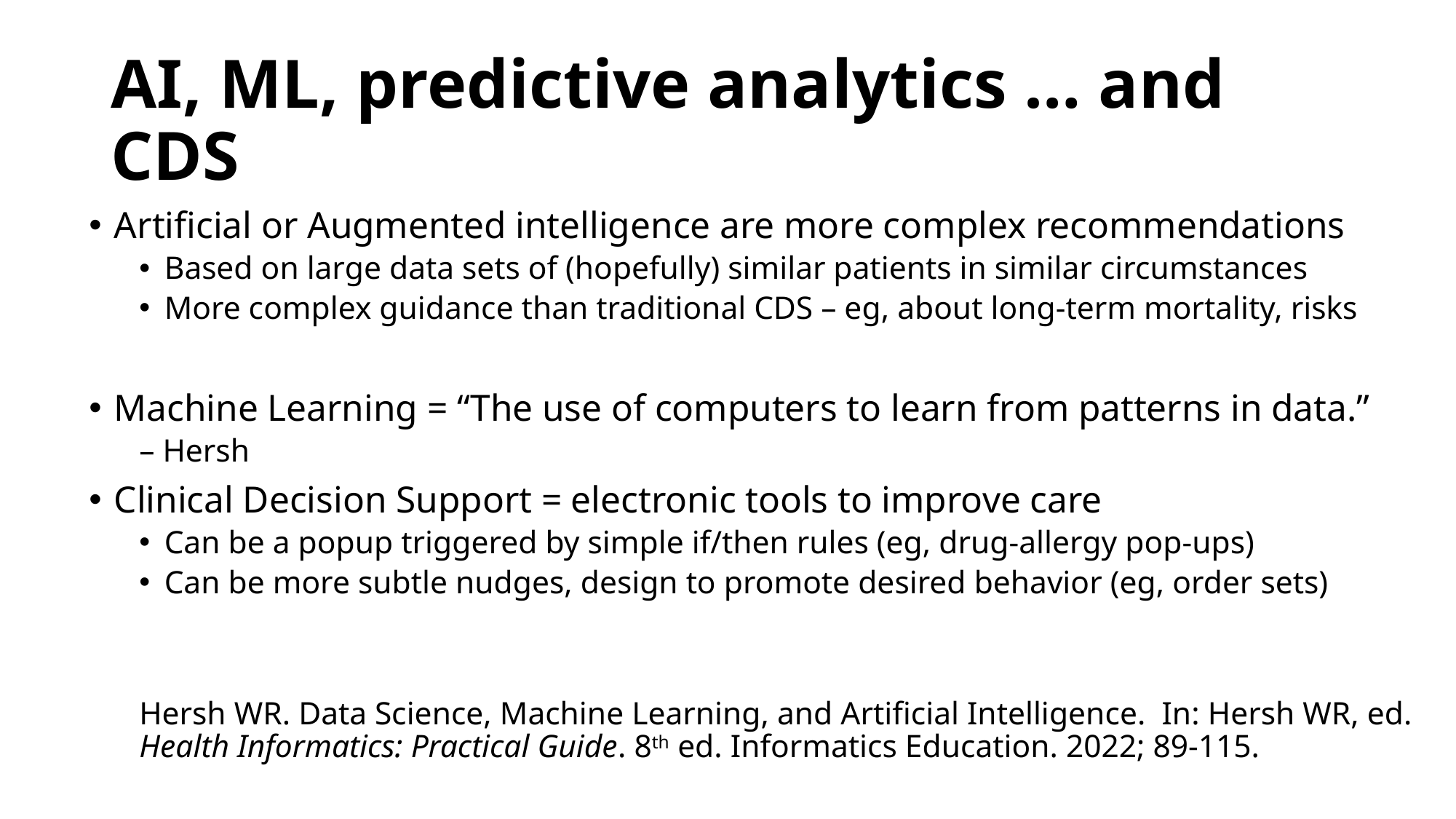

# AI, ML, predictive analytics … and CDS
Artificial or Augmented intelligence are more complex recommendations
Based on large data sets of (hopefully) similar patients in similar circumstances
More complex guidance than traditional CDS – eg, about long-term mortality, risks
Machine Learning = “The use of computers to learn from patterns in data.”
– Hersh
Clinical Decision Support = electronic tools to improve care
Can be a popup triggered by simple if/then rules (eg, drug-allergy pop-ups)
Can be more subtle nudges, design to promote desired behavior (eg, order sets)
Hersh WR. Data Science, Machine Learning, and Artificial Intelligence. In: Hersh WR, ed. Health Informatics: Practical Guide. 8th ed. Informatics Education. 2022; 89-115.

## Slide 9
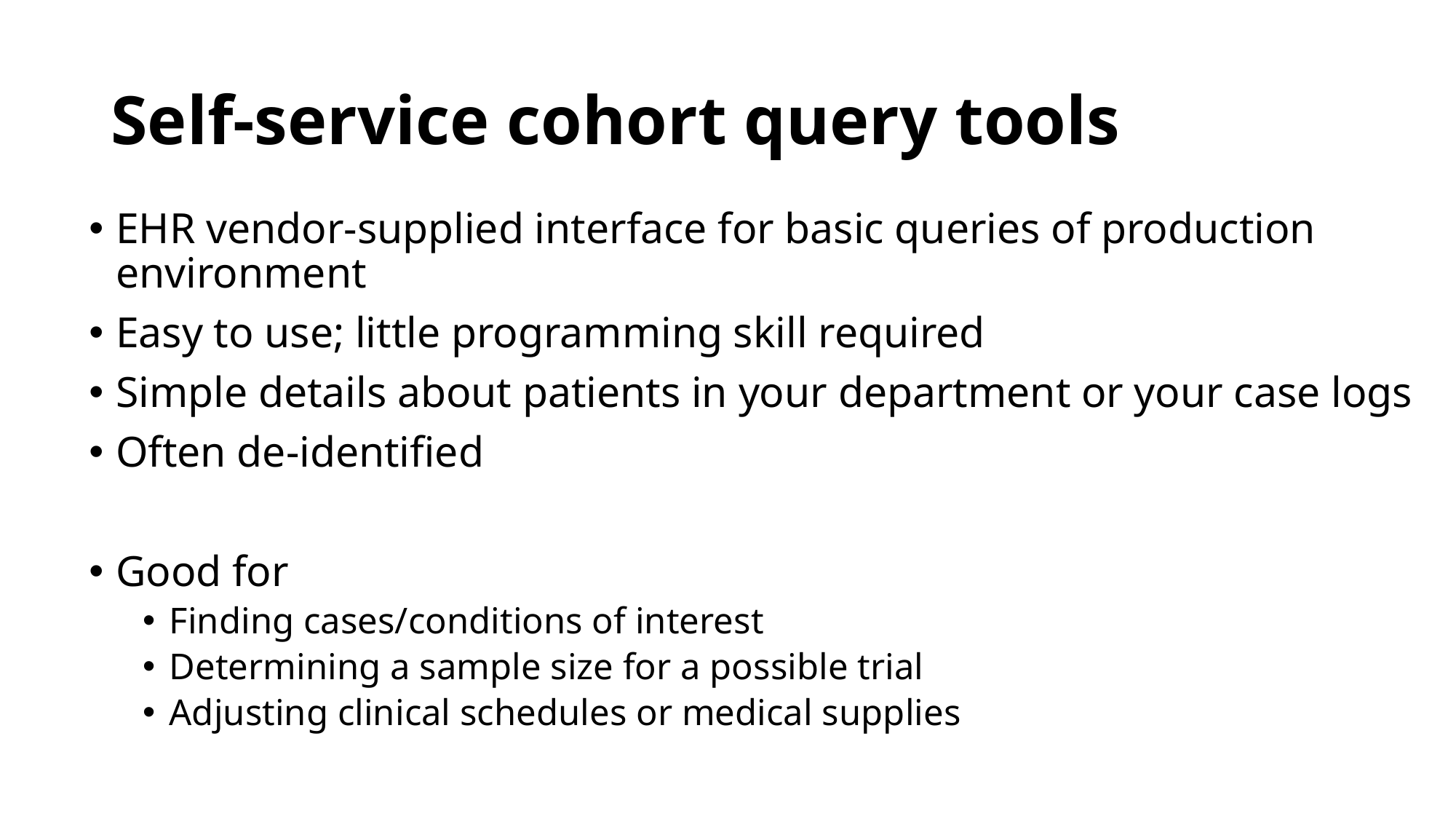

# Self-service cohort query tools
EHR vendor-supplied interface for basic queries of production environment
Easy to use; little programming skill required
Simple details about patients in your department or your case logs
Often de-identified
Good for
Finding cases/conditions of interest
Determining a sample size for a possible trial
Adjusting clinical schedules or medical supplies

## Slide 10
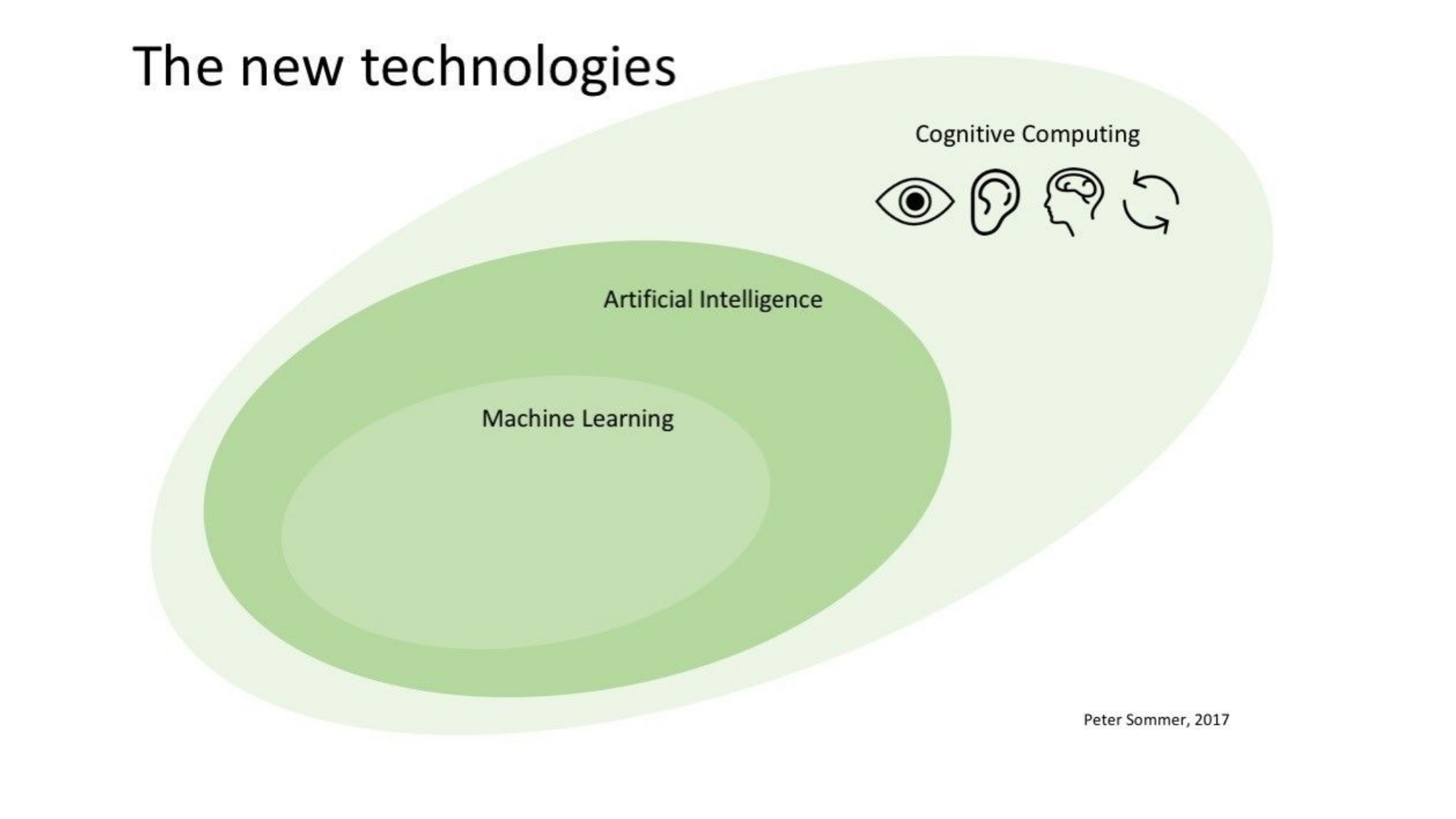

## Slide 11
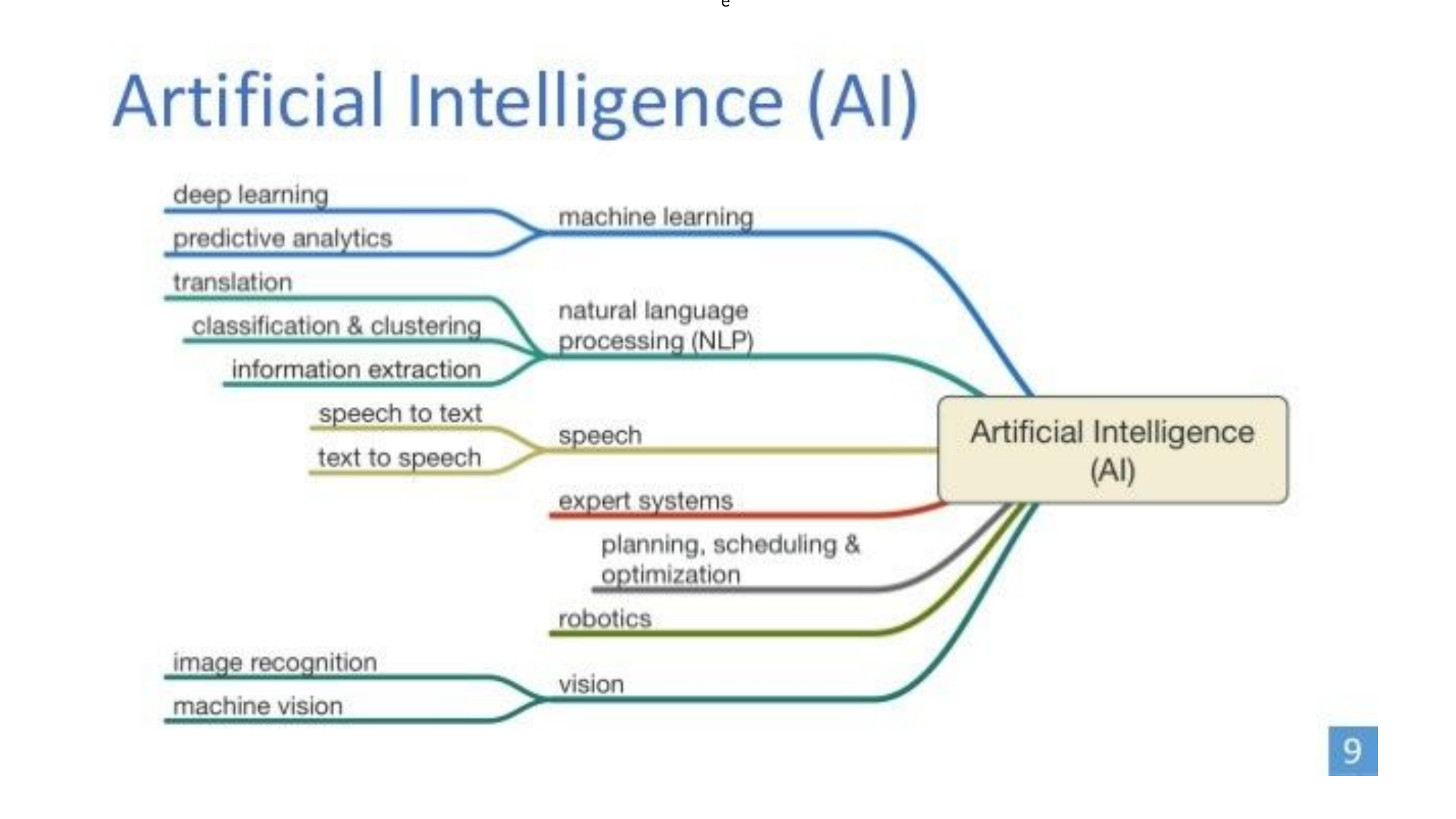

e

## Slide 12
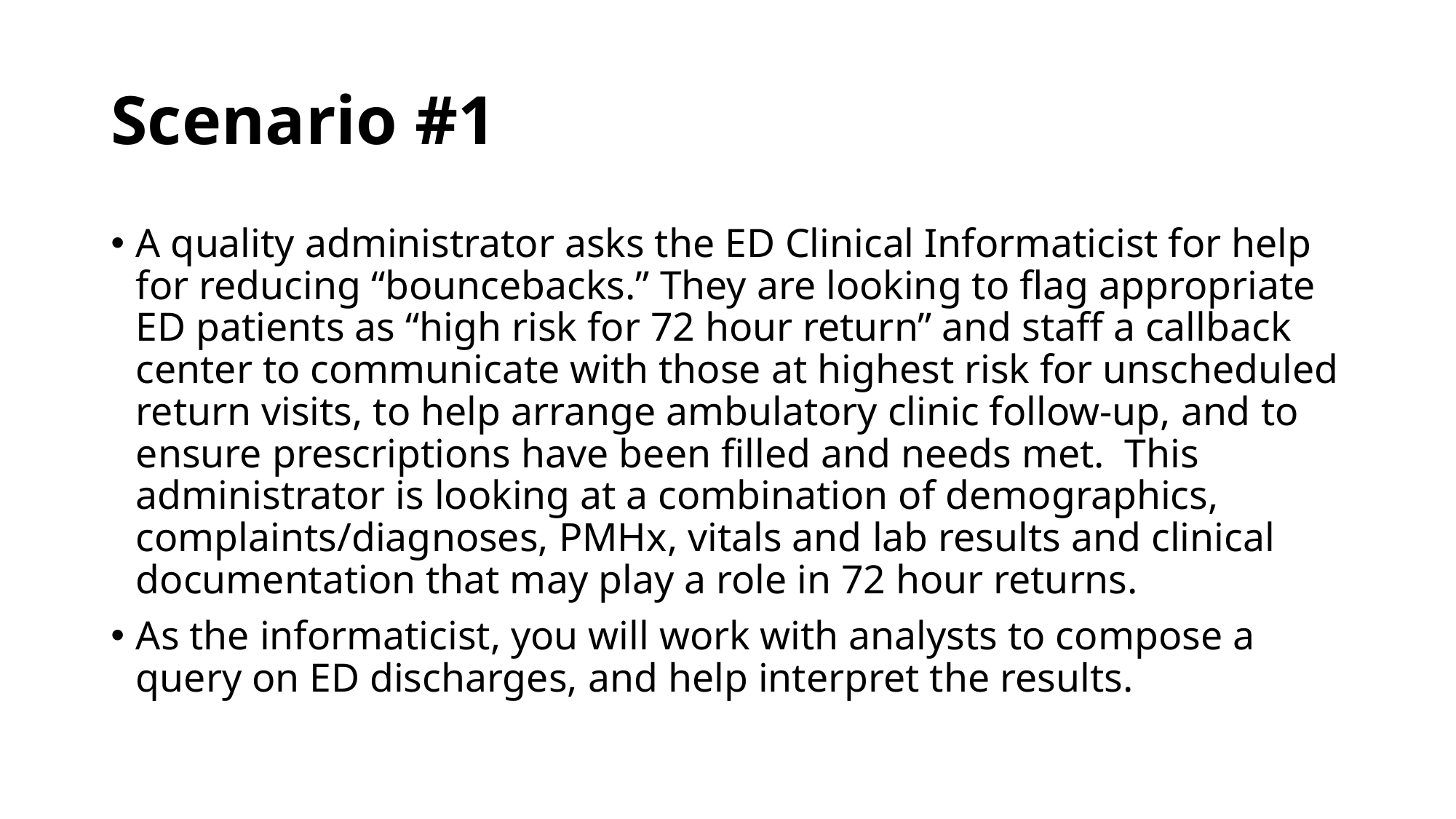

# Scenario #1
A quality administrator asks the ED Clinical Informaticist for help for reducing “bouncebacks.” They are looking to flag appropriate ED patients as “high risk for 72 hour return” and staff a callback center to communicate with those at highest risk for unscheduled return visits, to help arrange ambulatory clinic follow-up, and to ensure prescriptions have been filled and needs met.  This administrator is looking at a combination of demographics, complaints/diagnoses, PMHx, vitals and lab results and clinical documentation that may play a role in 72 hour returns.
As the informaticist, you will work with analysts to compose a query on ED discharges, and help interpret the results.

## Slide 13
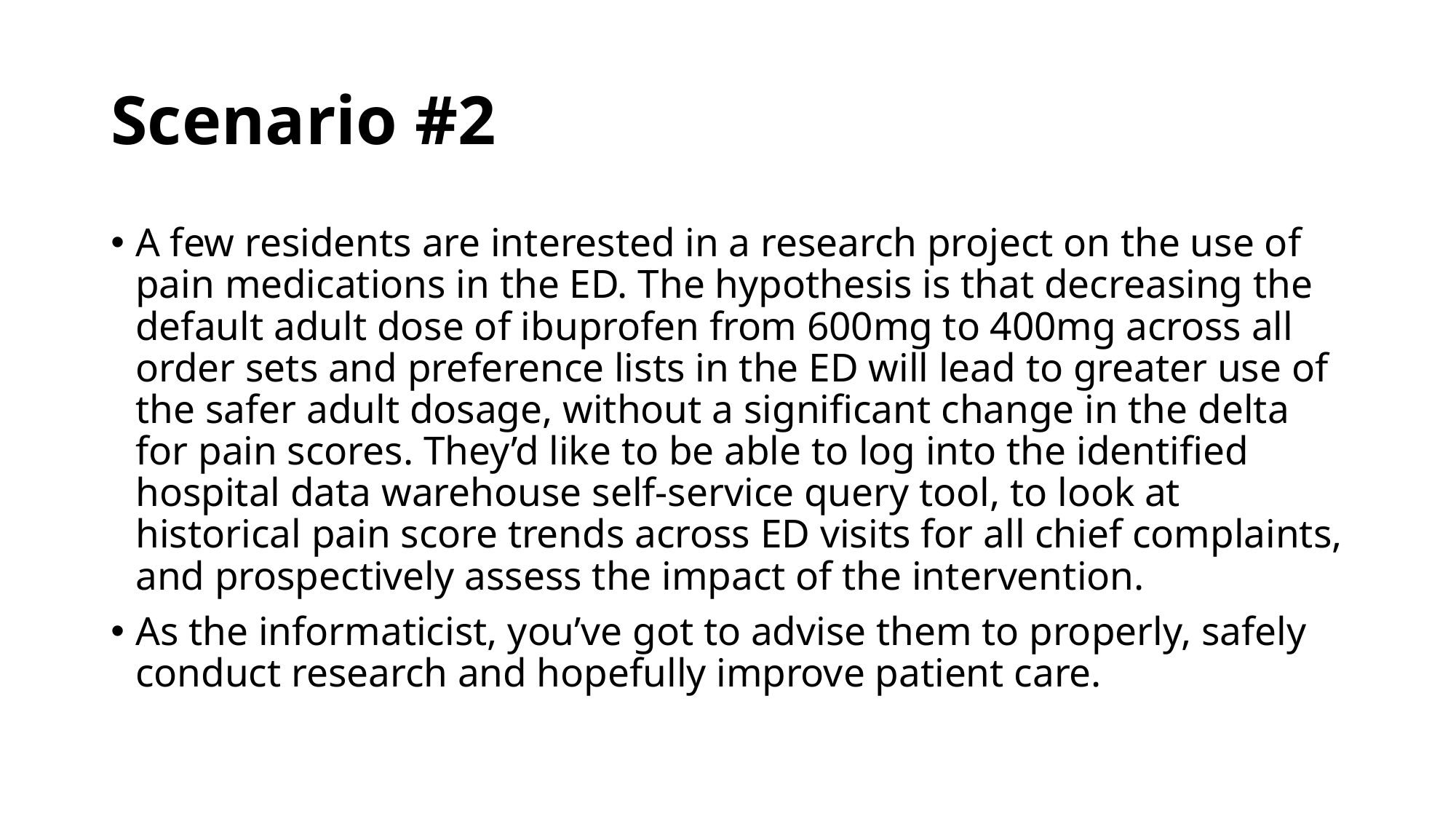

# Scenario #2
A few residents are interested in a research project on the use of pain medications in the ED. The hypothesis is that decreasing the default adult dose of ibuprofen from 600mg to 400mg across all order sets and preference lists in the ED will lead to greater use of the safer adult dosage, without a significant change in the delta for pain scores. They’d like to be able to log into the identified hospital data warehouse self-service query tool, to look at historical pain score trends across ED visits for all chief complaints, and prospectively assess the impact of the intervention.
As the informaticist, you’ve got to advise them to properly, safely conduct research and hopefully improve patient care.

## Slide 14
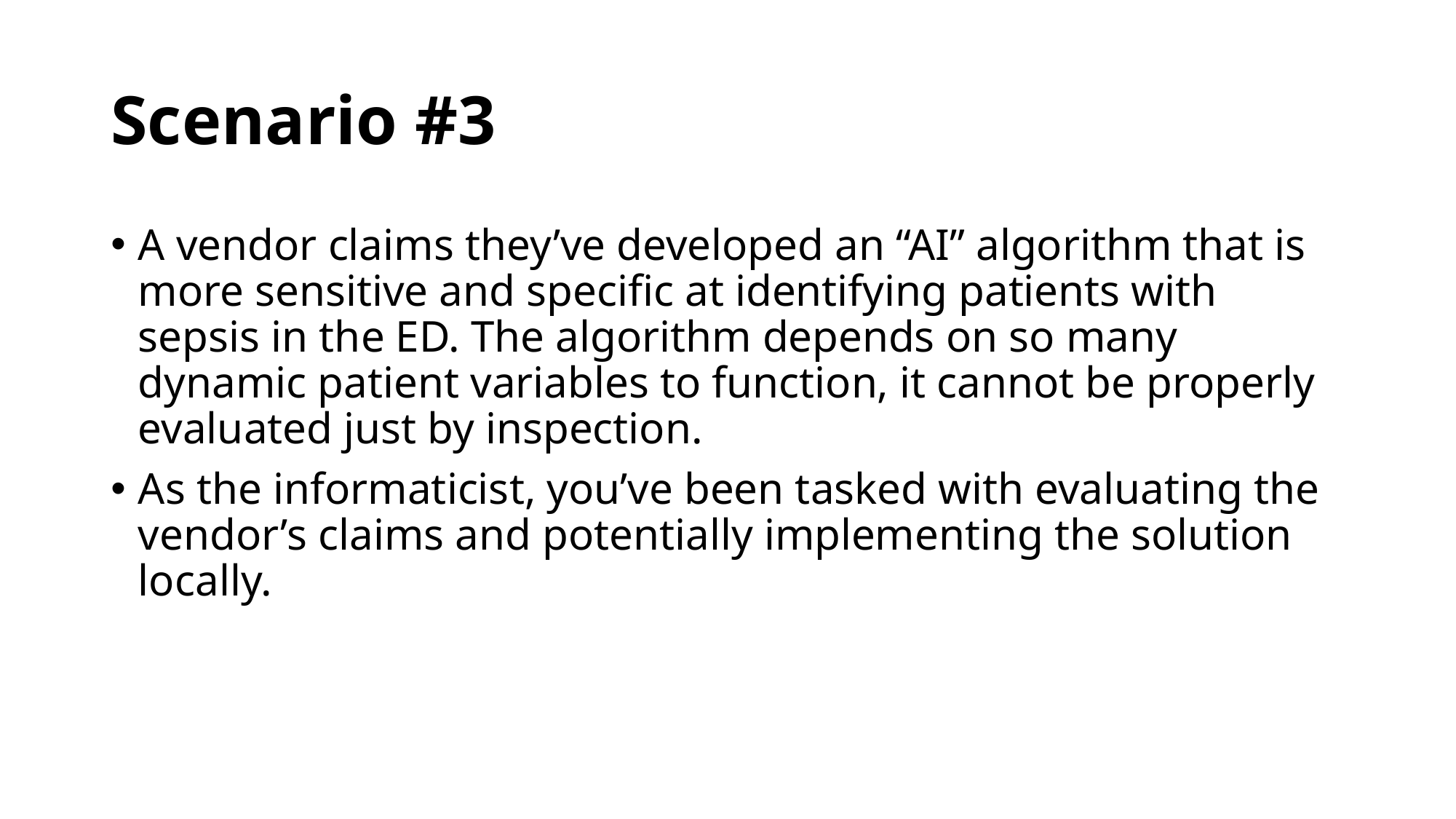

# Scenario #3
A vendor claims they’ve developed an “AI” algorithm that is more sensitive and specific at identifying patients with sepsis in the ED. The algorithm depends on so many dynamic patient variables to function, it cannot be properly evaluated just by inspection.
As the informaticist, you’ve been tasked with evaluating the vendor’s claims and potentially implementing the solution locally.

## Slide 15
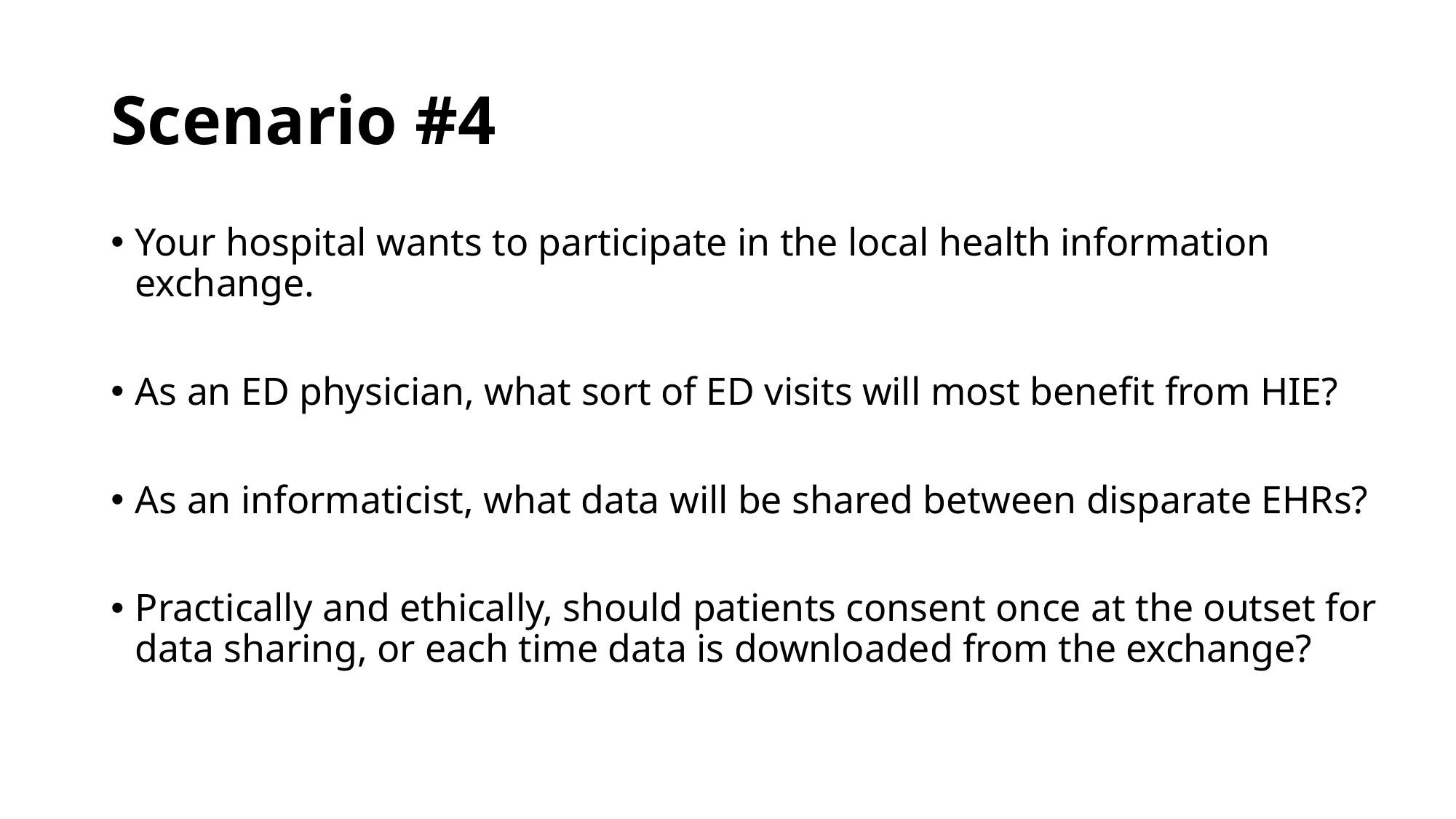

# Scenario #4
Your hospital wants to participate in the local health information exchange.
As an ED physician, what sort of ED visits will most benefit from HIE?
As an informaticist, what data will be shared between disparate EHRs?
Practically and ethically, should patients consent once at the outset for data sharing, or each time data is downloaded from the exchange?

## Slide 16
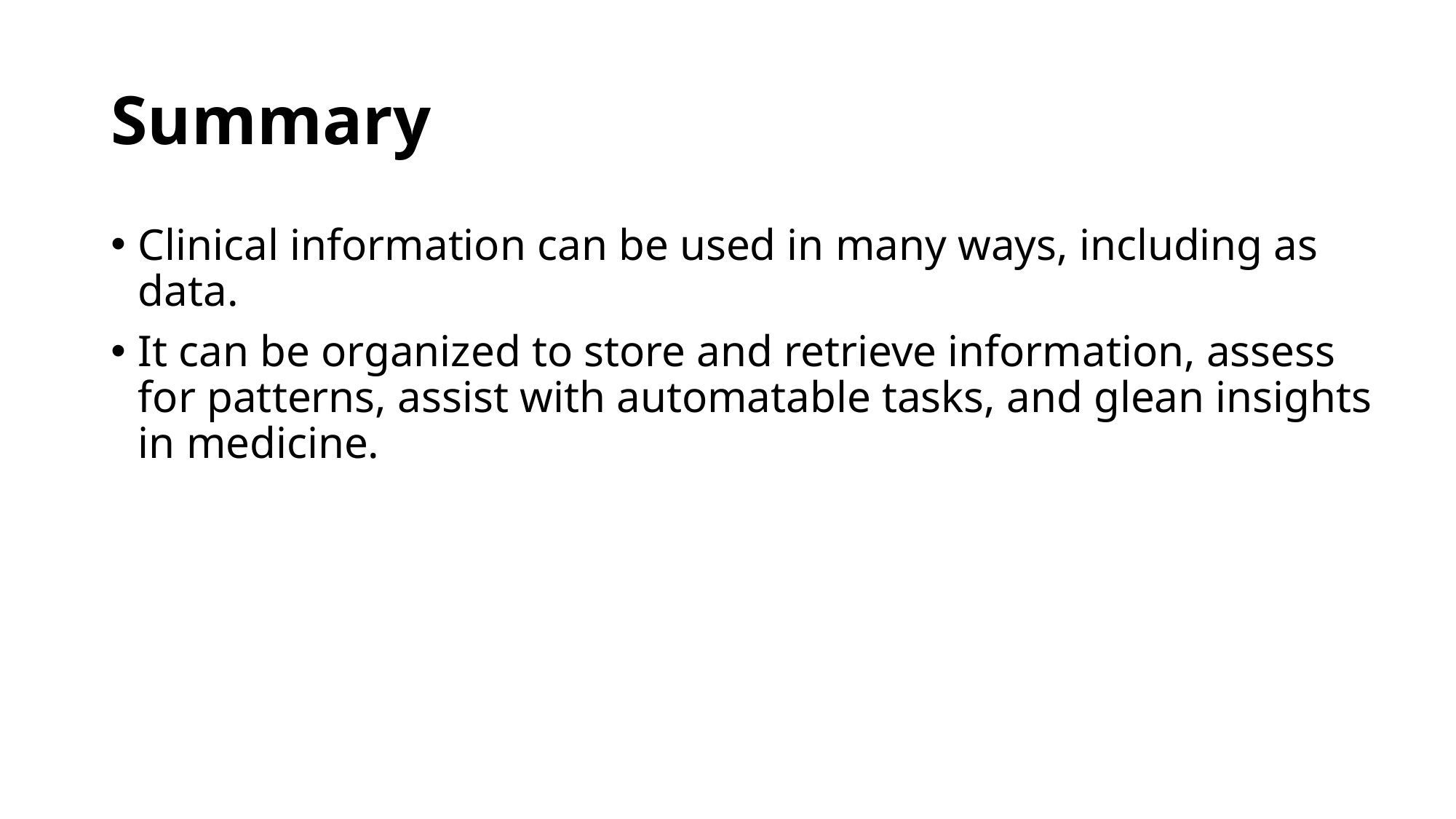

# Summary
Clinical information can be used in many ways, including as data.
It can be organized to store and retrieve information, assess for patterns, assist with automatable tasks, and glean insights in medicine.
