# Supplementary material for: A Model Curriculum for an Emergency Medicine Residency Rotation in Clinical Informatics
Source: J Educ Teach Emerg Med. 2022 Oct 15;7(4):C1–C50. doi: 10.21980/J82P9H (PMC10332664; doi:10.21980/J82P9H)
Supplement: Supplementary file 14 [file JETem-7-4-C1-AppendixE3c.docx]

Appendix E.3.b:

Data Analytics Governance Instructor Material

**Objectives:**

Grounded in examples and discussion, learners will

1. Develop a basic understanding of the concepts of data analytics for ED needs, including techniques of “AI”/machine learning and natural language processing.
2. Understand strategies for data warehouse access and methods of conducting research and quality projects to improve ED care and operations.
3. Appreciate the utility of health information exchange in the ED, as well as some of the challenges of interoperability, necessity of data standards, and tradeoffs involved with different consent models.
4. A quality administrator asks the ED Clinical Informaticist for help for reducing “bouncebacks.” They are looking to flag appropriate ED patients as “high risk for 72 hour return,” and staff a callback center to communicate with those at highest risk for unscheduled return visits, to help arrange ambulatory clinic follow-up, and to ensure prescriptions have been filled and needs met.  This administrator is looking at a combination of demographics, complaints/diagnoses, PMHx (past medical history), vitals and lab results, and clinical documentation that may play a role in 72-hour returns. As the informaticist, you will work with analysts to compose a query on ED discharges and help interpret the results.

1Q1: What challenges do you foresee in collecting and analyzing the discrete data?

1A1: Social Determinants of Health will play a significant factor in 72 hour returns and are not (yet, routinely) captured in EHR data.

1Q2: What approach would you take to analyzing clinical documentation?

1A2: Clinical Documentation is largely unstructured. Natural Language Processing techniques may be suitable for discovering words and phrases associated with higher-risk patients for 72 hour returns.

1Q3: Discuss advantages and disadvantages of methods to identifying future high-risk patients in the ED:

1. Running a daily report of patients that meet predefined high-risk criteria, and sharing it with the callback center.
2. Automatically flagging the charts in the EHR and having the callback center review flagged visits.
3. Asking the clinical staff to manually flag patients they think are at risk for unscheduled returns within 72 hours and having each flag event send a message with patient information to the callback center.

1A3: Method #1 is relatively easy to build and automate and provides the callback center with a daily work list, though it fails to capture any particular patients the ED staff were concerned about.

Method #2 is also relatively easy to build, but requires more work of the callback center.

Method #3 is the most labor-intensive, relying on both human curation of the list and interruptive notifications to the callback center.

1Q4: What challenges do you think the callback center will have, in contacting these patients and preventing unscheduled 72-hour returns? 
1A4: Patients at higher risk for return visits may be the hardest to reach (undomiciled or inconsistent address, no mobile phone). Also, patients today may prefer messaging – either SMS or through the EHR patient portal – to phone calls.

1. A few residents are interested in a research project on the use of pain medications in the ED. The hypothesis is that decreasing the default adult dose of ibuprofen from 600mg to 400mg across all order sets and preference lists in the ED will lead to greater use of the safer adult dosage without a significant change in the delta for pain scores. They’d like to be able to log into the identified hospital data warehouse self-service query tool to look at historical pain score trends across ED visits for all chief complaints and prospectively assess the impact of the intervention.

2Q1: What retrospective data may be possible to analyze first, to help answer the study question, before any prospective changes are made to ibuprofen dosing in the ED preference lists?

2A1: Despite default values, ED providers may choose different initial doses of ibuprofen. So, it might be possible to retrospectively assess the delta for pain scores retrospectively across different initial doses of ibuprofen, if steps are taken to match patient characteristics and diagnoses.

2Q2: What steps should the residents take before conducting this research?

2A2: The residents should demonstrate an understanding of research ethics. They should submit an internal review board application stating the scope of the research and describing the need for their data and their plans to access and secure it. Finally, they should demonstrate proficiency in safely and efficiently running queries using the self-service tools.

2Q3: Name several alternatives to providing the residents with data warehouse access.

2A3: An honest broker can run (or receive) the report for the relevant fields of interest, and de-identify it, giving the residents data they need for the research without risking PHI (protected health information) loss. Or a datamart could be developed, so the residents can’t access data out of scope of their project. The residents could access a de-identified self-service query tool, if available.

1. A vendor claims they’ve developed an “AI” algorithm that is more sensitive and specific at identifying patients with sepsis in the ED. The algorithm depends on so many dynamic patient variables to function, it cannot be properly evaluated just by inspection.

3Q1: Describe several approaches to evaluating the vendor’s claim and potentially partnering with the vendor to improve clinical care.

3A1: Approaches include

- Review the algorithm’s performance on the vendor’s training and evaluation data set. Determine what different characteristics those patients had with your hospitals’ patients, and whether those are likely to be significant.
- Partner with the vendor on researching and developing the sepsis algorithm, tailored to your ED patient population, through use of historical data in the data warehouse.
- Sign a non-disclosure agreement with the vendor, build a secure interface between your institution and the vendor, and test their algorithm on a sandboxed version of your EHR. This EHR can be populated by “synthetic” patients, or de-identified reflections of patients, or if a business agreement is in place, real patients whose demographic data and workup decisions are mirrored from the production environment. These approaches can be time-consuming and expensive, so if the algorithm doesn’t perform well, there will be sunk costs.

3Q2: What are some risks of using AI algorithms for clinical care?

3A2: The biggest risk comes from the algorithm’s complexity masking bias. There are already many examples in healthcare where AI recommendations thought to be based entirely on clinical details were actually found to be based on race, socioeconomic status, or other factors. Because the recommendations often appear as a “black box” to clinicians, some time may pass before these biases can be detected and properly interpreted.

1. Your hospital wants to participate in the local HIE (health information exchange).

4Q1: As an ED physician, do you expect ED patients would benefit from health information exchange?

4A1: Access to HIE has been shown to reduce redundant testing in the ED and reduce costs, and many cases have been described where the HIE provided helpful data for reaching a diagnosis and treatment faster.

4Q2: What ED presentations are least likely to benefit from HIE?

4A2: HIE could potentially make a difference in any ED presentation, if there’s a history of allergy or a potential for drug interactions that would otherwise be missed (particularly if patients are unable to share their allergies or medication lists). However, ED visits concerning minor traumas resulting in sprains, fractures, or lacerations seem less likely to benefit from the data in an HIE than, for example, a patient with recent surgery, or on chemotherapy, or managing complex chronic conditions.

4Q3: The hospitals participating in the HIE are on different EHRs. How will data about patients from different facilities be shared and accessible?

4A3: Most discrete data today – diagnoses, medications, vital signs, lab results, etc – are captured through standard terminologies (like ICD-10, RxNorm, LOINC, etc). Messages between facilities and HIE are also sent according to a common standard. Patients are matched with algorithms that permit some small degree of “fuzziness” so records from two facilities can be recognized as belonging to the same patient so long as there are very closes matches with name, birthday, address, and potentially other criteria. However, idiosyncrasies in how notes are categorized and described, and particular aspects of each EHR, mean that browsing patient data supplied by an HIE is not as seamless as viewing a chart compiled within a single institution.

4Q4: For this new HIE, your hospital wants to require a separate consent for patient data, so that neighboring facilities must document their own consent from the patient to access your hospital’s data. Is this feasible? Why would the hospital pursue this? How does this balance patient privacy vs patient care needs?

4A4: This arrangement is technically possible (attempts at downloading HIE data from your hospital’s institution can be met with a specialized consent form) and may be viewed by the hospital as patient-centric (if they have data at the hospital they’d rather not disclose to outside parties). However, this extra consent introduces an additional step for obtaining access, making routine use of HIE data less likely in the ED, and possibly jeopardizing care if critical details are missed or overlooked (or more difficult to collect, such as if patient arrives unconscious at the other facility).
