# Supplementary material for: A Model Curriculum for an Emergency Medicine Residency Rotation in Clinical Informatics
Source: J Educ Teach Emerg Med. 2022 Oct 15;7(4):C1–C50. doi: 10.21980/J82P9H (PMC10332664; doi:10.21980/J82P9H)
Supplement: Supplementary file 15 [file JETem-7-4-C1-AppendixE3d.docx]

Appendix E.3.c:

Data Analytics Governance Learner Material

**Objectives:**

Grounded in examples and discussion, learners will

1. Develop a basic understanding of the concepts of data analytics for ED needs, including techniques of “AI”/machine learning and natural language processing.
2. Understand strategies for data warehouse access and methods of conducting research and quality projects to improve ED care and operations.
3. Appreciate the utility of health information exchange in the ED, as well as some of the challenges of interoperability, necessity of data standards, and tradeoffs involved with different consent models.
4. A quality administrator asks the ED Clinical Informaticist for help for reducing “bouncebacks.” They are looking to flag appropriate ED patients as “high risk for 72 hour return,” and staff a callback center to communicate with those at highest risk for unscheduled return visits, to help arrange ambulatory clinic follow-up, and to ensure prescriptions have been filled and needs met.  This administrator is looking at a combination of demographics, complaints/diagnoses, PMHx (past medical history), vitals and lab results, and clinical documentation that may play a role in 72-hour returns. As the informaticist, you will work with analysts to compose a query on ED discharges and help interpret the results.

1Q1: What challenges do you foresee in collecting and analyzing the discrete data?

1Q2: What approach would you take to analyzing clinical documentation?

1Q3: Discuss advantages and disadvantages of methods to identifying future high-risk patients in the ED:

1. Running a daily report of patients that meet predefined high-risk criteria, and sharing it with the callback center.
2. Automatically flagging the charts in the EHR and having the callback center review flagged visits.
3. Asking the clinical staff to manually flag patients they think are at risk for unscheduled returns within 72 hours and having each flag event send a message with patient information to the callback center.

1Q4: What challenges do you think the callback center will have, in contacting these patients and preventing unscheduled 72-hour returns?

1. A few residents are interested in a research project on the use of pain medications in the ED. The hypothesis is that decreasing the default adult dose of ibuprofen from 600mg to 400mg across all order sets and preference lists in the ED will lead to greater use of the safer adult dosage without a significant change in the delta for pain scores. They’d like to be able to log into the identified hospital data warehouse self-service query tool to look at historical pain score trends across ED visits for all chief complaints and prospectively assess the impact of the intervention.

2Q1: What retrospective data may be possible to analyze first, to help answer the study question, before any prospective changes are made to ibuprofen dosing in the ED preference lists?

2Q2: What steps should the residents take before conducting this research?

2Q3: Name several alternatives to providing the residents with data warehouse access.

1. A vendor claims they’ve developed an “AI” algorithm that is more sensitive and specific at identifying patients with sepsis in the ED. The algorithm depends on so many dynamic patient variables to function, it cannot be properly evaluated just by inspection.

3Q1: Describe several approaches to evaluating the vendor’s claim and potentially partnering with the vendor to improve clinical care.

3Q2: What are some risks of using AI algorithms for clinical care?

1. Your hospital wants to participate in the local HIE (health information exchange).

4Q1: As an ED physician, do you expect ED patients would benefit from health information exchange?

4Q2: What ED presentations are least likely to benefit from HIE?

4Q3: The hospitals participating in the HIE are on different EHRs. How will data about patients from different facilities be shared and accessible?

4Q4: For this new HIE, your hospital wants to require a separate consent for patient data, so that neighboring facilities must document their own consent from the patient to access your hospital’s data. Is this feasible? Why would the hospital pursue this? How does this balance patient privacy vs patient care needs?
