# Supplementary material for: A Model Curriculum for an Emergency Medicine Residency Rotation in Clinical Informatics
Source: J Educ Teach Emerg Med. 2022 Oct 15;7(4):C1–C50. doi: 10.21980/J82P9H (PMC10332664; doi:10.21980/J82P9H)
Supplement: Supplementary file 16 [file JETem-7-4-C1-AppendixE4a.docx]

Appendix E.4:

Small Group Discussion: Leadership and Professionalism

**Pre-Session Preparation:**

This small group session is to be held during the fourth week of the rotation. See the proposed calendar for details. Faculty and learners will have completed pre-readings during their asynchronous learning sessions.

Instructor preparation: 45 minutes, Learner Responsible Content: 30 minutes, In Class Exercise: 90 minutes

**Recommended Pre-Reading:**

1. Schleyer T, Zappone S, Wells-Meyers C, Saxton T. Effective Interdisciplinary Teams. In. Finnell JT, Dixon BE, eds. *Clinical Informatics Study Guide.* 2nd ed. Springer; 2022: 285-306.
2. Sivers D. How to start a movement. Ted.com. Feb 2010. Accessed April 12, 2022. https://www.ted.com/talks/derek_sivers_how_to_start_a_movement?language=en#t-169239
3. Varkey P, Reller MK, Resar RK. Basics of quality improvement in health care. *Mayo Clin Proc*. 2007 Jun;82(6):735-9.
4. McMillan SS, King M, Tully MP. How to use the nominal group and Delphi techniques. *Int J Clin Pharm*. 2016;38(3):655-662.

**Objectives:**

Residents will learn leadership skills that can be applied to improve patient care.

1. Utilize informatics techniques to perform research and quality improvement projects.
2. Develop and present a realistic informatics-based project plan for a problem in their clinical environment with the option to pursue further research or quality improvement.
3. Appreciate the role and need for project management, change management, and stakeholder engagement.
4. Learn the basics of team management and effective communication skills including developing a team charter, creating a meeting agenda, identifying stakeholders, and making group decisions using nominal group techniques.
5. Appreciate the role EM physicians can play in departmental and hospital leadership.

**Linked objectives and methods:**

Objectives are achieved through small group discussion with guidance from the small group instructor. The faculty will use the provided PowerPoint, “Appendix E.4.a. Leadership PPT,” to guide the group discussion. An electronic or printed document, “Appendix E.4.b. Leadership Form,” will be given to each participant to use during the small group the session.

Each person will have the opportunity to learn through listening, reflecting, committing to a decision, brainstorming ideas, discussion, and voting. This allows for knowledge translation in an informal setting. Learners discuss their experiences and ideas in an open format.
