# Supplementary material for: A Model Curriculum for an Emergency Medicine Residency Rotation in Clinical Informatics
Source: J Educ Teach Emerg Med. 2022 Oct 15;7(4):C1–C50. doi: 10.21980/J82P9H (PMC10332664; doi:10.21980/J82P9H)
Supplement: Supplementary file 17 — Please see associated PowerPoint file [file JETem-7-4-C1-AppendixE4b.pptx]

## Slide 1
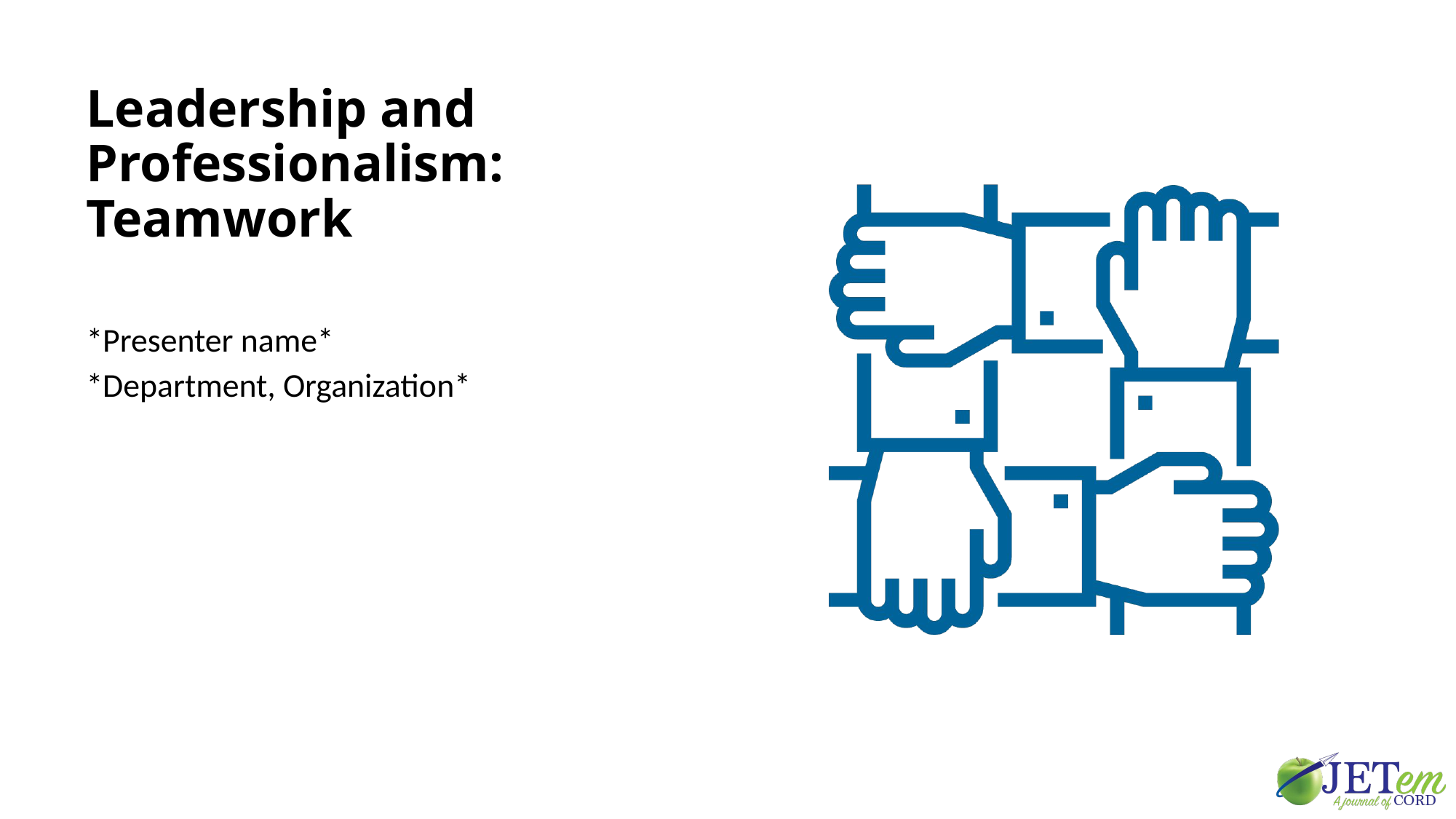

# Leadership and Professionalism: Teamwork​
*Presenter name*
*Department, Organization*

## Slide 2
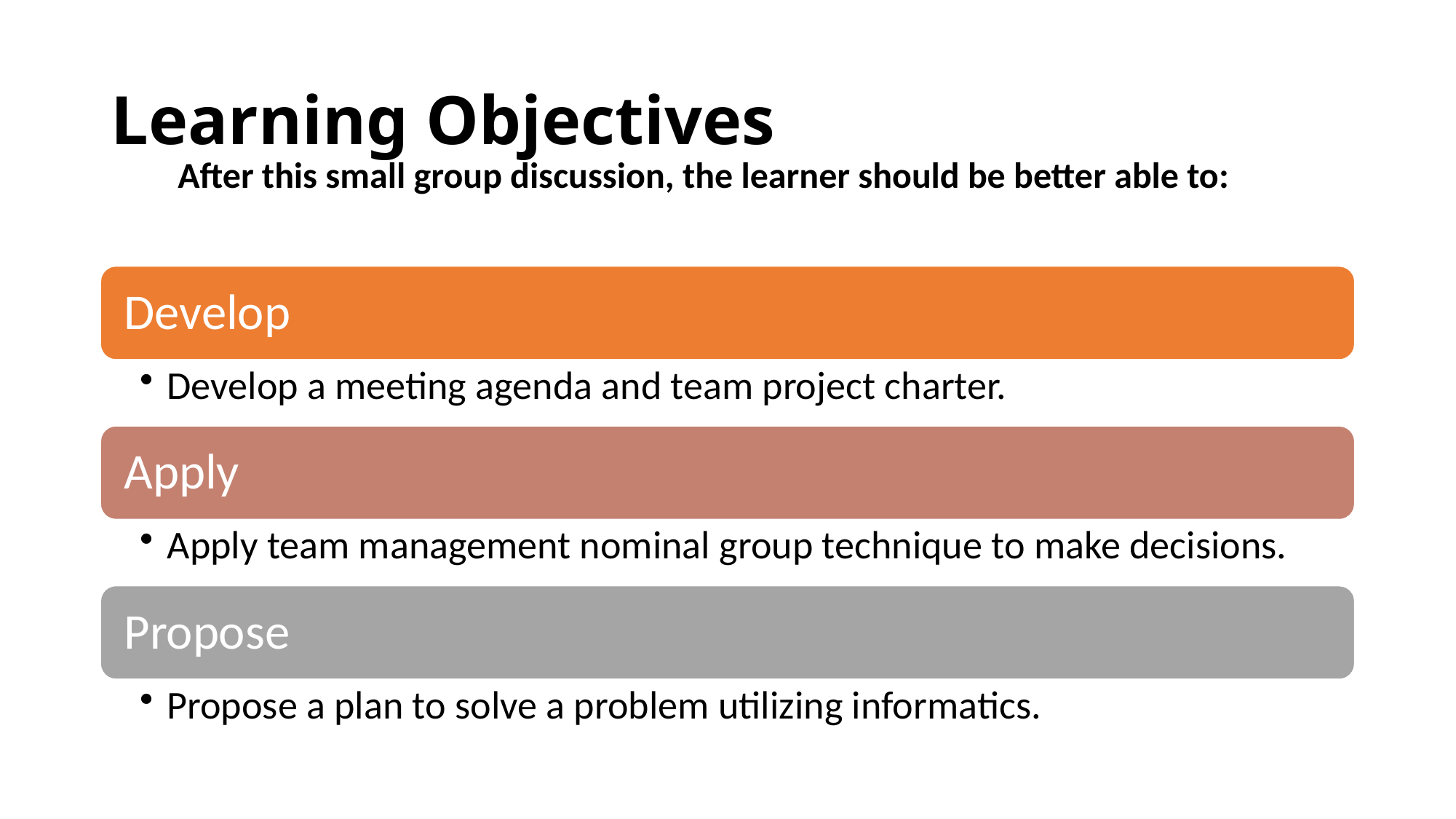

# Learning Objectives​
After this small group discussion, the learner should be better able to:​

## Slide 3
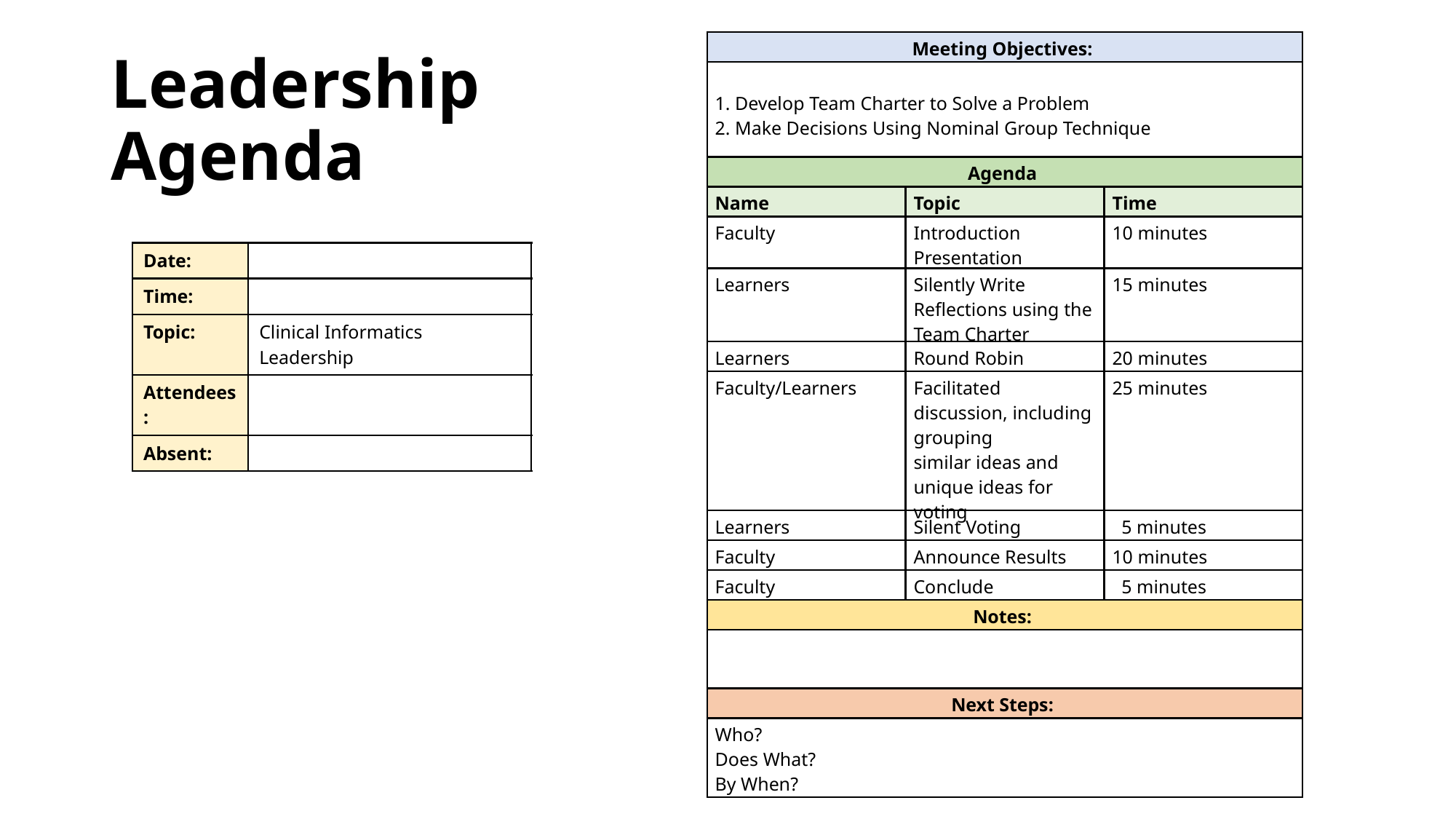

| Meeting Objectives: ​ | | |
| --- | --- | --- |
| ​ 1. Develop Team Charter to Solve a Problem ​ 2. Make Decisions Using Nominal Group Technique ​  ​ | | |
| Agenda ​ | | |
| Name ​ | Topic ​ | Time ​ |
| Faculty ​ | Introduction Presentation ​ | 10 minutes ​ |
| Learners ​ | Silently Write Reflections using the Team Charter ​ | 15 minutes ​ |
| Learners ​ | Round Robin ​ | 20 minutes ​ |
| Faculty/Learners ​ | Facilitated discussion, including grouping similar ideas and unique ideas for voting ​ | 25 minutes ​ |
| Learners ​ | Silent Voting ​ | 5 minutes ​ |
| Faculty ​ | Announce Results ​ | 10 minutes ​ |
| Faculty ​ | Conclude ​ | 5 minutes ​ |
| Notes: ​ | | |
| ​ | | |
| Next Steps: ​ | | |
| Who? Does What? By When? | | |
# Leadership Agenda​
| Date: ​ | ​ |
| --- | --- |
| Time: ​ | ​ |
| Topic: ​ | Clinical Informatics Leadership ​ |
| Attendees: ​ | ​ |
| Absent: ​ | ​ |

## Slide 4
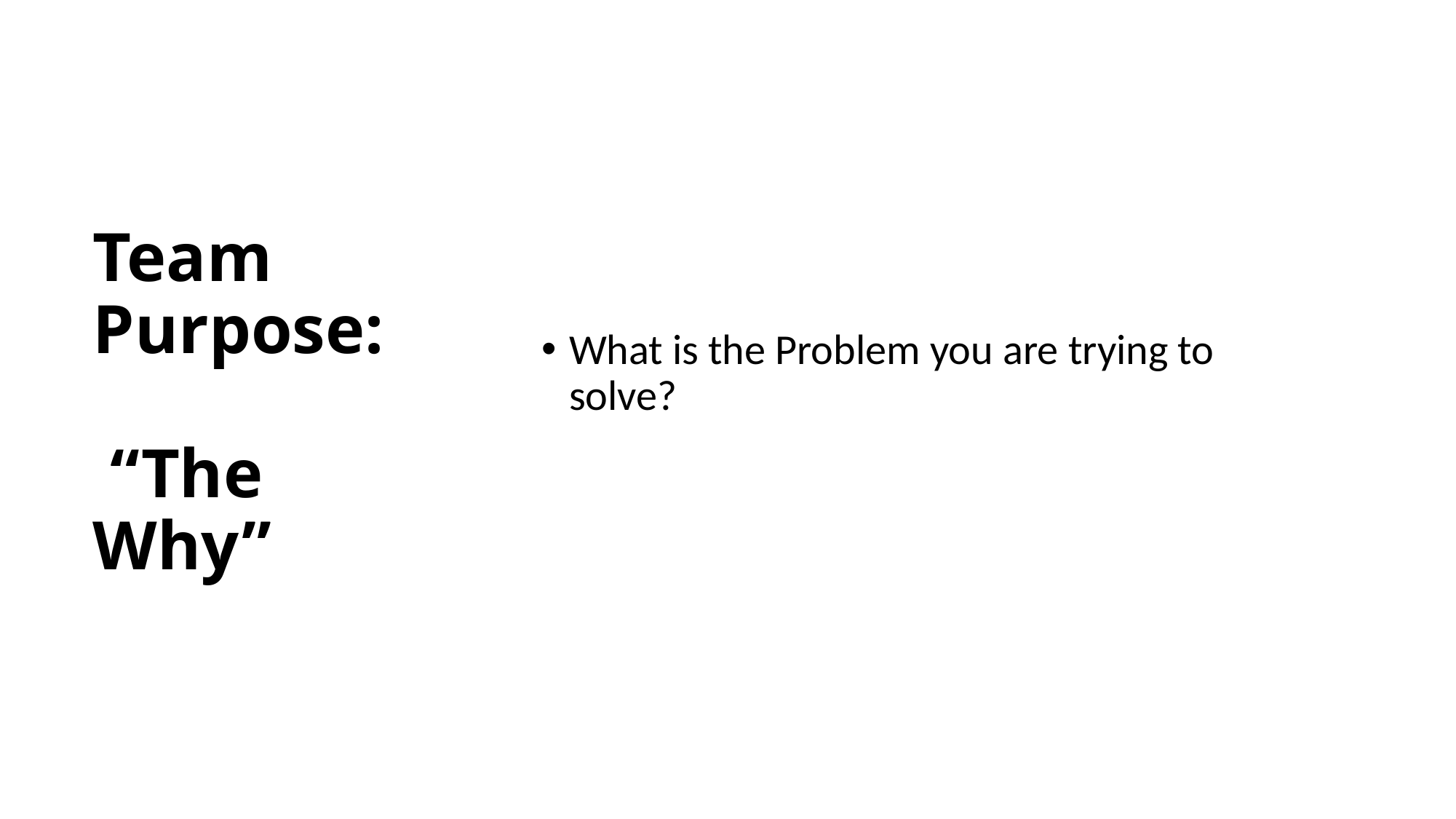

What is the Problem you are trying to solve?  ​
# Team Purpose: “The Why”​

## Slide 5
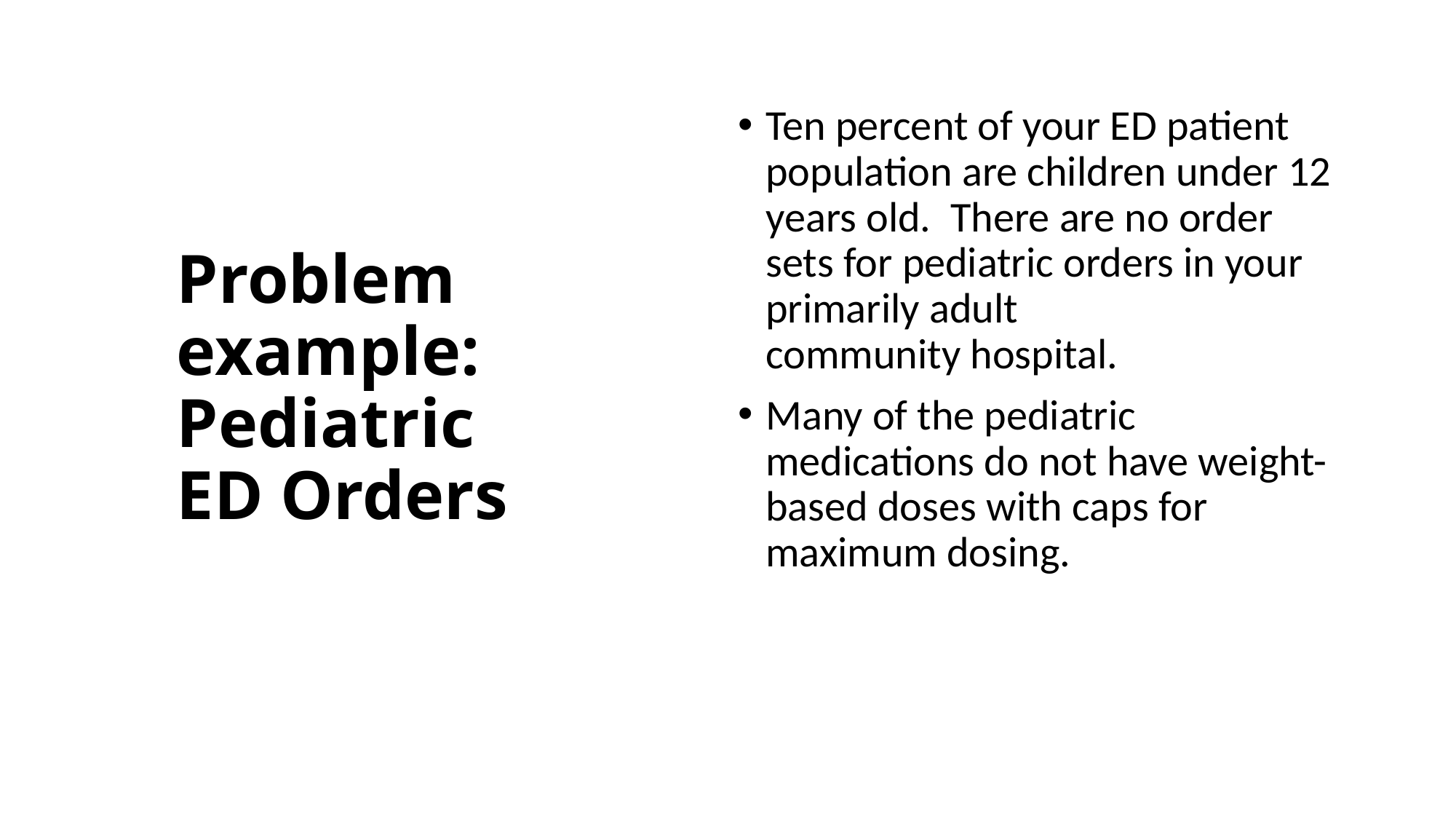

Ten percent of your ED patient population are children under 12 years old.  There are no order sets for pediatric orders in your primarily adult community hospital.
Many of the pediatric medications do not have weight-based doses with caps for maximum dosing.​
# Problem example: Pediatric ED Orders​

## Slide 6
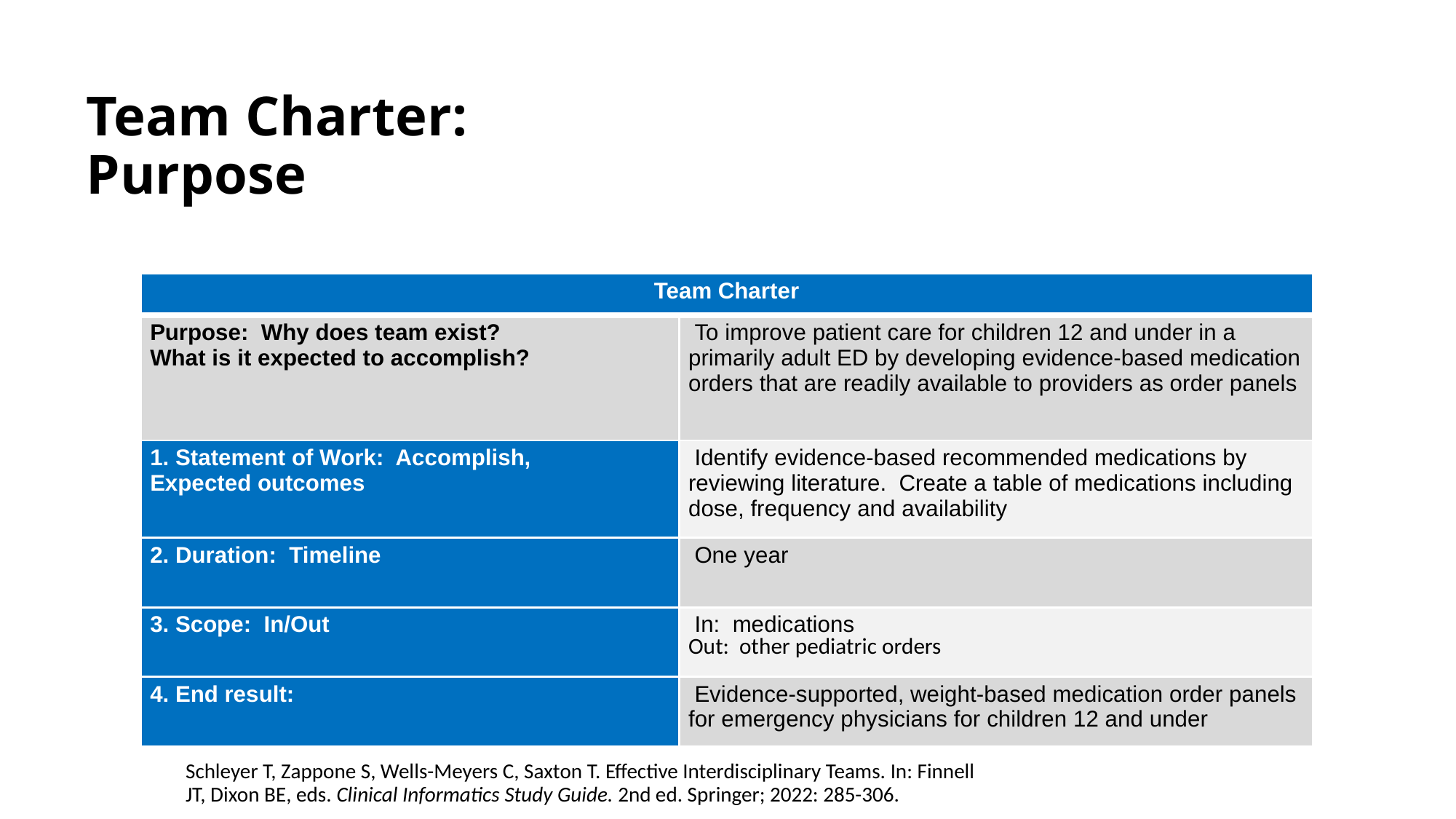

# Team Charter: Purpose​
| Team Charter​ | |
| --- | --- |
| Purpose:  Why does team exist? ​ What is it expected to accomplish?​  ​ | To improve patient care for children 12 and under in a primarily adult ED by developing evidence-based medication orders that are readily available to providers as order panels​ |
| 1. Statement of Work:  Accomplish, Expected outcomes​  ​ | Identify evidence-based recommended medications by reviewing literature.  Create a table of medications including dose, frequency and availability​ |
| 2. Duration:  Timeline​  ​ | One year​ |
| 3. Scope:  In/Out​  ​ | In:  medications​ Out:  other pediatric orders​ |
| 4. End result: ​  ​ | Evidence-supported, weight-based medication order panels for emergency physicians for children 12 and under​ |
Schleyer T, Zappone S, Wells-Meyers C, Saxton T. Effective Interdisciplinary Teams. In: Finnell JT, Dixon BE, eds. Clinical Informatics Study Guide. 2nd ed. Springer; 2022: 285-306.
This Photo by Unknown Author is licensed under CC BY

## Slide 7
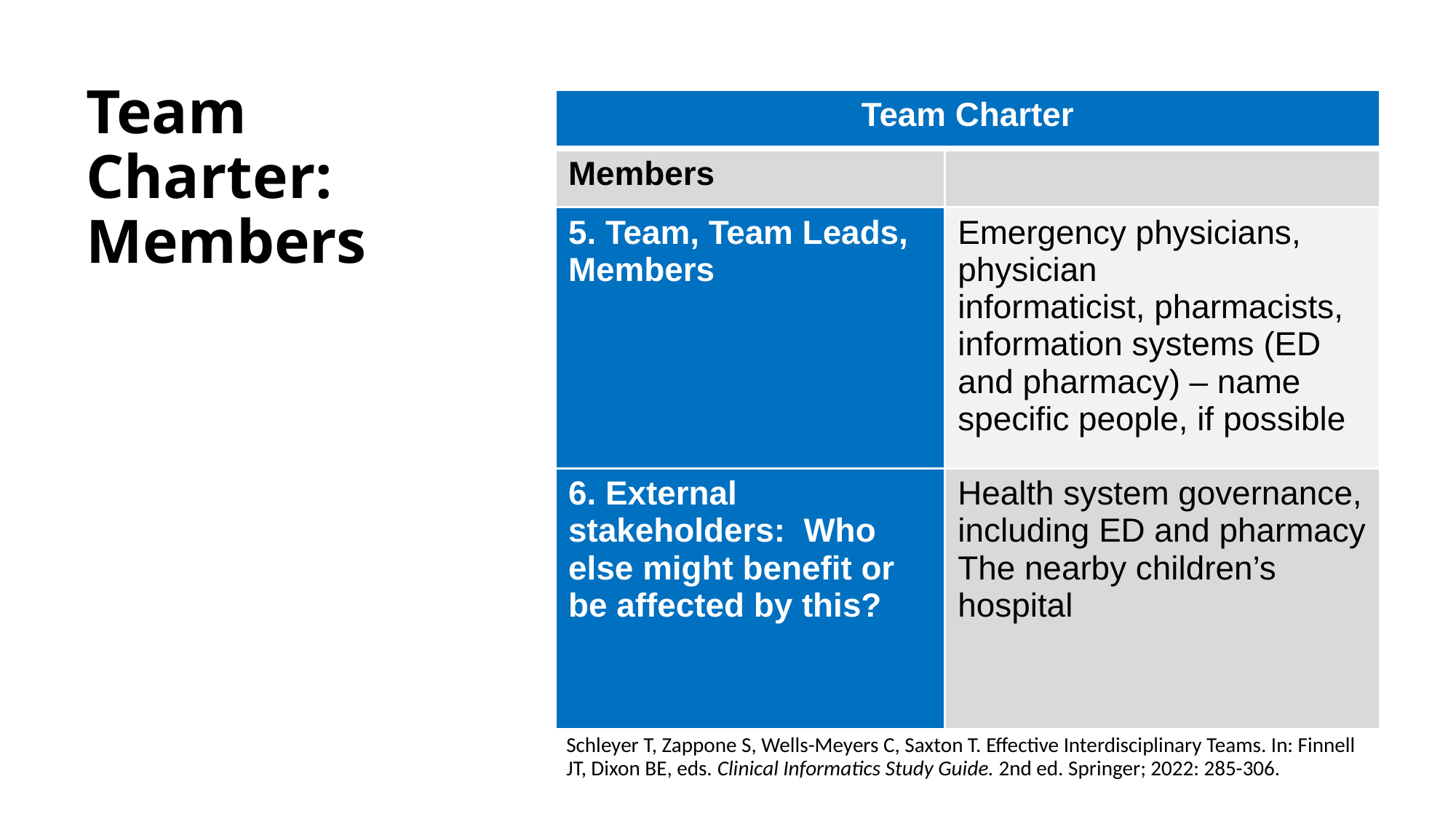

# Team Charter​: Members
| Team Charter​ | |
| --- | --- |
| Members​ | ​ |
| 5. Team, Team Leads, Members​  ​ | Emergency physicians, physician informaticist, pharmacists, information systems (ED and pharmacy) – name specific people, if possible​ |
| 6. External stakeholders:  Who else might benefit or be affected by this?​  ​ | Health system governance, including ED and pharmacy​ The nearby children’s hospital​ ​ |
Schleyer T, Zappone S, Wells-Meyers C, Saxton T. Effective Interdisciplinary Teams. In: Finnell JT, Dixon BE, eds. Clinical Informatics Study Guide. 2nd ed. Springer; 2022: 285-306.

## Slide 8
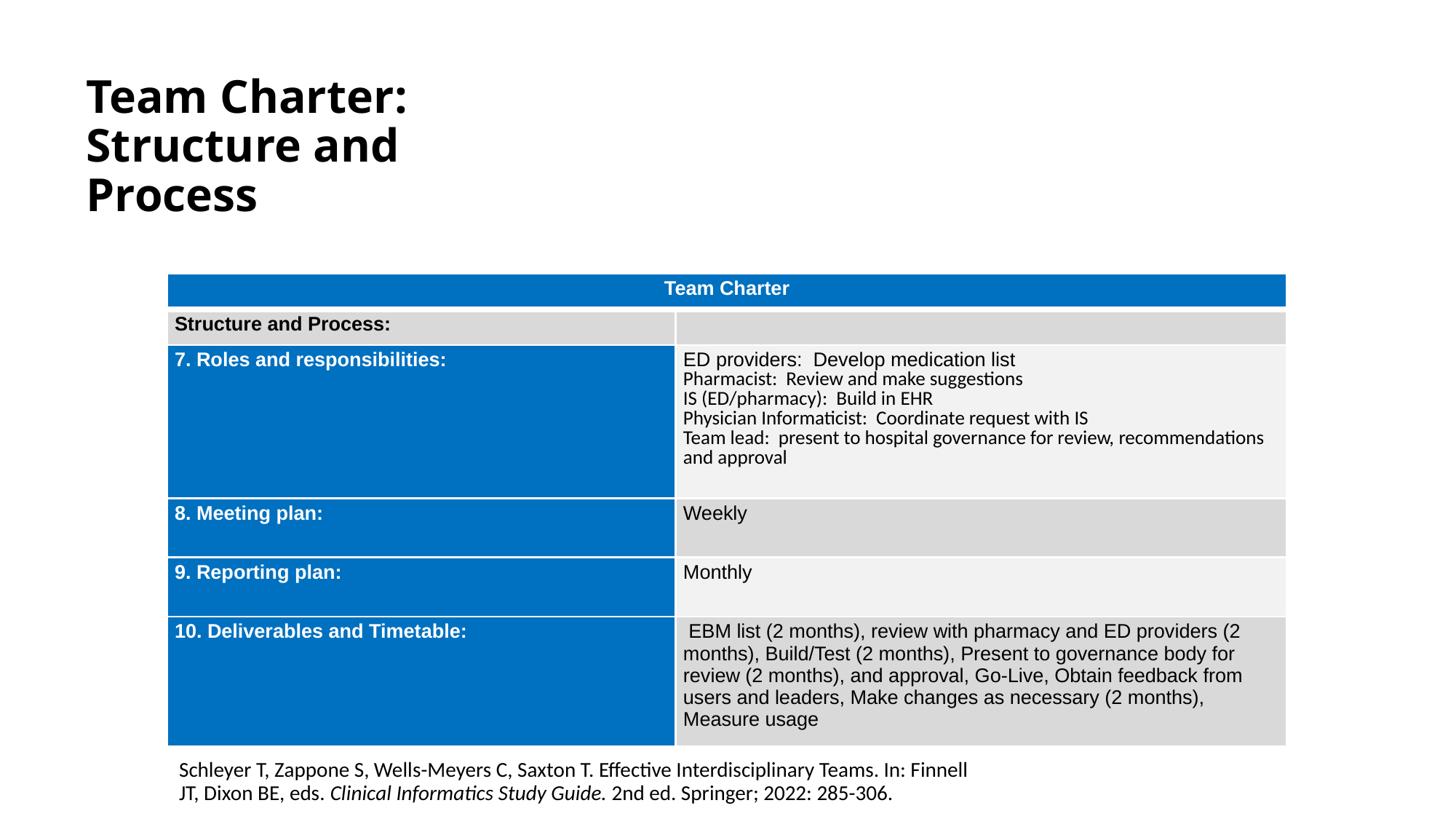

# Team Charter​: Structure and Process
| Team Charter​ | |
| --- | --- |
| Structure and Process:​ | ​ |
| 7. Roles and responsibilities:​  ​ | ED providers:  Develop medication list​ Pharmacist:  Review and make suggestions​ IS (ED/pharmacy):  Build in EHR ​ Physician Informaticist:  Coordinate request with IS​ Team lead:  present to hospital governance for review, recommendations and approval​ |
| 8. Meeting plan:​  ​ | Weekly​ |
| 9. Reporting plan:​  ​ | Monthly​ |
| 10. Deliverables and Timetable:​  ​ | EBM list (2 months), review with pharmacy and ED providers (2 months), Build/Test (2 months), Present to governance body for review (2 months), and approval, Go-Live, Obtain feedback from users and leaders, Make changes as necessary (2 months), Measure usage​ |
Schleyer T, Zappone S, Wells-Meyers C, Saxton T. Effective Interdisciplinary Teams. In: Finnell JT, Dixon BE, eds. Clinical Informatics Study Guide. 2nd ed. Springer; 2022: 285-306.

## Slide 9
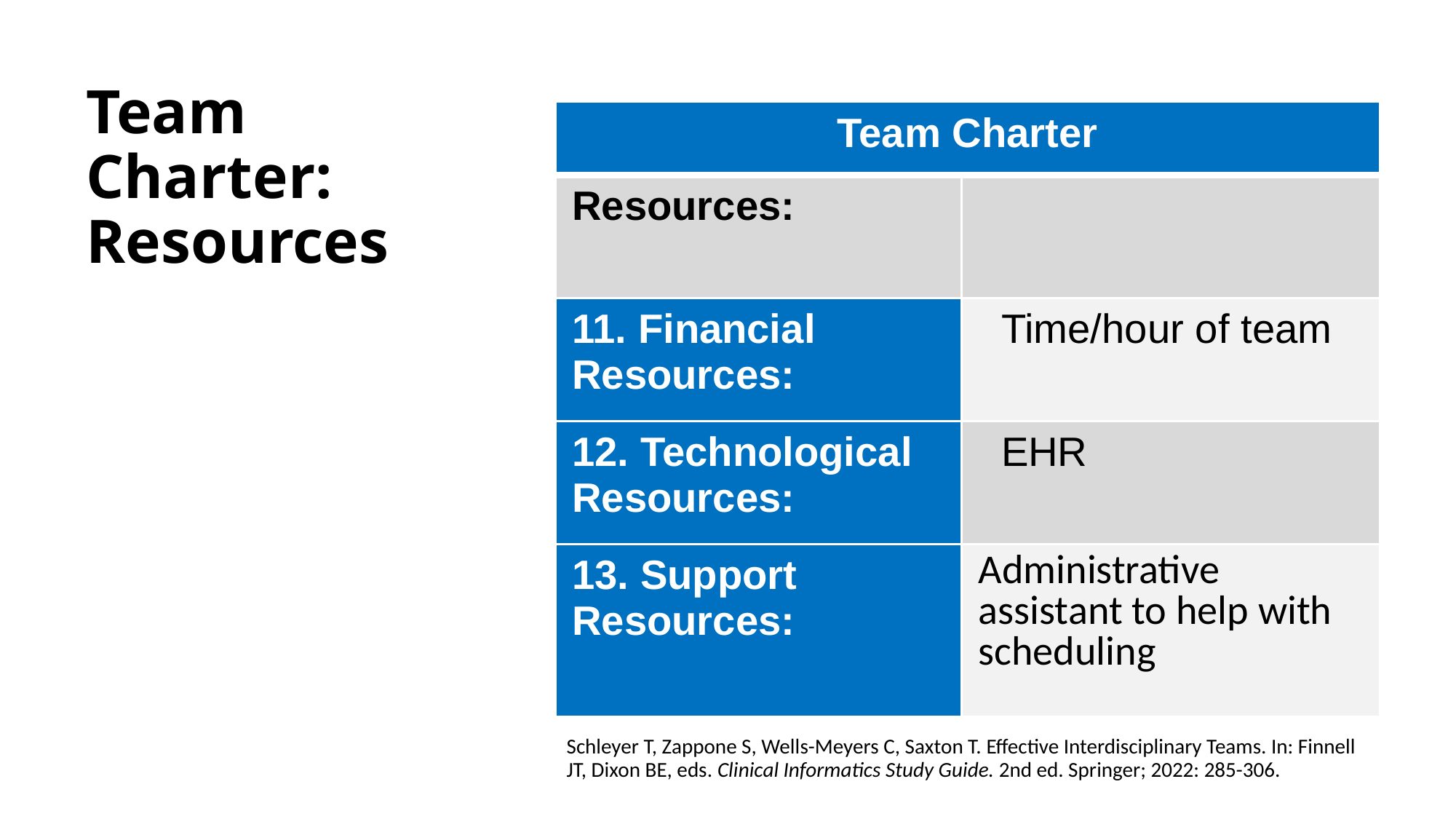

# Team Charter​: Resources
| Team Charter​ | |
| --- | --- |
| Resources:​  ​ | ​ |
| 11. Financial Resources:​ | Time/hour of team​ ​ |
| 12. Technological Resources:​ | EHR​ ​ |
| 13. Support Resources:​  ​ | Administrative assistant to help with scheduling​ |
Schleyer T, Zappone S, Wells-Meyers C, Saxton T. Effective Interdisciplinary Teams. In: Finnell JT, Dixon BE, eds. Clinical Informatics Study Guide. 2nd ed. Springer; 2022: 285-306.

## Slide 10
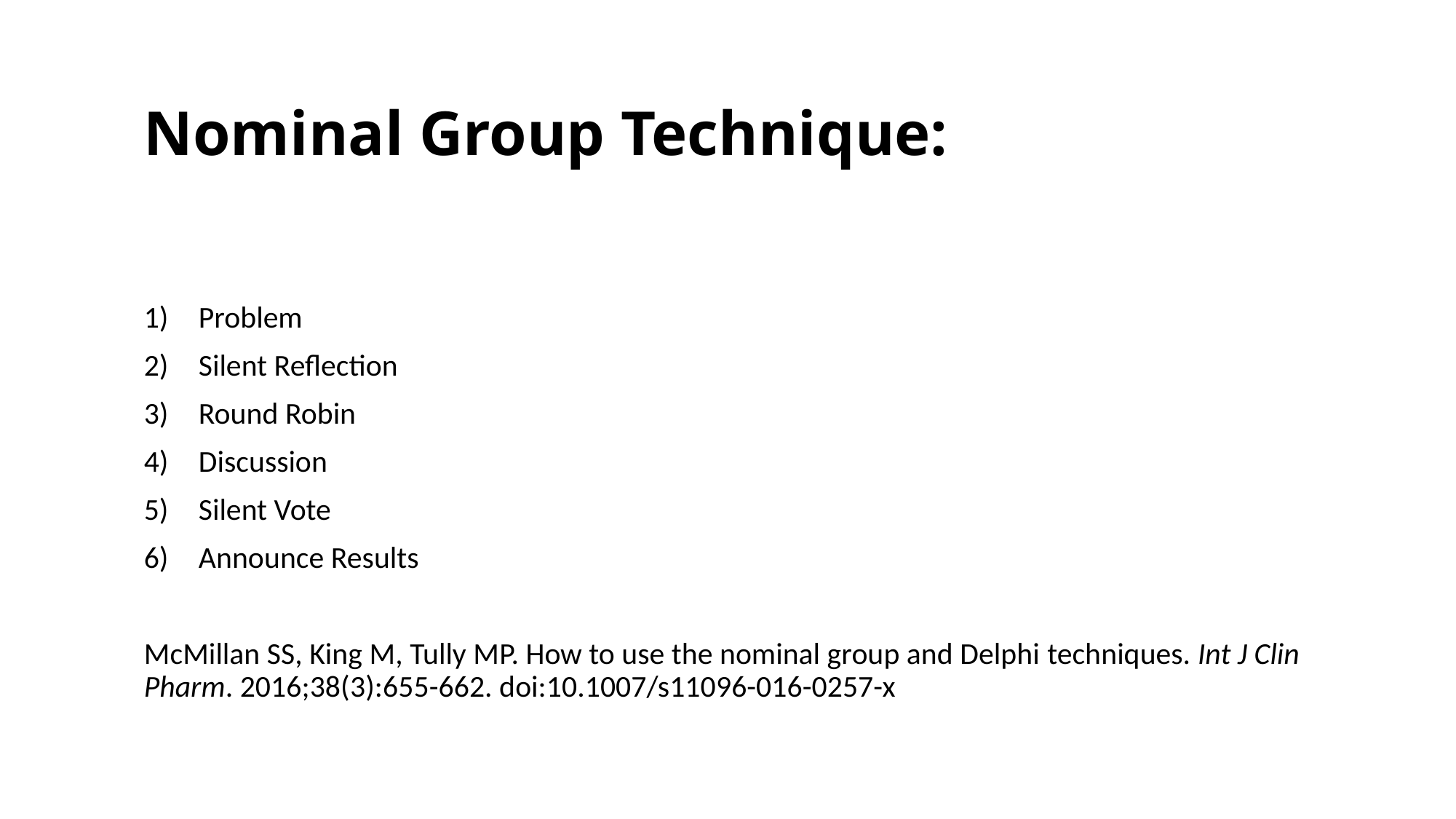

# Nominal Group Technique:
Problem​
Silent Reflection​
Round Robin​
Discussion​
Silent Vote​
Announce Results​
McMillan SS, King M, Tully MP. How to use the nominal group and Delphi ​techniques. Int J Clin Pharm. 2016;38(3):655-662. doi:10.1007/s11096-016-0257-x​

## Slide 11
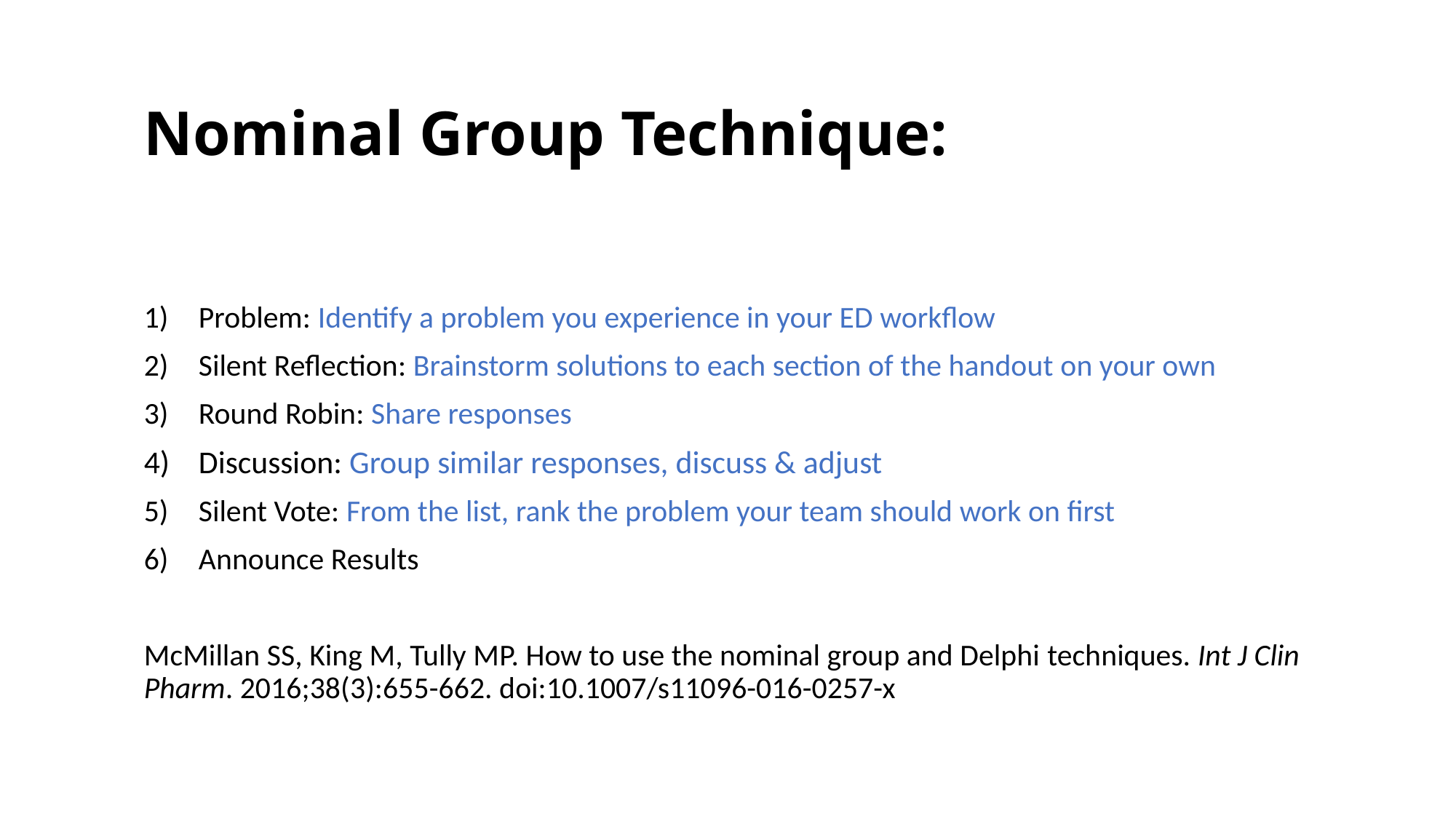

# Nominal Group Technique:
Problem​: Identify a problem you experience in your ED workflow
Silent Reflection​: Brainstorm solutions to each section of the handout on your own​
Round Robin​: Share responses
Discussion​: Group similar responses, discuss & adjust
Silent Vote​: From the list, rank the problem your team should work on first
Announce Results​
McMillan SS, King M, Tully MP. How to use the nominal group and Delphi ​techniques. Int J Clin Pharm. 2016;38(3):655-662. doi:10.1007/s11096-016-0257-x​

## Slide 12
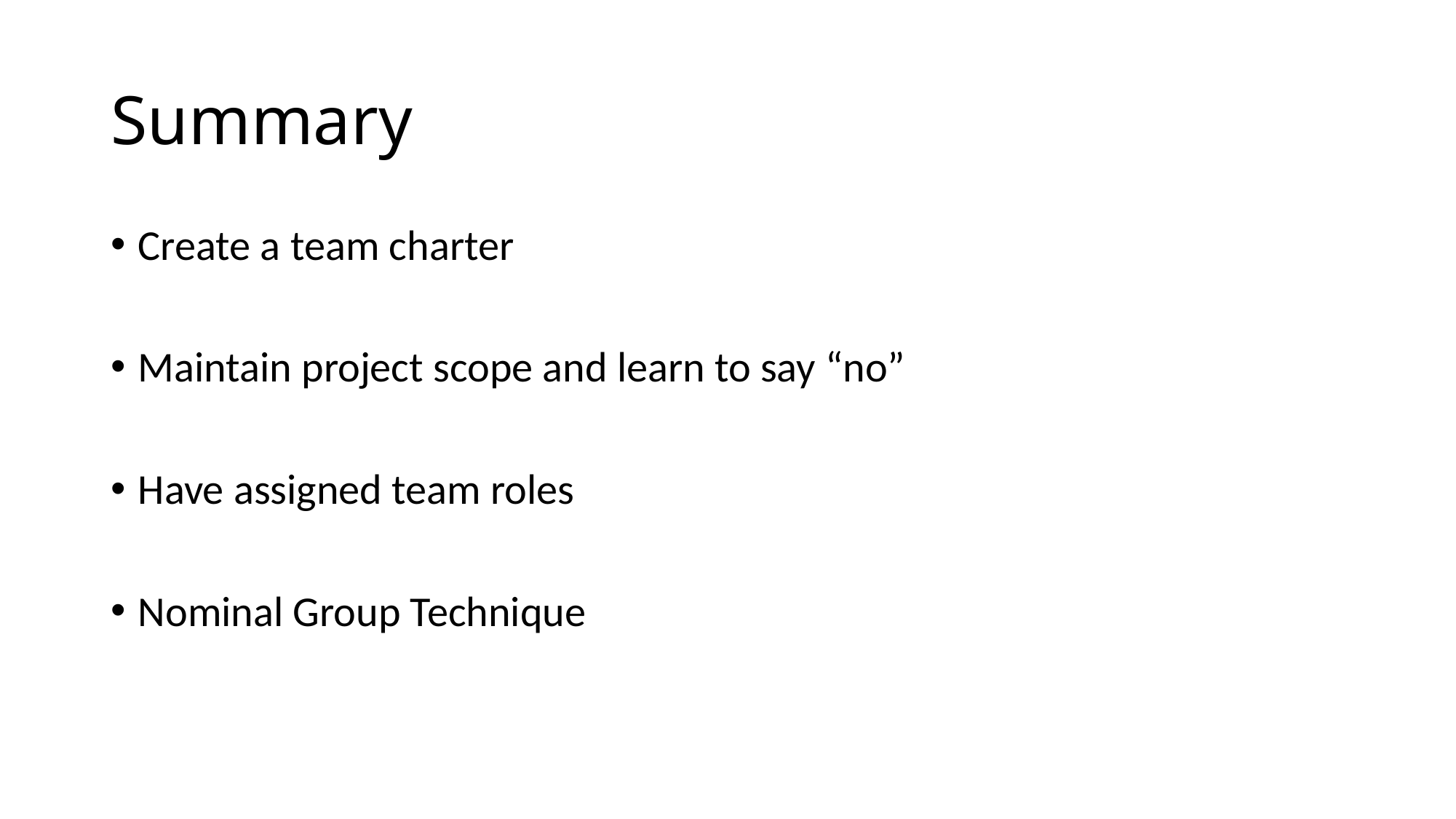

# Summary
Create a team charter
Maintain project scope and learn to say “no”
Have assigned team roles
Nominal Group Technique

## Slide 13
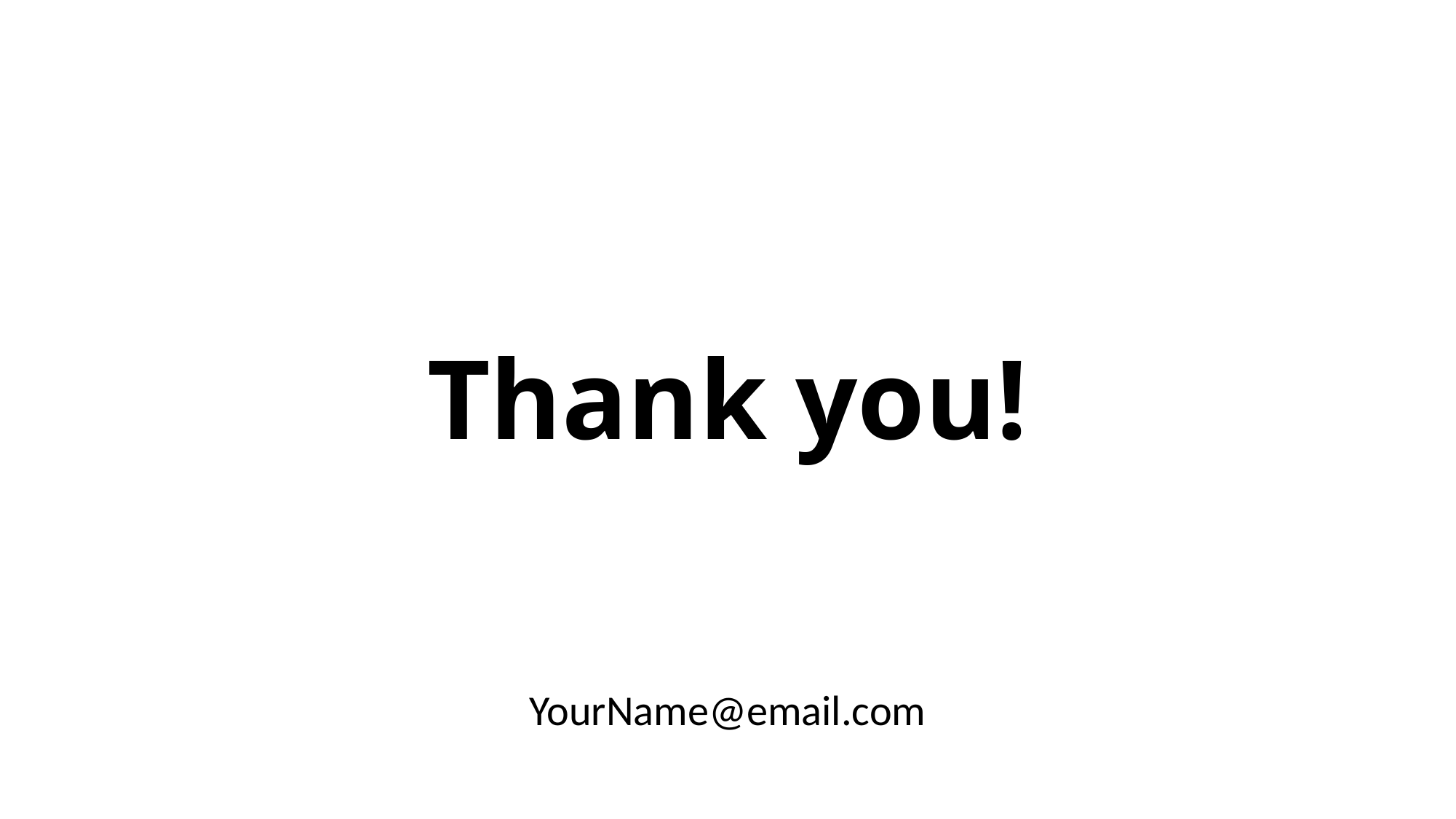

# Thank you!​
YourName@email.com​
