# Supplementary material for: A Model Curriculum for an Emergency Medicine Residency Rotation in Clinical Informatics
Source: J Educ Teach Emerg Med. 2022 Oct 15;7(4):C1–C50. doi: 10.21980/J82P9H (PMC10332664; doi:10.21980/J82P9H)
Supplement: Supplementary file 18 [file JETem-7-4-C1-AppendixE4c.docx]

Appendix E.4.b:

Leadership Form

**Leadership Agenda**

| **Date:** |  | |  | | |
| --- | --- | --- | --- | --- | --- |
| **Time:** |  | |  | | |
| **Topic:** |  | | Clinical Informatics Leadership | | |
| **Attendees:** |  | |  | | |
| **Absent:** |  | |  | | |
|  | | **Meeting Objectives:** | | | |
|  | | 1. Develop Team Charter to Solve a Problem  2. Make Decisions Using Nominal Group Technique | | | |
|  | | **Agenda** | | | |
|  | | **Name** | | **Topic** | **Time** |
|  | | Faculty | | Introduction Presentation | 10 minutes |
|  | | Learners | | Silently Write Reflections using the Team Charter | 15 minutes |
|  | | Learners | | Round Robin | 20 minutes |
|  | | Faculty/Learners | | Facilitated discussion, including grouping similar ideas and unique ideas for voting | 25 minutes |
|  | | Learners | | Silent Voting | 5 minutes |
|  | | Faculty | | Announce Results | 10 minutes |
|  | | Faculty | | Conclude | 5 minutes |
|  | | **Notes:** | | | |
|  | |  | | | |
|  | | **Next Steps:** | | | |
|  | |  | | | |

| **Team Charter** | |
| --- | --- |
| **Purpose:** Why does a team exist?  What is it expected to accomplish? |  |
| **1. Statement of Work:** Accomplish, Expected outcomes |  |
| **2. Duration:** Timeline |  |
| **3. Scope:** In/Out |  |
| **4. End result:** |  |
| **Members:** |  |
| **5:** Team, Team Leads, Members |  |
| **6. External stakeholders:** Who else might benefit or be affected by this? |  |
| **Structure and Process:** |  |
| **7. Roles and responsibilities:** |  |
| **8. Meeting plan:** |  |
| **9. Reporting plan:** |  |
| **10. Deliverables and Timetable:** |  |
| **Resources:** |  |
| **11. Financial Resources:** |  |
| **12. Technological Resources:** |  |
| **13. Support Resources:** |  |

**Nominal Group Technique**

**Individual Reflection:**

A problem was proposed by your leader. Using the team charter, silently reflect and write down possible ideas to solve the problem.

Each person verbally states his/her/their reflections which are compiled by the facilitator into one document by going around the room **without** discussion. Related items are grouped. Unique ideas are listed separately.

**Round Robin:**

After **ALL** ideas are added to the document, the facilitator leads a group discussion. A list of voting will be made for each section.

**Silent Voting:**

Each section should have a list of ideas with similar ones grouped and unique ones separated out. For voting purposes, label each idea per box alphabetically, for example: Duration: 3.a. one week, 3.b. one month, 3.c. one year

Use electronic poll to vote (MS Teams, Zoom, etc.) or have the facilitator tally votes manually by having the learners turn in their papers.

**Facilitator Announces Results**
