# Supplementary material for: A Model Curriculum for an Emergency Medicine Residency Rotation in Clinical Informatics
Source: J Educ Teach Emerg Med. 2022 Oct 15;7(4):C1–C50. doi: 10.21980/J82P9H (PMC10332664; doi:10.21980/J82P9H)
Supplement: Supplementary file 19 [file JETem-7-4-C1-AppendixF.docx]

Appendix F:

Sample Schedule

|  | Monday | Tuesday | Wednesday | Thursday | Friday |
| --- | --- | --- | --- | --- | --- |
| Week 1  Clinical Informatics Fundamentals | Rotation Introduction  Asynchronous Learning: Clinical Informatics Fundamentals | Administrative Session: Clinical Decision Support  Asynchronous Learning  Work on Appendix E.1.c. CI Fundamentals Learner Materials | Asynchronous Learning  Work on Appendix E.1.c. CI Fundamentals Learner Materials | Administrative Session: Computerized Provider Order Management  Asynchronous Learning  Work on Appendix E.1.c. CI Fundamentals Learner Materials | Small Group Discussion:  Clinical Informatics Fundamentals  Submit and discuss Appendix E.1.c. CI Fundamentals Learner Materials |
| Week 2  Improving Care Delivery and Outcomes | Asynchronous Learning: Improving Care Delivery and Outcomes  Review  Appendix E.2.b. Care Delivery Outcomes CDS Form  Review Appendix B Project Proposal Assignment | Administrative Session: Quality & Utilization  Work on  Appendix E.2.b. Care Delivery Outcomes CDS Form  Work on Appendix B Project Proposal Assignment | Administrative Session: Medical Informatics  Work on  Appendix E.2.b. Care Delivery Outcomes CDS Form  Work on Appendix B Project Proposal Assignment | Asynchronous Learning  Work on  Appendix E.2.b. Care Delivery Outcomes CDS Form  Work on  Appendix B Project Proposal Assignment | Small Group Discussion:  Improving Care Delivery and Outcomes.  Submit and discuss Appendix E.2.b. Care Delivery Outcomes CDS Form  Discuss and submit drafts of Appendix B Project Proposal Assignment |
| Week 3  Health Information Systems, Data Governance, and Data Analytics | Asynchronous Learning: Health Information Systems, Data Governance, and Data Analytics  Review Appendix E.3c. Data Analytics Governance Learner Material | Administrative Session: Physician Advisory Council  Work on  Appendix E.3c. Data Analytics Governance Learner Material  Work on Appendix B. Project Proposal Assignment | Administrative Session: Analytics Council  Work on  Appendix E.3c. Data Analytics Governance Learner Material  Work on Appendix B. Project Proposal Assignment | Asynchronous Learning  Work on  Appendix E.3c. Data Analytics Governance Learner Material  Work on Appendix B. Project Proposal Assignment | Small Group Discussion: Health Information Systems, Data Governance, and Data Analytics  Submit and discuss  Appendix E.3c. Data Analytics Governance Learner Material  Discuss and submit updated drafts of Appendix B Project Proposal Assignment |
| Week 4  Leadership & Professionalism | Asynchronous Learning: Leadership & Professionalism  Review  Appendix E.4.b. Leadership Form  Work on Appendix B Project Proposal Assignment | Asynchronous Learning: Leadership & Professionalism  Work on Appendix B Project Proposal Assignment | Administrative Session: Process Excellence  Small Group Discussion: Leadership & Professionalism  Discuss and submit updated drafts of Appendix B Project Proposal Assignment | Administrative Session: Clinical Pathways  Work on Appendix B Project Proposal Assignment | Present Work on Appendix B Project Proposal Assignment  to Leadership and other Learners |
