# Supplementary material for: A Model Curriculum for an Emergency Medicine Residency Rotation in Clinical Informatics
Source: J Educ Teach Emerg Med. 2022 Oct 15;7(4):C1–C50. doi: 10.21980/J82P9H (PMC10332664; doi:10.21980/J82P9H)
Supplement: Supplementary file 20 [file JETem-7-4-C1-AppendixG.docx]

Appendix G:

Sample Survey

**Clinical Informatics Rotation Evaluation**

Resident Name: ___________________ CI Rotation Faculty: ___________________________

Resident Program: _________________ Rotation Dates: _______________________________

Resident Level: __________________

This course was a valuable use of my elective time:

| Strongly Disagree | Disagree | Neutral | Agree | Strongly Agree |
| --- | --- | --- | --- | --- |
| 1 | 2 | 3 | 4 | 5 |

Comments:

I achieved the learning objectives:

| Strongly Disagree | Disagree | Neutral | Agree | Strongly Agree |
| --- | --- | --- | --- | --- |
| 1 | 2 | 3 | 4 | 5 |

Comments:

This rotation helped me understand Clinical Informatics:

| Strongly Disagree | Disagree | Neutral | Agree | Strongly Agree |
| --- | --- | --- | --- | --- |
| 1 | 2 | 3 | 4 | 5 |

Comments:

*Thank you to JT Finnell for his feedback on a revision of this document. Thank you to William Hersh for his educational materials. The corresponding authors would like to be contacted for further collaboration if you use this curriculum.* Carrie.Baker@ketteringhealth.org *and* Benjamin.Slovis@jefferson.edu *Please include the subject: “JET EM Resident Clinical Informatics.” Thank you!*
